# Targeting Nrf2/PHKG2 axis to enhance radiosensitivity in NSCLC

**DOI:** 10.1038/s41698-024-00629-3

**Published:** 2024-08-21

**Authors:** Fushi Han, Shuzhen Chen, Kangwei Zhang, Kunming Zhang, Meng Wang, Peijun Wang

**Affiliations:** 1 Department of Medical Imaging, Tongji Hospital, School of Medicine, Tongji University, Shanghai, 200065 China; 2 Institute of Medical Imaging Artificial Intelligence, Tongji University School of Medicine, Shanghai, 200065 China; 3 Department of Nuclear Medicine, Tongji Hospital, Tongji University School of Medicine, Shanghai, 200065 P. R. China; 4 Department of Internal Medicine, Tongji Hospital, Tongji University School of Medicine, Shanghai, 200065 P. R. China; 5 Department of Radiotherapy, Tongji Hospital, Tongji University School of Medicine, Shanghai, 200065 P. R. China

**Keywords:** Cancer epigenetics, Non-small-cell lung cancer

## Abstract

While ferroptosis shows promise in anti-cancer strategy, the molecular mechanisms behind this process remain poorly understood. Our research aims to highlight the regulation of radiotherapy-induced ferroptosis in non-small cell lung cancer (NSCLC) *via* the NRF2/PHKG2 axis-mediated mechanism. To identify ferroptosis-associated genes associated with radioresistance in NSCLC, this study employed high-throughput transcriptome sequencing and Lasso risk regression analysis. Clinical samples were analyzed to confirm PHKG2 expression changes before and after radiotherapy. The study further examined ferritinophagy-related factors, intracellular iron levels, mitochondrial function, and ferroptosis in NSCLC cells undergoing radiation exposure to explore the effect of PHKG2 on radiosensitivity or radioresistance. The research also demonstrated the transcriptional inhibition of PHKG2 by NRF2 and created in situ transplantation tumor models of NSCLC to examine the role of NRF2/PHKG2 axis in NSCLC radiosensitivity and resistance in vivo. The Lasso risk regression model that incorporated ferroptosis-associated genes effectively predicted the prognosis of patients with NSCLC. Radiotherapy-sensitive tissues exhibited an increased expression of PHKG2. Overexpression of PHKG2 led to elevated intracellular iron levels by promoting ferritinophagy and increased mitochondrial stress-dependent ferroptosis induced by radiotherapy. PHKG2 transcription repression was achieved through NRF2. The FAGs-Lasso risk regression model can accurately predict the prognosis of NSCLC patients. Targeting Nrf2 upregulates the expression of PHKG2 and reverses radiotherapy resistance in NSCLC by promoting iron autophagy and inducing mitochondrial dysfunction, thereby increasing radiotherapy sensitivity.

## Introduction

Non-small cell lung cancer (NSCLC) is one of the leading causes of cancer-related deaths worldwide^1^. It accounts for the majority of all lung cancers and generally has a poor prognosis^2^. Therefore, it is essential to study NSCLC and find practical prognostic tools and treatment strategies^3^. Radiotherapy has an essential place in the treatment of lung cancer. However, the prevalence of radioresistance in NSCLC patients has led to unsatisfactory outcomes^4^. The emergence of radioresistance, leading to tumor resistance to radiation-induced cell death, is a significant factor limiting the efficacy of radiotherapy. The molecular mechanisms of radioresistance in NSCLC are complex and poorly understood^5^. Understanding the mechanisms of radioresistance is essential to develop effective therapeutic strategies to overcome it^5^.

Ferroptosis is an iron-dependent form of regulated cell death that has recently been recognized as a critical mechanism regulating cancer cell survival, including the response of NSCLC cells to radiotherapy^6^. Ferroptosis is an iron-dependent cell death distinguished from apoptosis, necrosis, and autophagy^7^. Notably, autophagy has been highlighted as driving cells toward ferroptosis^8^. Iron phagocytosis has been associated with various cancers, including NSCLC, and its regulation is a potential cancer treatment^9^. For NSCLC, the literature shows that combined treatment with low concentrations of the ferritin inducers erastin and celastrol significantly induces cell death in NSCLC cells by activating the ROS-mitophagy signaling pathway^10^. Inhibition of ferroptosis led to radioresistance in lung cancer in a hypoxic microenvironment^11^. In addition, mitochondrial dysfunction is also involved in radioresistance^12,13^.

Understanding their downstream signaling pathways and identifying the specific genes involved may provide valuable insights for identifying new prognostic markers and therapeutic targets^14^. This knowledge may enable the development of personalized therapeutic approaches that target specific molecular pathways implicated in NSCLC radioresistance, including the triggering of ferroptosis in cancer cells^5^. Considering the importance of ferroptosis in lung cancer radiotherapy, we used high-throughput transcriptome sequencing, combined with Lasso risk regression analysis of the TCGA database NSCLC dataset, to screen ferroptosis-associated genes (FAGs) that may play a critical function in NSCLC radiotherapy^15^. Based on the survival analysis results from the TCGA-NSCLC dataset, it was observed that patients with high expression of PHKG2 had significantly better survival outcomes compared to those with low expression. Therefore, we consider PHKG2 as a crucial functional annotation gene (FAG) that is involved in radiation therapy and is associated with the prognosis of NSCLC. As a result, PHKG2 was chosen as the primary focus of this study^16^. Knockdown of PHKG2 inhibited the killing ability of erastin (a ferroptosis inducer) on U-2-OS and HT1080, suggesting that PHKG2 is involved in ferroptosis^17^. Several reports suggest that high expression of PHKG2 in lung adenocarcinoma (LUAD) favors patient prognosis^18,19^. Notably, based on UCSC predictions, NRF2 (nuclear factor erythroid 2-related factor 2) was identified as a potential upstream transcription factor for PHKG2. NRF2 is an essential regulator of cellular antioxidant response, controlling the expression of several antioxidant genes, and numerous studies have shown that NRF2 plays a vital role as its repressor in the phenomenon of iron toxicity^20,21^. Metformin induces ferroptosis in lung cancer patients as a potential therapeutic drug by downregulating the Nrf2/HO-1 signaling and increasing oxidative stress levels^22^.

This study thus sought to explore FAGs in NSCLC, which may provide valuable insights into the mechanisms underlying NSCLC radioresistance and ultimately improve therapeutic strategies’ clinical efficacy. Identifying specific genes involved in cancer cell survival and apoptosis, such as PHKG2, creates new opportunities to develop personalized treatments to enhance cancer patient survival rates. Moreover, the development of accurate prognostic markers based on FAGs may help evaluate patient prognosis accurately, enabling clinicians to tailor treatment protocols accordingly. Thus, knowledge regarding FAGs’ mechanisms and their regulatory pathways in NSCLC is essential to developing more effective clinical treatments and patient outcomes.

## Results

### PHKG2 was associated with radioresistance and prognosis in NSCLC patients

Radiotherapy can kill tumor cells by inducing ferroptosis, and the combination of ferroptosis inducers with radiation therapy is a potentially effective means of treating tumors^23^. To explore feasible methods for promoting ferroptosis to overcome radiotherapy resistance in NSCLC, we examined the impact of radiation therapy on gene expression in tumor tissues of NSCLC patients. Tumor tissue samples from radiation therapy-sensitive NSCLC patients before radiation therapy (Per-Radiotherapy, Per-RT, *n* = 4) and matched tumor tissue samples after radiation therapy (Post-Radiotherapy, Post-RT, *n* = 4) were subjected to high-throughput transcriptome sequencing analysis (RNA-Seq). Using the Per-RT group as a control, we analyzed the differentially expressed genes (DEGs) by applying the |LogFC | > 1 and *P* < 0.05 criteria. As a result, we identified 5447 DEGs, consisting of 2507 upregulated genes and 2940 downregulated genes in the irradiated tumor tissue (Supplementary Fig. 1A, B).

**Fig. 1:**
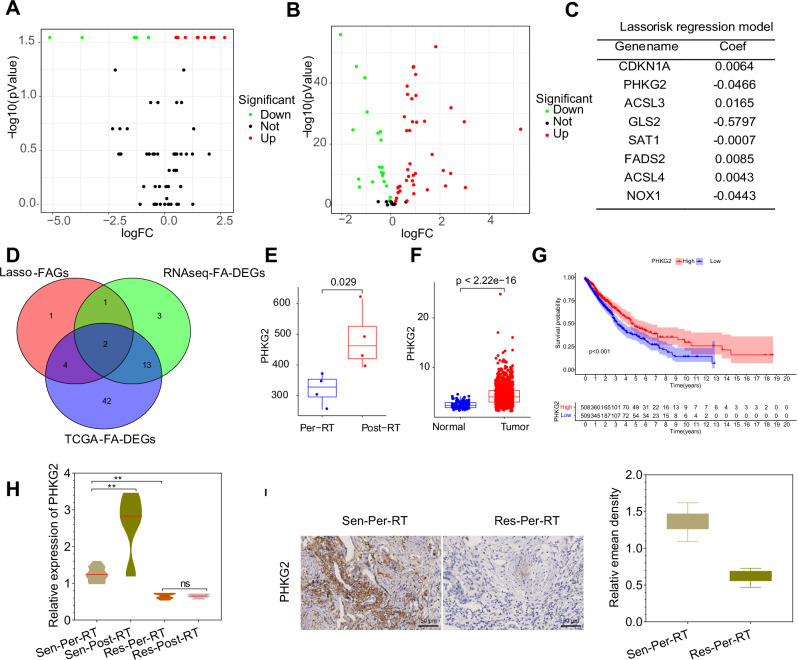
Expression of PHKG2 in NSCLC tissues of radiotherapy-sensitive and resistant patients. **A** Volcano plot depicting differential expression of FAGs between the Per-RT group (*n* = 4) and the Post-RT group (*n* = 4) based on RNAseq data, with red indicating upregulated genes and green indicating downregulated genes; **B** Volcano plot illustrating differential expression of FAGs between the Normal group (*n* = 108) and the Tumor group (*n* = 1041) using TCGA-NSCLC data, with red indicating upregulated genes and green indicating downregulated genes; **C** Genes and their Coef values used for constructing the FAGs-Lasso regression model; **D** Venn diagram displaying the intersection between RNAseq-FA-DEGs, TCGA-FA-DEGs, and Lasso-FAGs; **E** Expression profile of PHKG2 in RNAseq data; **F** Expression profile of PHKG2 in TCGA-NSCLC data; **G** Patient survival curve showing the relationship between PHKG2 expression and clinical outcomes based on TCGA-NSCLC data; **H** RT-qPCR measurement of PHKG2 expression in NSCLC tissues (*n* = 10 per group); **I** Immunohistochemical staining of PHKG2 expression in radiotherapy-sensitive (*n* = 10) and radiotherapy-resistant (*n* = 10) NSCLC tissues, with representative images on the left and quantification in the form of a bar graph on the right (scale bar = 100 μm). ^ns^*p* > 0.05, *p < 0.05, **p < 0.01, ***p < 0.001.

GO and KEGG enrichment analyses showed that these DEGs were mainly enriched in biological processes such as axon development, cell-cell adhesion via plasma-membrane, axonogenesis, and signaling pathways such as transcriptional misregulation in cancer, systemic lupus erythematosus and cell cycle (Supplementary Fig. 1C, D). Subsequently, we extracted the expression data of FAGs (70/71 FAGs) from RNAseq data as described in the Methods section^24–26^. The results of the differential analysis showed that a total of 19 FAGs were differentially expressed between the pre-radiotherapy tumor tissues and post-radiotherapy tumor tissues (ferroptosis associated-DEGs, RNAseq-FAG-DEGs), among which 14 FAGs were upregulated, and 5 were downregulated in the post-radiotherapy tumor tissues (Fig. 1A, Supplementary Fig. 1E).

To obtain FAGs associated with prognosis in NSCLC patients, we constructed a Lasso risk regression model based on transcriptomic data from the TCGA database for LUAD and LUSC. LUAD and LUSC transcriptomic and clinical data were obtained from the TCGA website. The TCGA-NSCLC transcriptomic data were obtained by combining the two sets of data. A batch correction was performed using the R software “sva” package, and the correction effect was examined by principal component analysis (PCA). The results showed that the corrected data were more evenly distributed than the pre-correction data, without a significant batch effect, which could be used for subsequent analysis (Supplementary Fig. 2A). Subsequently, we extracted the expression data of FAGs (71/71) from the corrected matrix (TCGA-NSCLC; *n*_ormal_ = 108, *n*_tumor_ = 1041) and performed differential analysis. The results revealed that a total of 61 FAGs were differentially expressed (TCGA-FA-DEGs) between adjacent standard samples and NSCLC tumor samples, of which 41 were upregulated and 20 were downregulated in tumor samples (Fig. 1B, Supplementary Fig. 2B).

Further, we performed univariate Cox analysis (Supplementary Fig. 2C) on 71 FAGs extracted from TCGA-NSCLC and selected 8 FAGs with *p* < 0.1 in the results as candidate genes for the Lasso risk regression model (CDKN1A, PHKG2, ACSL3, GLS2, SAT1, FADS2, ACSL4 and NOX1). Based on the candidate genes, we plotted the model regression coefficients and a cross-validation plot (Supplementary Fig. 2D, E) to screen the genes used for model construction. The results showed that the model had the best accuracy when the number of incorporated genes was 8 (the first dashed line on the left of the cross-validation plot). Therefore, we selected all 8 genes (Lasso-FAGs) for Lasso model construction (Fig. 1C). The FAGs-Lasso model illustrated that GLS2 (coefficient = −0.5797), PHKG2 (coefficient = −0.0466), NOX1 (coefficient = −0.0443) and SAT1 (coefficient = −0.0007) were favorable for patient prognosis (lower risk score), while high expression of ACSL4 (coefficient = 0.0043), CDKN1A (coefficient = 0.0064), FADS2 (coefficient = 0.0085) and ACSL3 (coefficient = 0.0165) were unfavorable for patient prognosis (higher risk score). Based on the risk score calculated by the FAGs-Lasso model, we assigned the patients into two groups: high-risk and low-risk groups (grouped by 50% *vs*. 50% of sample cases) and plotted survival and ROC curves. The survival curves displayed that the patients’ survival was by the expectation of the Lasso model (Supplementary Fig. 2F); the ROC curves showed that the ROC curves based on risk score had a good AUC (AUC = 0.607) and could better distinguish the prognosis of patients (Supplementary Fig. 2G), indicating that the FAGs-Lasso risk regression model was helpful for prognosis evaluation of NSCLC patients.

To obtain critical FAGs involved in radiotherapy and associated with NSCLC prognosis, we took intersections of RNAseq-FAG-DEGs, TCGA-FA-DEGs and Lasso-FAGs. The results showed that ACSL3 and PHKG2 were simultaneously present in all three subsets (Fig. 1D). We extracted the expression of both genes in the RNAseq and TCGA-NSCLC datasets. In the RNAseq dataset, both ACSL3 and PHKG2 were expressed higher in the post-radiotherapy tumor tissues than in the pre-radiotherapy tumor tissues (Fig. 1E, Supplementary Fig. 3A). In the TCGA-NSCLC dataset, both were highly expressed in the tumor tissues (Fig. 1F, Supplementary Fig. 3B). Survival analysis based on the TCGA-NSCLC dataset showed that the survival of patients with high PHKG2 expression was significantly better than that of patients with low PHKG2 expression, while the relationship between ACSL3 expression and patient survival was not significant (Fig. 1G, Supplementary Fig. 3C). Therefore, we suggest that PHKG2 is the key FAGs involved in radiotherapy and associated with NSCLC prognosis.

To test the above hypothesis, we collected matched pre- and post-radiotherapy clinical tumor tissues from 20 NSCLC patients, including 10 radiotherapy-resistant and 10 radiotherapy-sensitive patients. RT-qPCR results showed that PHKG2 expression was significantly higher in the post-radiotherapy tumor tissues from radiotherapy-sensitive NSCLC patients than in the pre-radiotherapy tumor tissues from radiotherapy-resistant NSCLC patients; the PHKG2 expression in the tissues of 8/10 patients was elevated after radiotherapy, while there was no significant change in PHKG2 levels between the pre-radiotherapy and post-radiotherapy tumor tissues from radiotherapy-resistant NSCLC patients (Fig. 1H, Supplementary Fig. 3D, E). In addition, RT-qPCR and immunohistochemistry results displayed that PHKG2 expression was significantly higher in the pre-radiotherapy tumor tissues from radiotherapy-sensitive NSCLC patients than in those from radiotherapy-resistant NSCLC patients (Fig. 1H, I), suggesting that high PHKG2 expression correlated with patients’ radiosensitivity. Collectively, PHKG2 shared a correlation with radioresistance and prognosis in NSCLC patients.

### PHKG2 promoted radiotherapy-induced ferroptosis in NSCLC cells

**Fig. 2:**
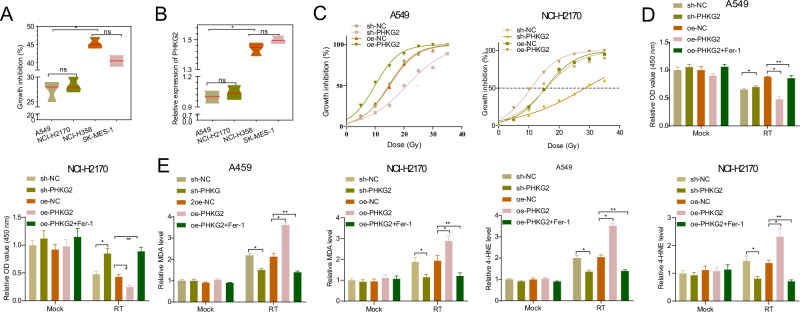
Effect of PHKG2 expression on radiotherapy-induced ferroptosis in NSCLC cells. **A** CCK-8 assay to detect the growth inhibition of NSCLC cell lines A549, NCI-H2170, NCI-H358 and SK-MES-1 by radiotherapy treatment. **B** RT-qPCR to detect the mRNA expression of PHKG2 in NSCLC cell lines A549, NCI-H2170, NCI-H358 and SK-MES-1. **C** LD50 curve for radiotherapy treatment in the presence of sh-NC, oe-NC, sh-PHKG2 or oe-PHKG2. **D** CCK-8 assay to detect the effect of single-dose radiotherapy treatment on cell viability of A549 and NCI-H2170 cell line treated with oe-PHKG2. **E** Expression of ferroptosis markers MDA and 4-HNE in A549 and NCI-H2170 cell line after single-dose radiotherapy treatment. All cell experiments were independently repeated three times. ^ns^*p* > 0.05, *p < 0.05, **p < 0.01, ***p < 0.001.

To investigate the relationship between PHKG2 expression and radiotherapy, we selected human LUAD cell lines (A549 and NCI-H358) and human and LUSC cell lines (NCI-H2170 and SK-MES-1) for in vitro cellular experimental validation. We used ^137^Cs γ-ray to treat each cell line with a total dose of 10 Gy at 0.85 Gy/min. Cell samples were collected 24 h after treatment, and the growth inhibition rate of each cell line was calculated using the sham-treated cell line (Mock group) as the control. The results showed that A549 and NCI-H2170 were more resistant to radiotherapy among the four cell lines than NCI-H358 and SK-MES-1 (Fig. 2A). Subsequently, RT-qPCR results showed that the expression of PHKG2 in A549 and NCI-H2170 was lower than that in NCI-H358 and SK-MES-1 (Fig. 2B).

To further investigate the role of PHKG2 in radiation therapy, we established cell lines with knocked-down and overexpressed PHKG2 using A549 and NCI-H2170 as parental lines. Knockdown validation results demonstrated that both sh-PHKG2-1 and sh-PHKG2-2 exhibited efficient knockdown effects. Therefore, we chose sh-PHKG2-1 (sh-PHKG2) with a more substantial knockdown effect for subsequent experiments (Fig. 2C, Supplementary Fig. 4A). The overexpression validation results found a significant increase in PHKG2 expression in the presence of oe-PHKG2 treatment (Supplementary Fig. 4B). We also validated the knockdown and overexpression effects at the protein level (Supplementary Fig. 4C), confirming the successful establishment of A549 and NCI-H2170 cell lines with knocked-down and overexpressed PHKG2, respectively. LD50 curves for the irradiation treatments of sh-NC, oe-NC, sh-PHKG2, and oe-PHKG2 were plotted, and the following results were obtained: no significant difference in LD50 values was observed between the sh-NC and oe-NC groups in A549 and NCI-H2170 cells; a significant increase in LD50 values was observed in both A549 and NCI-H2170 cells in the sh-PHKG2 group, while a significant decrease in LD50 values was observed in both cell lines in the oe-PHKG2 group (Fig. 2D). It showed that the expression of PHKG2 helped to increase the sensitivity of A549 and NCI-H2170 to radiotherapy and reversed the radioresistance.

To investigate whether PHKG2 regulates the sensitivity of A549 to radiotherapy through ferroptosis, we examined the cell line viability and its ferroptosis under different treatments. The assay results showed no significant differences in cell viability and content of ferroptosis markers (MDA and 4-HNE) in A549 cells treated with sh-NC, oe-NC, sh-PHKG2 or oe-PHKG2 before radiotherapy treatment. After radiotherapy treatment, the MDA and 4-HNE content was markedly decreased in response to PHKG2 knockdown, accompanied by enhancement of cell viability; opposite trends were found in the presence of PHKG2 overexpression (Fig. 2D, E). Further, ferroptosis inhibitor Fer-1 could resist the cell-killing effect of radiotherapy and inhibit radiotherapy-induced ferroptosis (Fig. 2D, E).

The above results suggest that changes in PHKG2 expression do not significantly affect the viability and ferroptosis of NSCLC cells but can promote radiotherapy-induced ferroptosis in NSCLC cells, thereby increasing radiosensitivity and reversing radioresistance.

### PHKG2 promoted ferritinophagy to upregulate intracellular iron levels

**Fig. 3:**
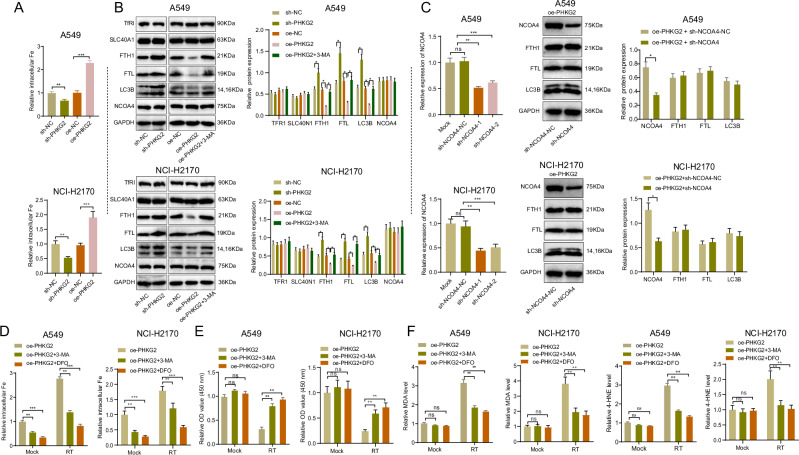
Regulation of ferritinophagy by PHKG2. **A** Comparison of intracellular iron levels in A549 cells and NCI-H2170 treated with sh-PHKG2 or oe-PHKG2. **B** Western blot detection of expression of proteins related to iron level regulation in A549 and NCI-H2170 cells treated with sh-PHKG2 or oe-PHKG2. **C** Validation of NCOA4 knockdown in oe-PHKG2-treated A549 and NCI-H2170 cell lines (left) and the effect of NCOA4 knockdown on the expression of ferritinophagy-related proteins (right). **D**–**F** Effects of 3-MA and DFO treatment on intracellular iron levels (**D**), cell viability (**E**), and ferroptosis (**F**) in oe-PHKG2-treated A549 and NCI-H2170 cells after sham radiotherapy or radiotherapy treatment. All cell experiments were independently repeated three times. ^ns^*p* > 0.05, *p < 0.05, **p < 0.01, ***p < 0.001.

Related studies have shown that PHKG2 can promote ferroptosis by upregulating intracellular iron ion levels^27^. To this end, we examined the relationship between PHKG2 expression and intracellular iron levels. The results showed that compared to the NC group, the sh-PHKG2 group exhibited a significant decrease in iron levels in A549 and NCI-H2170 cells, while the oe-PHKG2 group showed a significant increase in levels of free iron ions in A549 and NCI-H2170 cells (Fig. 3A), which is consistent with the results of previous studies. To investigate through which pathway PHKG2 affects intracellular iron levels, we examined protein expression of transferrin receptor protein 1 (TFR1), ferroportin-1/solute carrier family 40 member 1 (SLC40A1), Ferritin heavy chain (FTH1) and Ferritin light chain (FTL) in the presence of in sh-NC, oe-NC, sh-PHKG2 or oe-PHKG2. Western blot showed that the expression of FTH and FTL was increased in the presence of PHKG2 knockdown but decreased upon PHKG2 overexpression; neither PHKG2 knockdown nor PHKG2 overexpression significantly changed the expression of TFR1 and SLC40N1 (Fig. 3B). Previous studies have indicated that NCOA4 mediates ferritinophagy^28^. The Western blot results demonstrated that in the A549 and NCI-H2170 cells of the sh-PHKG2 group, the expression of FTH and FTL increased compared to the sh-NC group. In contrast, the expression of FTH and FTL decreased in the oe-PHKG2 group compared to the oe-NC group. However, there were no significant differences in the expression of TFR1 and SLC40N1 among the A549 and NCI-H2170 cells in each group (Fig. 3B). Furthermore, disruption of NCOA4 expression had no significant effect on the downregulation of FTH1 and FTL expression or the ferritinophagy induced by PHKG2 overexpression (Fig. 3C). Therefore, ferritinophagy mediated by PHKG2 and NCOA4 are independent of each other.

Furthermore, we investigated the relationship between PHKG2-mediated iron autophagy and radiotherapy resistance in NSCLC cells. A549 and NCI-H2170 cell lines overexpressing PHKG2 were treated with the autophagy inhibitor 3-MA or the iron chelator DFO, followed by radiotherapy or mock treatment. After 24 h of radiotherapy, cellular iron levels, cell viability, and the expression of iron death markers were assessed. The results demonstrated that 3-MA and DFO treatment significantly decreased iron levels in A549 and NCI-H2170 cells overexpressing PHKG2. Additionally, they suppressed radiotherapy-induced iron death and led to radiotherapy resistance in PHKG2-overexpressing cells (Fig. 3D–F).

The above results suggest that PHKG2 promotes intracellular ferritinophagy in NSCLC cells to increase intracellular iron levels and augments radiotherapy-induced ferroptosis, which enhances the radiosensitivity in NSCLC.

### PHKG2 increased radiosensitivity in NSCLC by mediating mitochondrial dysfunction

**Fig. 4:**
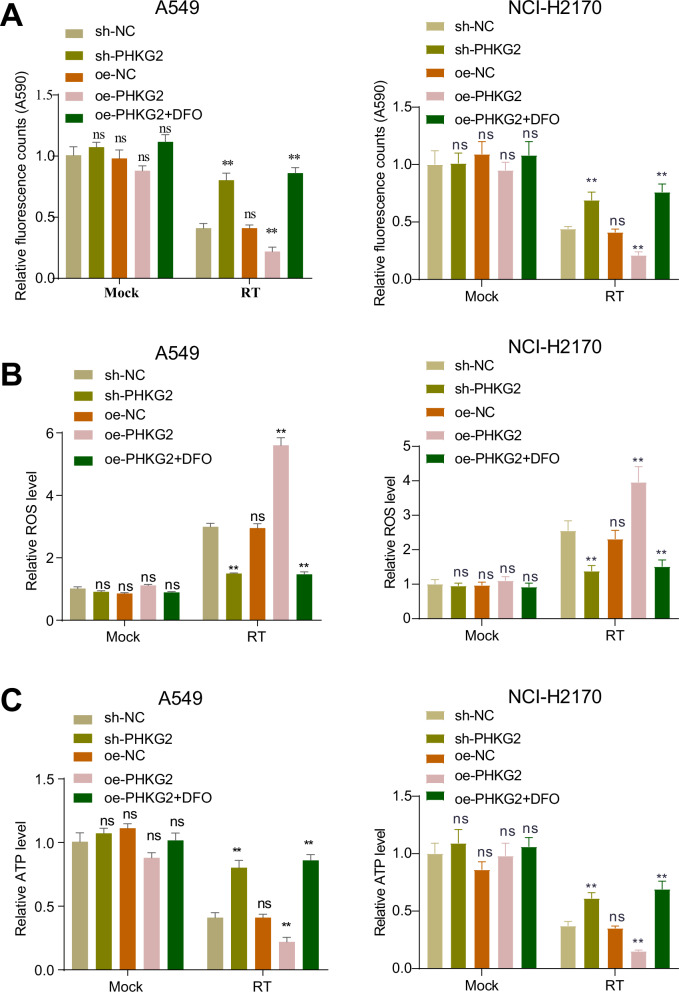
Effect of PHKG2 expression on radiotherapy-induced mitochondrial dysfunction. **A**–**C** Mitochondrial membrane potential (**A**), mitochondrial ROS (**B**) and intracellular ATP (**C**) levels in NSCLC cell lines treated with sh-PHKG2, oe-PHKG2 or oe-PHKG2 + DFO after radiotherapy or sham radiotherapy. All cell experiments were independently repeated three times. ^ns^*p* > 0.05, *p < 0.05, **p < 0.01, ***p < 0.001.

One of the characteristics of ferroptosis is mitochondrial wrinkling and dysfunction. To investigate whether PHKG2 can mediate mitochondrial dysfunction to promote radiotherapy-induced ferroptosis, we examined the mitochondrial function of A549 and NCI-H2170 cell lines with different levels of PHKG2 before and after radiotherapy treatment. The results showed no significant change in mitochondrial function in the A549 and NCI-H2170 cells with PHKG2 knockdown or overexpression before radiotherapy. However, after radiotherapy, the A549 and NCI-H2170 cells with PHKG2 overexpression showed significant mitochondrial dysfunction, as evidenced by a decrease in mitochondrial membrane potential, increased mitochondrial ROS, and a decline in intracellular ATP. Knockdown of PHKG2 or treatment of A549 and NCI-H2170 cells overexpressing PHKG2 with DFO could inhibit the mitochondrial dysfunction (Fig. 4A-C).

The above results indicate that overexpression of PHKG2 could not induce significant mitochondrial dysfunction in NSCLC cells but could promote radiotherapy-induced mitochondrial dysfunction and thus enhance their radiosensitivity.

### NRF2 negatively regulated PHKG2 transcription

To identify the upstream regulatory pathway of PHKG2, we conducted a prediction of the transcription factors for PHKG2 using UCSC. Within the predicted results, we discovered a transcriptional regulatory relationship between the iron-death-associated gene NFE2L2 (encoding the Nrf2 protein, a transcription factor) and PHKG2 (Supplementary Fig. 5). Nrf2 is a transcription factor known to regulate iron death and chemotherapy resistance mechanisms in NSCLC, as confirmed by previous extensive research^29–32^.

**Fig. 5:**
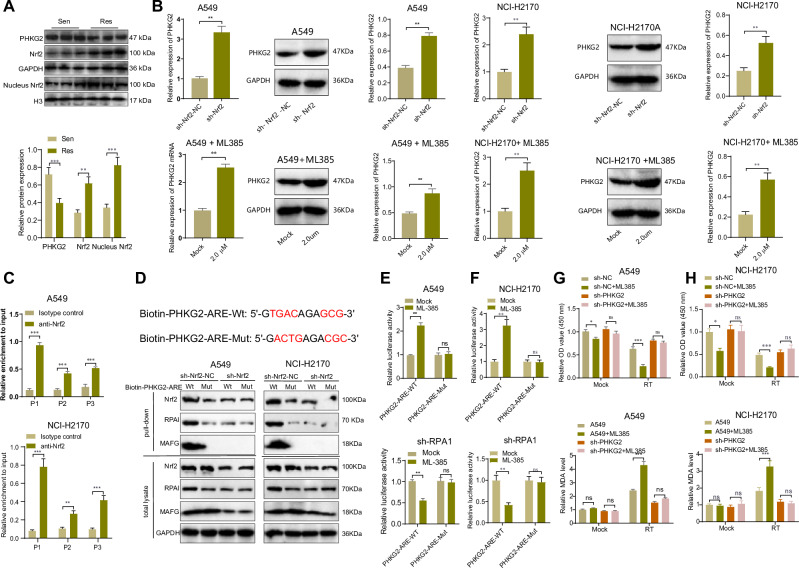
Effect of NRF2 inhibition on PHKG2 expression and sensitivity of NSCLC cells to radiotherapy. **A** Western blot to detect the expression of PHKG2 and NRF2 in NSCLC tissues of radiotherapy-sensitive and radiotherapy-resistant patients. **B** RT-qPCR and Western blot to detect the change of PHKG2 expression in A549 cells after NRF2 knockdown or inhibition. **C** ChIP-qPCR to detect potential binding sites between NRF2 and the promoter region of PHKG2. **D** Predicted PHKG2-ARE sequence and its Mut sequence. **E** DNA-pull down assay to verify the potential binding sites of NRF2 to the PHKG2 promoter region. **F** Dual luciferase assay to verify the transcriptional repression of PHKG2 by the NRF2-RPA1 complex. **G** CCK-8 assay to detect the effect of NRF2 inhibition on radiosensitivity in sh-PHKG2. **H** Effect of NRF2 inhibition on the level of ferroptosis marker MDA after radiotherapy in the presence of sh-PHKG2. All cell experiments were independently repeated three times. ^ns^*p* > 0.05, *p < 0.05, **p < 0.01, ***p < 0.001.

To validate the hypothesis above, we initially examined the expression levels of Nrf2 and PHKG2 in NSCLC tissues. Since Nrf2 functions as a transcription factor, we assessed the protein expression of Nrf2 and PHKG2 in the whole tissue and the nuclear expression of Nrf2. Western blot results revealed that both whole and nuclear Nrf2 exhibited low expression levels in radiation-sensitive tissues, while PHKG2 showed high expression in the whole tissue. In contrast, radiation-resistant tissues displayed high expression levels of both whole tissue and nuclear Nrf2, along with low expression of PHKG2 in the whole tissue. Notably, the expression of Nrf2 and PHKG2 in the whole tissue exhibited a negative correlation trend (Fig. 5A).

Furthermore, we further impaired the functionality of Nrf2 using lentiviral interference and the addition of inhibitors. RT-qPCR and Western blot analysis revealed that both shRNA treatments effectively decreased the expression of Nrf2 in A549 and NCI-H2170 cells (Supplementary Fig. 6A), and sh-NRF2-1 with optimal efficiency was used for subsequent experiments (noted as sh-NRF2). ML-385 inhibited NRF2 activation while decreasing its protein expression^33^. We established concentration gradients of 0 μM, 2 μM, and 4 μM to examine the inhibitory effects of ML-385 on Nrf2 expression in A549 and NCI-H2170 cells. RT-qPCR results revealed that ML-385 effectively suppressed Nrf2 expression levels in both A549 and NCI-H2170 cells, exhibiting a concentration-dependent pattern (Supplementary Fig. 6B), and we finally chose a concentration of 2 μM for the subsequent experiments. Subsequently, we assessed the expression of PHKG2 in A549 and NCI-H2170 cells following treatment with sh-Nrf2 and ML-385. RT-qPCR and Western blot analysis revealed a significant increase in PHKG2 expression in cells treated with sh-Nrf2 and ML-385 (when compared to the sh-Nrf2-NC group and Mock group). This suggests a potential negative regulatory role of Nrf2 in the expression of PHKG2 (Fig. 5B). Meanwhile, we constructed NCI-H358 cell lines with NRF2 overexpression, and RT-qPCR results showed that PHKG2 expression was significantly lower in response to NRF2 overexpression (Supplementary Fig. 6C), further verifying the negative regulatory effect of NRF2 on PHKG2.

The transcriptional function of NRF2 can be achieved by forming a complex with MAFG, which in turn binds to the ARE upstream of the target gene^34,35^. Replication protein A 70 kDa DNA-binding subunit (RPA1) competes with MAFG to bind to NRF2, and when RPA1 forms a complex with NRF2, it exerts the function of transcriptional repression^32,36^. We predicted the binding sites of NRF2 to the PHKG2 promoter region (NC_000016.10:30746425-30748425, Homo sapiens) through the JASPAR database and identified three potential binding sites with high scores (Supplementary Fig. 6D). Moreover, the ChIP-qPCR results in Supplementary Table 1 demonstrated that NRF2 was highly enriched at the P1 sequence (Fig. 5C). Since the core sequence of ARE is considered to be 5’-TGA(C/T)nnnGC(A/G)-3’, we mutated “TGAC” and “GCG” in the P1 sequence to eliminate their ARE function (Fig. 5D). DNA pull-down assay using Wt ARE (PHKG2-ARE-Wt) or Mut ARE (PHKG2-ARE-Mut) biotinylated DNA probes showed that there was specific binding between NRF2 and MAFG with PHKG2-ARE-Wt, but not with PHKG2-ARE-Mut (Fig. 5E). Subsequently, we constructed luciferase reporter plasmids containing PHKG2-ARE-Wt and PHKG2-ARE-Mut, which were then transfected into A549 cells. Following transfection, the cells were treated with ML-385, and a dual luciferase reporter assay was performed to measure the luciferase activity. The results revealed that ML-385 treatment significantly enhanced the luciferase activity of PHKG2-ARE-Wt while showing no significant impact on the luciferase activity of PHKG2-ARE-Mut. Moreover, after knocking down RPA1 expression (Supplementary Fig. 6E), the luciferase activity of PHKG2-ARE-Wt decreased upon treatment with ML-385, indicating a shift from negative regulation to positive regulation. Furthermore, we observed in TCGA-LUAD data that patients with KEAP1 mutations had significantly lower expression of PHKG2 compared to patients with wild-type KEAP1 (Supplementary Fig. 6F). Under physiological conditions, KEAP1 can interact with Nrf2 to prevent its nuclear translocation and transcriptional regulatory function^37^. Based on these findings, we hypothesize that Nrf2 may act as a transcription factor for PHKG2, regulating its expression and thereby contributing to ferroptosis and NSCLC radioresistance post-radiotherapy. This finding further validates the transcriptional inhibitory role of the Nrf2-RPA1 complex on PHKG2 (Fig. 5F).

To investigate the role of the Nrf2-PHKG2 axis in radiotherapy resistance, we treated sh-NC and sh-PHKG2 cell lines with the Nrf2 inhibitor ML-385 and subjected them to mock radiotherapy or radiotherapy. By assessing cell viability and occurrence of ferroptosis, we observed that ML-385 significantly increased the radiosensitivity of A549 and NCI-H2170 cells and enhanced post-radiotherapy ferroptosis. Notably, these effects were less pronounced in the sh-PHKG2 group (Fig. 5G, H).

The above results suggest that NRF2 negatively regulates PHKG2 expression, and targeted inhibition of NRF2 can promote ferroptosis after radiotherapy by upregulating PHKG2, thereby reversing NSCLC radioresistance.

### Targeting the NRF2/PHKG2 axis reversed radioresistance in NSCLC

**Fig. 6:**
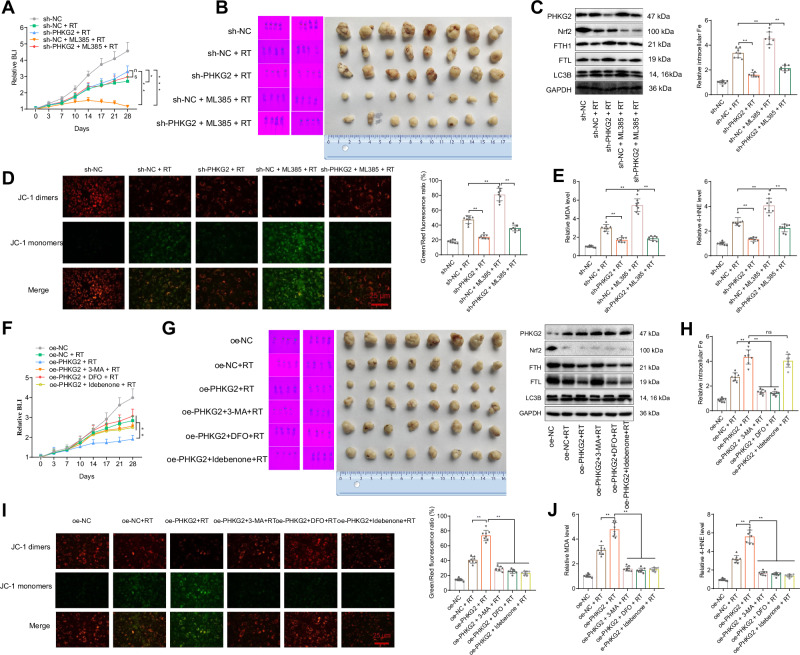
Effect of the NRF2/PHKG2 axis on radiosensitivity/radioresistance in NSCLC. **A**–**E** In vivo animal experiments exploring the modulation of NRF2/PHKG2 axis on radiosensitivity/radioresistance in NSCLC (*n* = 8). **A** Growth of the in situ transplantation tumor. **B** The PHKG2, NRF2, FTH1, FTL and LC3B expression. **C** The iron levels in NSCLC tissues. **D** JC-1 staining to detect mitochondrial membrane potential in NSCLC tissues and its quantitative map. **E** The expression of ferroptosis markers MDA and 4-HNE in NSCLC tissues. **F**–**J** In vivo animal experiments to explore ferritinophagy, cell iron levels and mitochondrial dysfunction in PHKG2-promoted ferroptosis after radiotherapy (*n* = 8). **F** Growth of the in situ transplantation tumor. **G** The expression of PHKG2, NRF2, FTH1, FTL and LC3B. H, The iron levels in NSCLC tissues. **I** JC-1 staining to detect mitochondrial membrane potential in NSCLC tissues and its quantitative map. **J** The expression of ferroptosis markers MDA and 4-HNE in NSCLC tissues. ^ns^*p* > 0.05, *p < 0.05, **p < 0.01, ***p < 0.001.

To investigate the role of the NRF2/PHKG2 axis in regulating the sensitivity/resistance to NSCLC radiotherapy, we constructed an NSCLC in situ transplantation model as described in the Methods section and treated mice with sh-NC, sh-NC + radiotherapy, sh-PHKG2 + radiotherapy, sh-NC + ML385 + RT or sh-PHKG2 + ML385 + radiotherapy. Bioluminescence intensity was measured twice weekly using small animal live imaging to assess tumor growth; mice were euthanized 28 days after injection, and tumor tissues were taken to detect relevant indexes. The experimental results showed that the knockdown of PHKG2 significantly improved the radioresistance in NSCLC, as demonstrated by the significantly higher tumor growth rate and larger experimental endpoint tumor size under radiotherapy treatment (Fig. 6A). Meanwhile, consistent with the in vitro results, the ferritinophagy (Fig. 6B), intra-tissue iron levels (Fig. 6C) and ferroptosis marker levels (Fig. 6E) were notably lower in the presence of PHKG2 knockdown under radiotherapy treatment accompanied by superior mitochondrial function (low level of mitochondrial depolarization) (Fig. 6D), suggesting that the effect of knockdown of PHKG2 on NSCLC radioresistance was related to ferritinophagy and mitochondria-dependent ferroptosis. In addition, exogenous administration of ML-385 to inhibit the expression and function of NRF2 could suppress the radioresistance, as demonstrated by a significant decrease in tumor growth rate and experimental endpoint tumor size under radiotherapy treatment, along with marked increases in PHKG2, ferritinophagy and iron levels in NSCLC tissues, mitochondrial dysfunction and elevated expression of ferroptosis markers in tissues. However, ML-385 treatment failed to reverse the radioresistance in the in situ transplantation model with PHKG2 knockdown, suggesting that the reversal of radioresistance by NRF2 was achieved through modulation of PHKG2 (Fig. 6A–E).

Further, to verify the role of ferritinophagy, cellular iron levels and mitochondrial dysfunction in PHKG2-promoted ferroptosis after radiotherapy, we constructed the NSCLC in situ transplantation tumor model again as described in the Methods section, followed by radiotherapy or/and drug treatment 7 days after tumor cell injection: oe-NC, oe-NC + radiotherapy, oe-PHKG2 + radiotherapy, oe-PHKG2 + DFO + radiotherapy and oe-PHKG2 + idebenone + radiotherapy. The experimental results were consistent with the expected results: PHKG2 overexpression significantly increased the ferritinophagy after radiotherapy and increased iron levels and mitochondrial dysfunction in the tissues after radiotherapy, which promoted the ferroptosis after radiotherapy and augmented the sensitivity of NSCLC to radiotherapy. In contrast, exogenous treatment with 3-MA, DFO or idebenone markedly inhibited these trends (Fig. 6F, J), indicating that PHKG2-mediated post-radiotherapy ferroptosis depends on ferritinophagy and mitochondrial stress. We also found that DFO and idebenone treatment did not significantly affect radiotherapy-induced ferritinophagy (Fig. 6G). Idebenone treatment had no significant effect on intracellular iron levels after radiotherapy (Fig. 6H), but 3-MA, DFO and idebenone treatment all inhibited mitochondrial dysfunction after radiotherapy (Fig. 6I), which suggests that mitochondrial dysfunction after radiotherapy may be autophagy or iron level-dependent.

The above results suggest that targeted inhibition of NRF2 or upregulation of PHKG2 both reversed the radioresistance and increased the radiosensitivity in NSCLC, the process of which is associated with ferritinophagy and mitochondrial dysfunction.

## Discussion

Recently, FAGs have gained significant attention in the field of cancer research as they have been shown to play a vital role in regulating cancer cell survival and response to various cellular stresses, including radiotherapy^38^. In our study, we identified a role for PHKG2 in radiosensitivity and prognosis of NSCLC by predicting high-throughput transcriptome sequencing and validating radiotherapy-sensitive tissues/cells. Furthermore, we found that PHKG2 promoted ferritin phagocytosis to increase intracellular iron levels (at FTH and FTL levels) and enhanced radiotherapy-induced ferroptosis, enhancing radiosensitivity in NSCLC. As a driver gene of ferroptosis, PHKG2 was also observed to be downregulated in higher stages of LUAD^18^. ophiopogonin-B (an inducer of autophagy in NSCLC cells) increases the expression of PHKG2, which contributes to the induction of ferroptosis in NSCLC, as evidenced by alterations in intracellular iron and mitochondrial membrane potential^39^. As previously described, PHKG2 knockdown inhibited the ability of erastin, a ferroptosis inducer, to kill U-2-OS and HT1080^17^. It should be noted that in the present study, we demonstrated that ferritin phagocytosis mediated by PHKG2 and NCOA4 is independent of each other and that the facilitative role of PHKG2 in radiosensitivity of NSCLC is associated with radiotherapy-induced mitochondrial dysfunction^40^. Understanding the regulatory mechanisms and downstream signaling pathways of FAGs in NSCLC cells may provide valuable insights for identifying new prognostic markers and therapeutic targets, including the development of personalized therapies and ultimately improving patient prognosis^41^.

Previous studies have indicated that baicalin, a key regulator of iron death, physically interacts with Nrf2, affecting its stability through the induction of ubiquitin degradation. This interaction inhibits the expression of downstream targets of Nrf2, such as GPX4 and xCT, ultimately stimulating iron death^42^. Additionally, EF40 reduces the expression of Nrf2, consequently suppressing the cell’s defense response that relies on Nrf2. This accumulates reactive oxygen species (ROS) within the cell, causing DNA damage responses, cell cycle arrest, and apoptosis^43^. Activation of the Nrf2 signaling pathway counteracts ROS, relieving the impaired necrosis and autophagy induced by ROS^44^. These findings demonstrate that NRF2 actively supports ROS clearance to combat iron death. Furthermore, our study reveals that targeting NRF2 upregulates PHKG2, promoting ferroptosis, enhancing radiotherapy-induced mitochondrial stress-dependent iron toxicity, and increasing the sensitivity of NSCLC to radiotherapy. Both of these mechanisms play a role in NSCLC resistance to radiotherapy.

HKG2 has emerged as a key regulator of NSCLC and radioresistance^5^. The current study has identified PHKG2 as a novel mediator promoting radiotherapy-induced iron phagocytosis in NSCLC cells^45^. PHKG2 was overexpressed in radiotherapy-sensitive tissues, promoting ferritin phagocytosis, increasing intracellular iron content and enhancing radiotherapy-induced ferroptosis^39^. In addition, NRF2 was also found to function as a transcriptional repressor of PHKG2^46^. Targeting the NRF2/PHKG2 axis promotes radiotherapy-induced ferroptosis and reverse radioresistance in NSCLC cells^41^. Thus, PHKG2 provides a promising target for developing effective therapeutic strategies for treating NSCLC, particularly in regulating ferritin formation^45^. binding of RPA1 to NRF2 converts ARE-dependent transcriptional activation to ARE-NRE-dependent repression^47^. As previously described, NRF2 is a transcriptional regulator of several genes associated with iron toxicity^48^. Stabilization of NRF2 in response to USP11 led to iron toxicity to regulate the development of NSCLC^49^. Another study showed that the NRF2-RPA1 complex directly inhibits FOCAD expression and regulates the sensitivity of human NSCLC cells to ferroptosis induced by cystine deprivation via the FAK signaling pathway^47^. In addition to inhibiting ferritin formation in NSCLC, NRF2 is also thought to be involved in radioresistance in NSCLC^50^. For example, NRF2 destabilization caused by metformin treatment enhanced the radiosensitivity of NSCLC cells^51^. Previous studies have identified 2425 differentially expressed genes between A549 cells with WT and NFE2L2 gene knockdown. Through comprehensive gene ontology analysis across all tumors, we confirmed the crucial role of NFE2L2 in tumorigenesis. Among the 20 significantly derepressed pathways mediated by NFE2L2, 12 directly involve cancer pathways^52^. This study validates the efficacy of FAGs-Lasso risk regression in accurately predicting the prognosis of NSCLC patients. Targeting NRF2 can upregulate PHKG2, promote ferritinophagy, enhance radiation-induced mitochondrial dysfunction, and increase NSCLC sensitivity to radiotherapy. These findings align with previous research, highlighting the substantial therapeutic potential of NRF2 in NSCLC treatment. These previous studies support that targeting NRF2 has great potential in tumor therapy by regulating radiotherapy-induced iron toxicity^53^. p62-Keap1-NRF2 may regulate ferritin turnover by regulating ferritin turnover through ferritin phagocytosis^54^. zVI-NP decreases NRF2 levels, leading to mitochondrial dysfunction and intracellular oxidative stress, which results in ferroptosis in lung cancer cells^55^. Blockade of the KEAP1/NRF2 axis by Bazedoxifene facilitated mitochondrial dysfunction and increased sensitivity of osimertinib to NSCLC^56^. The present study demonstrates that the NRF2/PHKG2 axis plays a key role in ferritin phagocytosis and radiotherapy-induced mitochondrial stress-dependent iron toxicity in NSCLC.

**Fig. 7:**
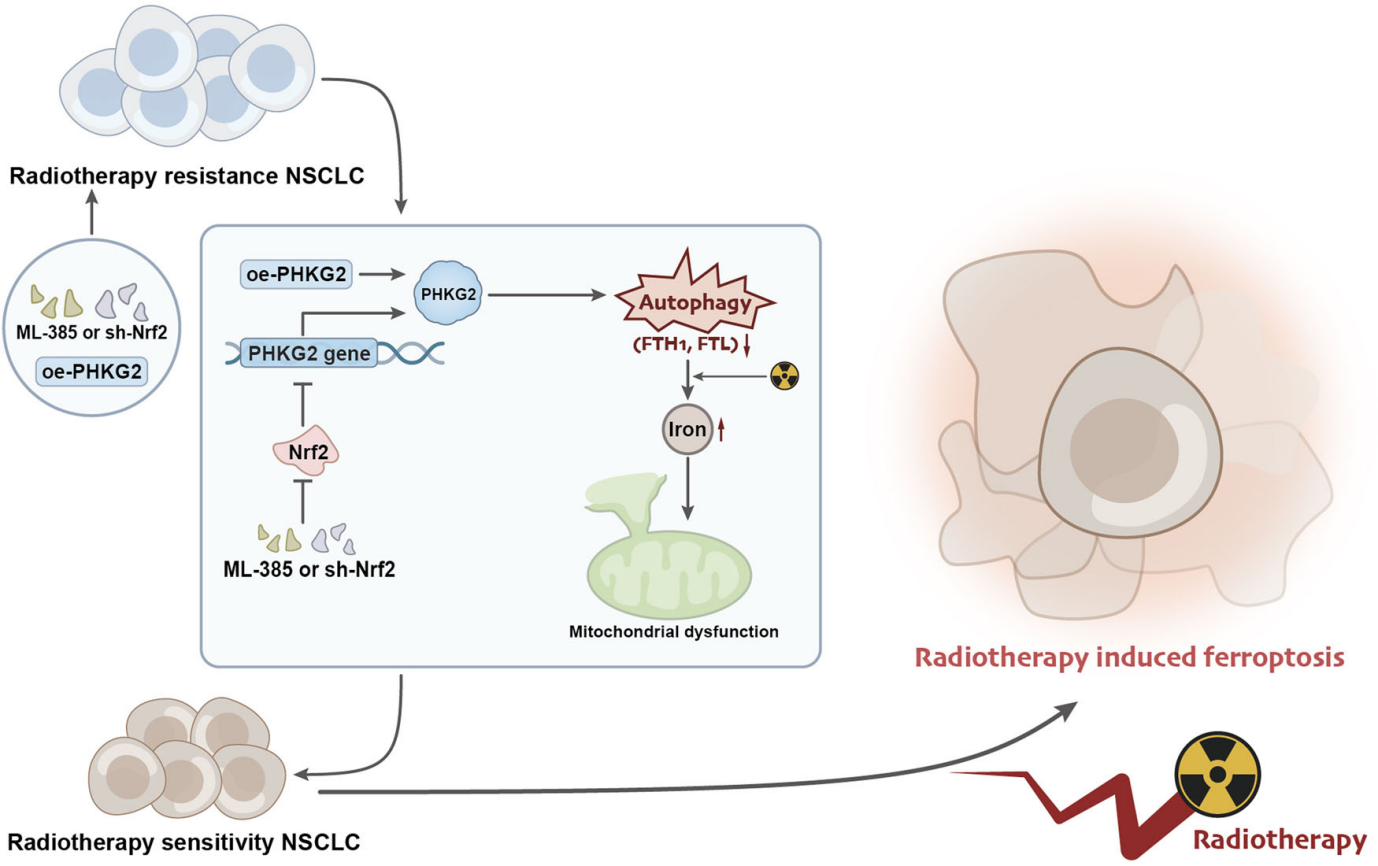
Molecular mechanism of NRF2/PHKG2 axis involved in radiosensitivity/radioresistance in NSCLC cells. Targeting NRF2 upregulates PHKG2 to promote ferritinophagy and enhance radiotherapy-induced mitochondrial stress-dependent ferroptosis, which increases the sensitivity of NSCLC to radiotherapy (The diagram was created using Biorender.).

In conclusion, it can be preliminarily inferred that FAGs-Lasso risk regression can more accurately predict the prognosis of NSCLC patients. Moreover, targeting NRF2 can upregulate PHKG2, promote ferritin phagocytosis, enhance radiation-induced mitochondrial dysfunction, thereby increasing the sensitivity of NSCLC to radiation therapy (Fig. 7). The current study’s contribution regarding the significance of FAGs and PHKG2 in regulating NSCLC’s radiation sensitivity and resistance provides a promising outlook for developing effective radiotherapy strategies. Identifying the regulatory pathways of these genes offers targets for drug development and further clinical research, paving the way for creating new personalized treatment methods to improve efficacy. However, the study’s focus on a limited number of gene regulatory pathways necessitates further investigation, including whether NRF2’s inhibitory effect on PHKG2 transcription applies to other tumor samples and the roles of other genes in radiation resistance regulation. The use of non-human sample models with NSCLC cell lines presents limitations in terms of clinical significance. Nevertheless, recognizing the importance of understanding the roles of FAGs and PHKG2 and their regulatory mechanisms in NSCLC radiation resistance holds rich potential for further investigation and development to enhance cancer treatment outcomes. In summary, researching FAGs and PHKG2 provides a valuable avenue for advancing personalized precision medicine approaches for cancer patients, ultimately improving clinical outcomes and quality of life for patients.

## Methods

### Ethical approval

The study was conducted under the approval (Approval No. SBKT-2023-052) of the Ethics Committee of Tongji Hospital, Tongji University School of Medicine and has complied with all relevant ethical regulations including the Declaration of Helsinki and the Guidance of the Ministry of Science and Technology (MOST) for the Review and Approval of Human GeneticResources. Written informed consent was obtained from all patients. All procedures in the animal experiment were approved by the Animal Ethics Committee of Tongji Hospital, Tongji University School of Medicine (Approval No. 2023-DW-SB-018) and conducted by the *Guide for the Care and Use of Laboratory Animals*.

### FAG expression acquisition from TCGA NSCLC transcriptome data

Transcriptomic and patient clinical data for LUAD and lung squamous carcinoma (LUSC) were obtained from the TCGA database. The LUAD dataset contained 598 samples (normal = 59, tumor = 539), and the LUSC dataset contained 551 samples (normal = 49, tumor = 502). The two data sets were combined, and the batch effect was removed using the R software “sva” package to obtain the NSCLC data matrix (total = 1149; normal = 108, tumor = 1041). The expression data of FAGs in the TCGA NSCLC expression matrix were extracted, and the heat map of differential expression of FAGs was drawn using the R software “heatmap”.

A total of 71 FAGs were collected from previous studies^24–26^, including ABCC1, ACACA, ACO1, ACSF2, ACSL3, ACSL4, AIFM2, AKR1C1, AKR1C2, AKR1C3, ALOX12, ALOX15, ALOX5, ATP5MC3, CARS1, CBS, CD44, CDKN1A, CHAC1, CISD1, CISD2, CRYAB, CS, DHODH, DPP4, EMC2, FADS2, FANCD2, FDFT1, FTH1, FXN, G6PD, GCH1, GCLC GCLM, GLS2, GOT1, GPX4, GSS, HMGCR, HMOX1, HSBP1, HSPA5, HSPB1, IREB2, KEAP1, LPCAT3, MT1G, NCOA4, NFE2L2, NFS1, NOX1, NQO1, PEBP1, PGD, PHKG2, PLA2G6, POR, PROM2, PTGS2, RARRES2, RPL8, SAT1, SCD, SLC1A5, SLC7A11, SQLE, STEAP3, TFRC, TP53, and ZEB1. The protein-protein interaction (PPI) network of these FAGs is demonstrated in Supplementary Fig. 7.

### FAGs-based Lasso risk regression model construction

The genes with *p* < 0.1 were selected to construct the Lasso risk regression model. The risk score was calculated, and the survival curves were plotted. ROC curves were plotted using the “survivalROC” package, and heat maps of gene expression and clinical relevance were delineated using the “pheatmap” package. Univariate and multivariate Cox regression analyses were performed using the “survival” package to verify whether the Lasso risk regression model was helpful in clinical diagnosis^57^.

### Sample collection

Tumor samples for our study were obtained from patients treated for NSCLC at Tongji Hospital, Tongji University School of Medicine, and all patients enrolled were first diagnosed with primary NSCLC and had not previously received any form of oncology treatment. Patient information for sequencing and validation is shown in Supplementary Table 2–3. The sequencing samples were pre- and post-radiotherapy tissue samples from 4 radiotherapy-sensitive patients, and the validation samples were pre- and post-radiotherapy tissue samples from 10 radiotherapy-sensitive and 10 radiotherapy-resistant patients. For tissue sample acquisition, in situ, NSCLC tumor tissues were obtained according to the treatment protocol^58^, and fresh samples were snap-frozen in liquid nitrogen and then stored at -80 °C for long-term storage.

### High-throughput transcriptome sample sequencing, data quality control and differential analysis

Total RNA was extracted from each sample using Trizol reagent (16096020, Thermo Fisher Scientific, Rockford, IL). RNA concentration, purity and integrity were determined using the Qubit® RNA Analysis Kit (HKR2106-01, Biogene, Shanghai, China) from Qubit®2.0 Fluorometer® (Q33216, Life Technologies, Carlsbad, CA), a Nanometer spectrophotometer (IMPLEN) and RNA Nano 6000 analysis kits from Bioanalyzer 2100 system (5067-1511, Agilent Technologies, Palo Alto, CA), respectively. Each sample contained 3 μg of total RNA and was used as input material for RNA sample preparation. The cDNA libraries were generated using the NEBNext® UltraTM RNA Library Preparation Kit for Illumina® (E7435L, New England Biolabs, Beverly, MA) according to the manufacturer’s recommendations and quality was assessed on an Agilent Bioanalyzer 2100 system. According to the manufacturer’s instructions, index-coded samples were clustered on the cBot cluster generation system using TruSeq PE Cluster Kit v3 cBot HS (Illumina) (PE-401-3001, Illumina, San Diego, CA). After cluster generation, library preparations were sequenced on the Illumina-Hiseq 550 platform, and 125 bp/150 bp paired-end reads were generated^59,60^.

The quality of paired-end reads of raw sequencing data was checked using FastQC software v0.11.8. Pre-processing of raw data using Cutadapt software 1.18 was performed by removal of Illumina sequencing junctions and poly(A) tail sequences. Reads with >5% N content were removed by a perl script, and 70% of the reads with base mass above 20 were extracted using FASTX Toolkit software 0.0.13. Double-end sequences were repaired using BBMap software. Finally, the filtered high-quality reads were compared with the human reference genome by hisat2 software (0.7.12). Differentially expressed genes (DEGs) between Donor and pAML samples were screened by the R software “Limma” package (with a threshold of |logFC | > 1, *p*-value < 0.05). An expression heat map of DEGs was mapped using the R “heatmap” package, and a volcano plot of DEGs was mapped using the R “ggplot2” package. GO and KEGG enrichment analyses were performed using the R software “clusterProfiler” package^61,62^.

### In vitro culture of human NSCLC cell lines

For cell culture, human LUAD cell lines A549 (CCL-185, ATCC, Manassas, VA), NCI-H358 (CRL-5807, ATCC) and human LUSC cell line NCI-H2170 (CRL-5928, ATCC) were cultured using RPMI 1640 medium (11875119, Thermo Fisher Scientific) supplemented with 10% FBS (10100147 C, Thermo Fisher Scientific). The human LUSC cell line SK-MES-1 (HTB-58, ATCC) was cultured using DMEM-H (11965092, Thermo Fisher Scientific) supplemented with 10% FBS. All cells were cultured with 1% penicillin-streptomycin (15140163, Thermo Fisher Scientific) and left at 37 °C with 5% CO_2_^63^.

### Lentivirus production and transduction

Lentiviral transfection was used to construct cell lines with gene knockdown or overexpression and the corresponding control cell lines, while a Mock group only added with medium (solvent) was set up as a validation control. A549 cells were infected with lentiviral vectors harboring overexpression (oe)-negative control (NC), short hairpin RNA (sh)-NC, oe-PHKG2 and sh-PHKG2 (EGFP-labeled group, also applied to animal experiments), sh-NRF2-NC and sh-NRF2, sh-NCOA4-NC and sh-NCOA4, sh-RPA1-NC and sh-RPA1; NCI-H358 cells were infected with lentiviral vectors harboring oe-NRF2-NC and oe-NRF2. In the knockdown experiments, 2 shRNA sequences were used simultaneously, and the one with optimal efficiency was selected for subsequent experimentation. shRNA sequences are shown in Supplementary Table 4. Constructed plasmids were co-transduced into 293 T cells with the helper plasmids, followed by validation, amplification, and purification to obtain the packaging lentivirus. Sangon (Shanghai, China) provided the plasmid construction and lentivirus packaging.5 × 10^5^ cells were seeded into 6-well plates for lentivirus-mediated cell infection. When cell confluency reached 70–90%, medium containing appropriate amounts of packaging lentivirus (MOI = 10, working titer = about 5 × 10^6^ TU/mL) and 5 μg/mL polybrene (TR-1003, Merck KGaA, Darmstadt, Germany) was added to the cells for infection. After 4 h of infection, an equal amount of medium was added to dilute polybrene, and the medium was replaced with fresh medium after 24 h. After 48 h of transfection, 1 μg/mL puromycin (A1113803, Thermo Fisher Scientific) was used for resistance screening to obtain stably infected cell lines.

Cellular irradiation was performed using the X-RAD 320 Biological Irradiator (Precision X-Ray, USA) at a frequency of 0.85 Gy/min, with a single exposure and a total dose of 10 Gy. Cells were collected 24 h after irradiation for subsequent experiments. Drug treatments were initiated 24 h before irradiation (for a total treatment duration of 48 h) by adding NRF2 inhibitor ML-385 (2 μM, HY-100523)^64^, iron chelator deferoxamine (DFO) (100 μM, HY-B0988)^65^, Fer-1 (60 nm, HY-100579), or autophagy inhibitor 3-methyladenine (3-MA) (5 mM, HY-19312)^66^ to the respective culture medium^67^. All drugs were purchased from MCE (USA)^68^.

### CCK-8 assay

CCK8 kits (C0037, Beyotime, Shanghai, China) were used to detect the growth inhibition of cells by radiation therapy. Cells were seeded in 96-well culture plates, and after radiotherapy or drug treatment, 10 μL CCK-8 working solution (10% of the culture volume) was added to each well and incubated at 37 °C for 2 h. The absorbance values (A) at 450 nm were measured using a Multiskan FC microplate reader (51119080, Thermo Fisher Scientific), with three duplicated wells set up for each group; the average values were taken^68^. A sham radiotherapy group and solvent group (blank control for drugs) were set up to determine the growth inhibition rate, where the cell growth inhibition rate = 1—OD 450 nm _treatment group_ / OD 450 nm _control group_.

### Ferroptosis marker test

Ferroptosis is a form of cell death caused by the accumulation of lipid peroxidation on cell membranes; therefore, the lipid peroxidation products malondialdehyde (MDA) and 4-hydroxynonenal (4-HNE) can respond to the ferroptosis^69^. MDA content in cells or tissues was measured using the Micro MDA Assay Kit (BC0025, Solarbio, Beijing, China) and Lipid Peroxidation (4-HNE) Assay Kit (ab238538, Abcam, Cambridge, UK) to detect 4-HNE content in cells or tissues^70^.

### Iron level measurement

According to the manufacturer’s protocol, Orange FerroOrange was used to detect Fe2+ in cells. Properly treated cells were incubated with 1 μM orange FerroOrange in Hank’s Balanced Salt Solution (HBSS) at 37 °C for 30 min and observed under a confocal laser scanning microscope (Olympus Corp., Tokyo, Japan). Tissues were collected and freeze-dried, and 3 mg of tissue per sample was digested overnight in 65% concentrated nitric acid. After removing the nitric acid by heating, 1% nitric acid was added to equilibrate the volume to 1 mL. The concentration of iron in the tissues was measured by inductively coupled plasma mass spectrometry (ICP-MS, Thermo Fisher Scientific)^27^.

### Mitochondrial function assessment

Mitochondrial membrane potential was measured using the JC-1-Mitochondrial Membrane Potential Assay Kit (ab113850, Abcam). Cells were incubated with a 5 μM MitoSOX probe for 10 min at 37 °C and observed under a fluorescence microscope (Olympus Corp.) to measure mitochondrial ROS. Cellular ATP levels were measured using a firefly luciferase-based ATP assay kit. Briefly, the cells were centrifuged to remove debris, and the supernatant was added to the substrate solution. Luminescence was recorded using a microplate reader (BioTek Instruments, Winooski, VT)^27^.

### ChIP, DNA pull-down and dual luciferase assays

When the cells reached 70–80% confluence, 1% formaldehyde was added and fixed at room temperature for 10 min to cross-link the intracellular DNA with the protein. The supernatant was collected and incubated overnight at 4 °C in two tubes with the NC antibody rabbit anti-IgG (1:100, Abcam, ab172730) and the target protein-specific antibody anti-NRF2 (2 μg/test, Abcam, ab62352), respectively. The endogenous DNA-protein complex was precipitated using Protein Agarose/Sepharose, and the supernatant was aspirated and removed after brief centrifugation. ChIP-qPCR products were characterized by 3% agarose gel electrophoresis^27^.

Biotin-PHKG2-ARE-wild-type (Wt) (5’-GTGACAGAGCG-3’)/Biotin-PHKG2-ARE-mutant (Mut) (5’-GACTGAGACGC-3’) (Genecreate, Wuhan, China) labeled with 50 nM biotin were transduced into sh-NC- or sh-NRF2-treated cells for 48 h. The cells were collected and lysed, and the lysates were mixed with M-280 streptavidin magnetic beads (Merck KGaA) pre-encapsulated with RNase-free BSA and yeast tRNA (Merck KGaA, 55714), followed by incubation overnight at 4 °C with vortexing for 3 h. The beads were washed twice with pre-cooled lysis buffer, three times with low salt buffer and once with high salt buffer. Western blot was used to detect the enrichment of related proteins^71^.

The binding site of NRF2 to the promoter region of PHKG2 was predicted using the JASPAR database. Then, the Wt (5’-GTGACAGAGCG-3’) and Mut (5’-GACTGAGACGC-3’) sequences of PHKG2 were constructed separately and inserted into the pGL-3 luciferase reporter vector (Thermo Fisher Scientific, 4351372). The luciferase reporter plasmid was transfected into A549 cells or cells transduced with sh-RPA1, and the cells were collected and lysed after 48 h. Following centrifugation at 250 ×g for 3 ~ 5 min and extraction of the supernatant, the Firefly and Renilla luciferase activities were detected using the Dual Luciferase® Reporter Assay System (E1910, Promega, Madison, WI); the ratio of the activity of firefly luciferase to that of Ranilla luciferase indicated relative luciferase activity^72^.

### Immunohistochemistry

NSCLC tissue samples were fixed in 4% paraformaldehyde, dehydrated, cleared, wax-impregnated, and embedded before tissue sectioning. For immunohistochemical staining, sections were dewaxed and rehydrated, and antigen retrieval was performed according to the instructions of the primary antibody (1:200, PHKG2, Thermo Fisher Scientific, PA5-98059), followed by staining using a universal two-step detection kit (PV-9000, ZhongShan JinQiao, Beijing, China) according to the protocol provided by the manufacturer. The staining results were observed and preserved using a light microscope (CX43, Olympus). The results were analyzed semi-quantitatively using Image Pro Plus software.

### Western blot

Total cell or tissue proteins were extracted using a protein extraction kit (BB3101, Bestbio, Shanghai, China), nucleus proteins were extracted with a cell plasma protein extraction kit (Bestbio, P0027), and protein concentrations were determined with a BCA kit (Beyotime, P0012S). A 10% SDS-PAGE gel (Beyotime, P0012A) was prepared, 50 μg of protein samples were added to each well, and the proteins were electrophoresed at a constant pressure of 80 V to 120 V for 2 h. The proteins were transferred to a PVDF membrane (Merck KGaA, IPVH00010) at a constant flow of 250 mA for 90 min with the wet transfer method. The PVDF membrane was sealed with TBST containing 5% skim milk powder at room temperature for 2 h. Primary antibodies (Supplementary Table 5) were incubated overnight at 4 °C, followed by further incubation with horseradish peroxidase-conjugated goat anti-rabbit IgG (1:2000, Abcam, ab6721) or goat anti-mouse IgG (1:2000, Abcam, ab6789) at room temperature for 1 h. ECL reaction solution (Beyotime, P0018FS) was used for color development, followed by exposure in darkness and image development. All the Original WB images can be found in Supplementary Figs. 8–75.

### RT-qPCR

Total RNA from tissues and cells was extracted using Trizol (Thermo Fisher Scientific, 16096020). cDNA was obtained by reverse transcription using a reverse transcription kit (RR047A, Takara, Shiga, Japan). cDNA was prepared using a SYBR® Premix Ex TaqTM II kit (Takara, DRR081). GAPDH was used as an internal reference. The amplification curves were plotted. All RT-qPCR experiments were set up with 3 duplicated wells. The primer design is shown in Supplementary Table 6. 2^-ΔΔCt^ indicated the multiple ratios of target gene expression between the experimental and control groups.

### In situ transplantation model of NSCLC

Eighty-eight 4–5 week-old BALB/c nude mice weighing 20 ± 2 g were purchased from Vital River Laboratory Animal Technology Co., Ltd. (Beijing, China). Mice were housed in standard feeding cages at a constant room temperature of 23 ± 1 °C with 12 h light/dark cycles and free access to food and water. The mice were acclimatized and housed for 1 week before the experiment.

For NSCLC in situ transplantation tumor model construction: 2 × 10^6^ of EGFP-labeled NSCLC cells were injected transthoracically into 6 week-old BALB/c nude mice, and to confirm successful injection, bioluminescence intensity of the whole mice was immediately measured using a bioluminescence imaging system (IVIS Imaging System Xenogen, PerkinElmer, Waltham, MA). The lung fluorescence signal was examined twice weekly to monitor tumor growth and plot tumor growth curves.

On days 8, 15, and 22 after cell inoculation, partial irradiation of mouse tumors was performed using the X-RAD 320 Biological Irradiator (Precision X-Ray, USA). The rest of the body was shielded from radiation using lead clothing. Each treatment consisted of a 4 Gy dose, resulting in a total dose of 12 Gy. During irradiation, mice were anesthetized with isoflurane and fixed with metomidate. Upon awakening, they were returned to standard housing conditions. The same procedures were followed in the sham irradiation group, but the irradiation switch was not turned on. For the groups receiving drug treatment, ML-385 (30 mg/kg), DFO (200 mg/kg), 3-MA (15 mg/kg), or Idebenone (20 mg/kg, HY-N0303) [Idebenone (IDB) is an effective antioxidant] were administered through intraperitoneal injection to the mice, twice per week, starting 7 days after tumor cell injection. The control group received injections of an equal volume of physiological saline. Mice were euthanized 28 days after tumor injection (exposed to a visible euthanasia box filled with 40% carbon dioxide gas, with euthanasia gas replaced at a rate of 30% of box volume per minute, exposure for 5 min, confirming animal immobility, lack of respiration, and dilated pupils. CO_2_ was then turned off, and animals were monitored for 2–3 min to confirm death) for dissection, and tumor tissues from each group of mice were collected for subsequent experiments^73^.

In vivo experiments were performed in 2 parts as follows. A total of 40 mice were used to verify the modulatory effect of the NRF2/PHKG2 axis on the sensitivity/resistance to NSCLC radiotherapy (*n* = 8): sh-NC (inoculated with sh-NC and treated with sham radiotherapy), sh-NC + radiotherapy (inoculated with sh-NC and treated with radiotherapy), sh-PHKG2 + radiotherapy (inoculated with sh-PHKG2 and treated with radiotherapy), sh-NC + ML385 + radiotherapy (inoculated with sh-NC and treated with both ML-385 and radiotherapy) or sh-PHKG2 + ML385 + RT (inoculated with sh-PHKG2 and treated with both ML-385 and radiotherapy). Other 48 mice used to verify the role of ferritinophagy, cellular iron levels and mitochondrial dysfunction in PHKG2-promoted ferroptosis after radiotherapy were treated with oe-NC, oe-NC + radiotherapy, oe-PHKG2 + radiotherapy, oe-PHKG2 + 3-MA + radiotherapy, oe-PHKG2 + DFO + radiotherapy, and oe-PHKG2 + idebenone + radiotherapy (*n* = 8).

### Statistical analysis methods

In our study, bioinformatics results were statistically analyzed using R 4.2.1 and other results were statistically analyzed using SPSS 26.0 (IBM Corp. Armonk, NY). The measurement data were expressed as mean ± standard deviation. First, tests were conducted to assess the normality and homogeneity of variances. These tests ensured that the data followed a normal distribution and that the variances were equal. For comparisons between the two groups, we employed the independent samples *t*-test. On the other hand, we utilized a one-way analysis of variance (ANOVA) for comparisons among multiple groups. Additionally, we employed repeated measures ANOVA to account for within-subject variability when comparing multiple time points. *p* < 0.05 indicated statistical differences. The bioinformatics figures were generated using raw data and R language, while the bar graphs were plotted using GraphPad Prism 8.0. The experimental pathology and cellular images were obtained by the laboratory.

### Reporting summary

Further information on research design is available in the Nature Research Reporting Summary linked to this article.

### Supplementary information

Reporting Summary

Supplementary Information

## Data Availability

The RNA-sequencing data generated and analyzed during this study have been deposited in the National Center for Biotechnology Information (NCBI) Sequence Read Archive (SRA), a repository that is part of the International Nucleotide Sequence Database Collaboration (INSDC). The data are publicly accessible under BioProject ID PRJNA1120111. The SRA accession numbers for the data are as follows: **Per-RT Samples:** • SRR29288018 • SRR29288017 • SRR29288016 • SRR29288015 **Post-RT Samples:** • SRR29288014 • SRR29288013 • SRR29288012 • SRR29288011
