# Supplementary material for: Targeting Nrf2/PHKG2 axis to enhance radiosensitivity in NSCLC
Source: NPJ Precis Oncol. 2024 Aug 21;8:183. doi: 10.1038/s41698-024-00629-3 (PMC11339382; doi:10.1038/s41698-024-00629-3)
Supplement: Supplementary file 1 — Reporting Summary [file 41698_2024_629_MOESM1_ESM.pdf]

nature portfolio

Corresponding author(s): Peijun Wang
Last updated by author(s): Nov 30, 2023

Reporting Summary

Nature Portfolio wishes to improve the reproducibility of the work that we publish. This form provides structure for consistency and transparency in reporting. For further information on Nature Portfolio policies, see our Editorial Policies and the Editorial Policy Checklist.

Statistics

For all statistical analyses, confirm that the following items are present in the figure legend, table legend, main text, or Methods section.

n/a	Confirmed	
☐	☒	The exact sample size (*n*) for each experimental group/condition, given as a discrete number and unit of measurement
☐	☒	A statement on whether measurements were taken from distinct samples or whether the same sample was measured repeatedly
☐	☒	The statistical test(s) used AND whether they are one- or two-sided<br>*Only common tests should be described solely by name; describe more complex techniques in the Methods section.*
☐	☒	A description of all covariates tested
☐	☒	A description of any assumptions or corrections, such as tests of normality and adjustment for multiple comparisons
☐	☒	A full description of the statistical parameters including central tendency (e.g. means) or other basic estimates (e.g. regression coefficient) AND variation (e.g. standard deviation) or associated estimates of uncertainty (e.g. confidence intervals)
☐	☒	For null hypothesis testing, the test statistic (e.g. *F*, *t*, *r*) with confidence intervals, effect sizes, degrees of freedom and *P* value noted<br>*Give P values as exact values whenever suitable.*
☐	☒	For Bayesian analysis, information on the choice of priors and Markov chain Monte Carlo settings
☐	☒	For hierarchical and complex designs, identification of the appropriate level for tests and full reporting of outcomes
☐	☒	Estimates of effect sizes (e.g. Cohen's *d*, Pearson's *r*), indicating how they were calculated

Our web collection on statistics for biologists contains articles on many of the points above.

Software and code

Policy information about availability of computer code

Data collection	The article's data will be shared on reasonable request to the corresponding author.
Data analysis	The article's data will be shared on reasonable request to the corresponding author.

For manuscripts utilizing custom algorithms or software that are central to the research but not yet described in published literature, software must be made available to editors and reviewers. We strongly encourage code deposition in a community repository (e.g. GitHub). See the Nature Portfolio guidelines for submitting code & software for further information.

Data

Policy information about availability of data

All manuscripts must include a data availability statement. This statement should provide the following information, where applicable:

- Accession codes, unique identifiers, or web links for publicly available datasets
- A description of any restrictions on data availability
- For clinical datasets or third party data, please ensure that the statement adheres to our policy

The article's data will be shared on reasonable request to the corresponding author.

Research involving human participants, their data, or biological material

Policy information about studies with human participants or human data. See also policy information about sex, gender (identity/presentation), and sexual orientation and race, ethnicity and racism.

Reporting on sex and gender	Tumor samples for our study were obtained from patients treated for NSCLC at Tongji Hospital, Tongji University School of Medicine, and all patients enrolled were first diagnosed with primary NSCLC and had not previously received any form of oncology treatment. Patient information for sequencing and validation is shown in Supplementary Table 2-3.
Reporting on race, ethnicity, or other socially relevant groupings	N/A
Population characteristics	N/A
Recruitment	Tongji Hospital, Tongji University School of Medicine
Ethics oversight	The study was conducted under the approval (Approval No. SBKT-2023-052) of the Ethics Committee of Tongji Hospital, Tongji University School of Medicine.

Note that full information on the approval of the study protocol must also be provided in the manuscript.

Field-specific reporting

Please select the one below that is the best fit for your research. If you are not sure, read the appropriate sections before making your selection.

☒ Life sciences ☐ Behavioural & social sciences ☐ Ecological, evolutionary & environmental sciences

For a reference copy of the document with all sections, see nature.com/documents/nr-reporting-summary-flat.pdf

Life sciences study design

All studies must disclose on these points even when the disclosure is negative.

Sample size	Tumor samples for our study were obtained from patients treated for NSCLC at Tongji Hospital, Tongji University School of Medicine, and all patients enrolled were first diagnosed with primary NSCLC and had not previously received any form of oncology treatment. Patient information for sequencing and validation is shown in Supplementary Table 2-3.
Data exclusions	Tumor samples for our study were obtained from patients treated for NSCLC at Tongji Hospital, Tongji University School of Medicine, and all patients enrolled were first diagnosed with primary NSCLC and had not previously received any form of oncology treatment. Patient information for sequencing and validation is shown in Supplementary Table 2-3. The sequencing samples were pre- and post-radiotherapy tissue samples from 4 radiotherapy-sensitive patients, and the validation samples were pre- and post-radiotherapy tissue samples from 10 radiotherapy-sensitive and 10 radiotherapy-resistant patients. For tissue sample acquisition, in situ, NSCLC tumor tissues were obtained according to the treatment protocol 56, and fresh samples were snap-frozen in liquid nitrogen and then stored at -80? for long-term storage.
Replication	Tumor samples for our study were obtained from patients treated for NSCLC at Tongji Hospital, Tongji University School of Medicine, and all patients enrolled were first diagnosed with primary NSCLC and had not previously received any form of oncology treatment. Patient information for sequencing and validation is shown in Supplementary Table 2-3. The sequencing samples were pre- and post-radiotherapy tissue samples from 4 radiotherapy-sensitive patients, and the validation samples were pre- and post-radiotherapy tissue samples from 10 radiotherapy-sensitive and 10 radiotherapy-resistant patients. For tissue sample acquisition, in situ, NSCLC tumor tissues were obtained according to the treatment protocol 56, and fresh samples were snap-frozen in liquid nitrogen and then stored at -80? for long-term storage.
Randomization	Tumor samples for our study were obtained from patients treated for NSCLC at Tongji Hospital, Tongji University School of Medicine, and all patients enrolled were first diagnosed with primary NSCLC and had not previously received any form of oncology treatment. Patient information for sequencing and validation is shown in Supplementary Table 2-3. The sequencing samples were pre- and post-radiotherapy tissue samples from 4 radiotherapy-sensitive patients, and the validation samples were pre- and post-radiotherapy tissue samples from 10 radiotherapy-sensitive and 10 radiotherapy-resistant patients. For tissue sample acquisition, in situ, NSCLC tumor tissues were obtained according to the treatment protocol 56, and fresh samples were snap-frozen in liquid nitrogen and then stored at -80? for long-term storage.
Blinding	Tumor samples for our study were obtained from patients treated for NSCLC at Tongji Hospital, Tongji University School of Medicine, and all patients enrolled were first diagnosed with primary NSCLC and had not previously received any form of oncology treatment. Patient information for sequencing and validation is shown in Supplementary Table 2-3. The sequencing samples were pre- and post-radiotherapy tissue samples from 4 radiotherapy-sensitive patients, and the validation samples were pre- and post-radiotherapy tissue samples from 10 radiotherapy-sensitive and 10 radiotherapy-resistant patients. For tissue sample acquisition, in situ, NSCLC tumor tissues were obtained according to the treatment protocol 56, and fresh samples were snap-frozen in liquid nitrogen and then stored at -80? for long-term storage.

Reporting for specific materials, systems and methods

We require information from authors about some types of materials, experimental systems and methods used in many studies. Here, indicate whether each material, system or method listed is relevant to your study. If you are not sure if a list item applies to your research, read the appropriate section before selecting a response.

Materials & experimental systems

n/a	Involved in the study
☐	☒ Antibodies
☐	☒ Eukaryotic cell lines
☒	☐ Palaeontology and archaeology
☐	☒ Animals and other organisms
☒	☐ Clinical data
☒	☐ Dual use research of concern
☒	☐ Plants

Methods

n/a	Involved in the study
☒	☐ ChIP-seq
☒	☐ Flow cytometry
☒	☐ MRI-based neuroimaging

Antibodies

Antibodies used

The supernatant was collected and incubated overnight at 4? in two tubes with the NC antibody rabbit anti-IgG (1:100, Abcam, ab172730) and the target protein-specific antibody anti-NRF2 (2 μg/test, Abcam, ab62352), respectively.

Validation

The supernatant was collected and incubated overnight at 4? in two tubes with the NC antibody rabbit anti-IgG (1:100, Abcam, ab172730) and the target protein-specific antibody anti-NRF2 (2 μg/test, Abcam, ab62352), respectively.

Eukaryotic cell lines

Policy information about cell lines and Sex and Gender in Research

Cell line source(s)

To investigate the relationship between PHKG2 expression and radiotherapy, we selected human LUAD cell lines (A549 and NCI-H358) and human and LUSC cell lines (NCI-H2170 and SK-MES-1) for in vitro cellular experimental validation. We used 137Cs γ-ray to treat each cell line with a total dose of 10 Gy at 0.85 Gy/min. Cell samples were collected 24 h after treatment, and the growth inhibition rate of each cell line was calculated using the sham-treated cell line (Mock group) as the control. The results showed that A549 and NCI-H2170 were more resistant to radiotherapy among the four cell lines than NCI-H358 and SK-MES-1 (Figure 2A). Subsequently, RT-qPCR results showed that the expression of PHKG2 in A549 and NCI-H2170 was lower than that in NCI-H358 and SK-MES-1 (Figure 2B).

Authentication

To investigate the relationship between PHKG2 expression and radiotherapy, we selected human LUAD cell lines (A549 and NCI-H358) and human and LUSC cell lines (NCI-H2170 and SK-MES-1) for in vitro cellular experimental validation. We used 137Cs γ-ray to treat each cell line with a total dose of 10 Gy at 0.85 Gy/min. Cell samples were collected 24 h after treatment, and the growth inhibition rate of each cell line was calculated using the sham-treated cell line (Mock group) as the control. The results showed that A549 and NCI-H2170 were more resistant to radiotherapy among the four cell lines than NCI-H358 and SK-MES-1 (Figure 2A). Subsequently, RT-qPCR results showed that the expression of PHKG2 in A549 and NCI-H2170 was lower than that in NCI-H358 and SK-MES-1 (Figure 2B).

Mycoplasma contamination

To investigate the relationship between PHKG2 expression and radiotherapy, we selected human LUAD cell lines (A549 and NCI-H358) and human and LUSC cell lines (NCI-H2170 and SK-MES-1) for in vitro cellular experimental validation. We used 137Cs γ-ray to treat each cell line with a total dose of 10 Gy at 0.85 Gy/min. Cell samples were collected 24 h after treatment, and the growth inhibition rate of each cell line was calculated using the sham-treated cell line (Mock group) as the control. The results showed that A549 and NCI-H2170 were more resistant to radiotherapy among the four cell lines than NCI-H358 and SK-MES-1 (Figure 2A). Subsequently, RT-qPCR results showed that the expression of PHKG2 in A549 and NCI-H2170 was lower than that in NCI-H358 and SK-MES-1 (Figure 2B).

Commonly misidentified lines
(See ICLAC register)

To investigate the relationship between PHKG2 expression and radiotherapy, we selected human LUAD cell lines (A549 and NCI-H358) and human and LUSC cell lines (NCI-H2170 and SK-MES-1) for in vitro cellular experimental validation. We used 137Cs γ-ray to treat each cell line with a total dose of 10 Gy at 0.85 Gy/min. Cell samples were collected 24 h after treatment, and the growth inhibition rate of each cell line was calculated using the sham-treated cell line (Mock group) as the control. The results showed that A549 and NCI-H2170 were more resistant to radiotherapy among the four cell lines than NCI-H358 and SK-MES-1 (Figure 2A). Subsequently, RT-qPCR results showed that the expression of PHKG2 in A549 and NCI-H2170 was lower than that in NCI-H358 and SK-MES-1 (Figure 2B).

Animals and other research organisms

Policy information about studies involving animals; ARRIVE guidelines recommended for reporting animal research, and Sex and Gender in Research

Laboratory animals

Eighty-eight 4-5-week-old BALB/c nude mice weighing 20 ± 2 g were purchased from Vital River Laboratory Animal Technology Co., Ltd. (Beijing, China). Mice were housed in standard feeding cages at a constant room temperature of 23 ± 1°C with 12-h light/dark cycles and free access to food and water. The mice were acclimatized and housed for one week before the experiment.

Wild animals

N/A

Reporting on sex	Eighty-eight 4-5-week-old BALB/c nude mice weighing 20 ± 2 g were purchased from Vital River Laboratory Animal Technology Co., Ltd. (Beijing, China). Mice were housed in standard feeding cages at a constant room temperature of 23 ± 1°C with 12-h light/dark cycles and free access to food and water. The mice were acclimatized and housed for one week before the experiment.
Field-collected samples	Eighty-eight 4-5-week-old BALB/c nude mice weighing 20 ± 2 g were purchased from Vital River Laboratory Animal Technology Co., Ltd. (Beijing, China). Mice were housed in standard feeding cages at a constant room temperature of 23 ± 1°C with 12-h light/dark cycles and free access to food and water. The mice were acclimatized and housed for one week before the experiment.
Ethics oversight	All procedures in the animal experiment were approved by the Animal Ethics Committee of Tongji Hospital, Tongji University School of Medicine (Approval No. 2023-DW-SB-018) and conducted by the Guide for the Care and Use of Laboratory Animals.

Note that full information on the approval of the study protocol must also be provided in the manuscript.

Plants

Seed stocks	N/A
Novel plant genotypes	N/A
Authentication	N/A
