# Supplementary material for: Targeting Nrf2/PHKG2 axis to enhance radiosensitivity in NSCLC
Source: NPJ Precis Oncol. 2024 Aug 21;8:183. doi: 10.1038/s41698-024-00629-3 (PMC11339382; doi:10.1038/s41698-024-00629-3)
Supplement: Supplementary file 2 — Supplementary Information [file 41698_2024_629_MOESM2_ESM.docx]

Supplementary Table 1 Primer sequences for ChIP-qPCR

| Prediction site | Sequences (5’-3’) |
| --- | --- |
| P1 (Human) | Forward: 5’-AGTGCAGTGGTGTGATCTCA-3’ |
|  | Reverse: 5’-CGCCTGTAATCCTAGCTACTCA-3’ |
| P2 (Human) | Forward: 5’-AAGCAGAGGTAAGAGCCAGT-3’ |
|  | Reverse: 5’-TGGAATGGGGAAAGTGGAGA-3’ |
| P3 (Human) | Forward: 5’-CTGAGTCACTCCTGGCAGAA-3’ |
|  | Reverse: 5’-CAATCTTCCACCCTCTCCCA-3’ |

Note: ChIP, chromatin immunoprecipitation; qPCR, quantitative polymerase chain reaction.

Supplementary Table 2 Clinical and pathological features of 4 NSCLC patients for sequencing

| Features | Radiosensitivity (n = 4) | |
| --- | --- | --- |
|  | n | % |
| Age (years) |  |  |
| ≤55 | 1 | 25 |
| >55 | 3 | 75 |
| Gender |  |  |
| Male | 3 | 75 |
| Female | 1 | 25 |
| Smoking history |  |  |
| Yes | 4 | 100 |
| No | 0 | 0 |
| Histological |  |  |
| Adenocarcinoma | 2 | 50 |
| Squamous carcinoma | 2 | 50 |
| Differentiation |  |  |
| Moderate and High | 2 | 50 |
| Poor | 2 | 50 |
| TNM stage |  |  |
| Ⅰ | 0 | 0 |
| Ⅱ | 0 | 0 |
| Ⅲ | 3 | 75 |
| Ⅳ | 1 | 25 |

Note: NSCLC, non-small cell lung cancer; n, number; TNM, tumor-node-metastasis

Supplementary Table 3 Clinical and pathological features of 20 NSCLC patients for validation

| Features | Radiosensitivity (n = 10) | | Radioresistance (n = 10) | |
| --- | --- | --- | --- | --- |
|  | n | % | n | % |
| Age (years) |  |  |  |  |
| ≤55 | 4 | 40 | 7 | 70 |
| >55 | 6 | 60 | 3 | 30 |
| Gender |  |  |  |  |
| Male | 7 | 70 | 4 | 40 |
| Female | 3 | 30 | 6 | 60 |
| Smoking history |  |  |  |  |
| Yes | 8 | 80 | 6 | 60 |
| No | 2 | 20 | 4 | 40 |
| Histological |  |  |  |  |
| Adenocarcinoma | 5 | 50 | 6 | 60 |
| Squamous carcinoma | 5 | 50 | 4 | 40 |
| Differentiation |  |  |  |  |
| Moderate and High | 4 | 40 | 3 | 30 |
| Poor | 6 | 60 | 7 | 70 |
| TNM Stage |  |  |  |  |
| Ⅰ | 2 | 20 | 0 | 0 |
| Ⅱ | 3 | 30 | 0 | 0 |
| Ⅲ | 5 | 50 | 0 | 0 |
| Ⅳ | 0 | 0 | 10 | 100 |

Note: NSCLC, non-small cell lung cancer; n, number; TNM, tumor-node-metastasis

Supplementary Table 4 shRNA sequences

| shRNA | Sequences (5’-3’) |
| --- | --- |
| sh-PHKG2-1 | 5’-CGCCAGAGATCCTTAAATGCT-3’ |
| sh-PHKG2-2 | 5’-CTAATGATCCTGCTACCCTCT-3’ |
| sh-NRF2-1 | 5’-CCGGCATTTCACTAAACACAA-3’ |
| sh-NRF2-2 | 5’-GCTCCTACTGTGATGTGAAAT-3’ |
| sh-NCOA4-1 | 5’-TCAGCAGCTCTACTCGTTATT-3’ |
| sh-NCOA4-2 | 5’-ACTCTTGTTTATCGAAGTATA-3’ |
| sh-RPA1-1 | 5’-CCCTAGAACTGGTTGACGAAA-3’ |
| sh-RPA1-2 | 5’-CGTGCTGTCTTCAAGCACTAT-3’ |

Note: sh-, short hairpin RNA; PHKG2, phosphorylase kinase catalytic subunit gamma 2; NRF2, NFE2L2 (NRF2) NFE2 like bZIP transcription factor 2; NCOA4, nuclear receptor coactivator 4; RPA1, [replication protein A1](https://www.ncbi.nlm.nih.gov/gene/6117).

Supplementary Table 5 Manufacturer information of antibodies used for Western blot

| Antibody | Manufacturer | Cat. | Dilution ratio |
| --- | --- | --- | --- |
| PHKG2 (Human) | Thermo Fisher | PA5-98059 | 1:1000 |
| NRF2 (Human) | Abcam | ab62352 | 1:1000 |
| TFR1 (Human) | Abcam | ab214039 | 1:1000 |
| SLC40A1 (Human) | Abcam | ab239583 | 1:500 |
| FTH1 (Human) | Abcam | ab75972 | 1:2000 |
| FTL (Human) | Abcam | ab109373 | 1:10000 |
| LC3B (Human) | Abcam | ab192890 | 1:2000 |
| NCOA4 (Human) | Abcam | ab62495 | 1:100 |
| RPA1 (Human) | Abcam | ab79398 | 1:2000 |
| MAFG (Human) | Abcam | ab154318 | 1:500 |
| H3 (Human) | Abcam | ab1791 | 1:1000 |
| GAPDH (Human) | Abcam | ab8245 | 1:500 |

Note: PHKG2, phosphorylase kinase catalytic subunit gamma 2; NRF2, NFE2L2 (NRF2) NFE2 like bZIP transcription factor 2; TFR1, transferrin receptor; SLC40A1, solute carrier family 40 member 1; FTH1, ferritin heavy chain 1; FTL, ferritin light chain; LC3B, light chain 3 beta; NCOA4, nuclear receptor coactivator 4; RPA1, [replication protein A1](https://www.ncbi.nlm.nih.gov/gene/6117); MAFG, MAF bZIP transcription factor G; H3, histocompatibility 3; GAPDH, glyceraldehyde-3-phosphate dehydrogenase.

Supplementary Table 6 Primer sequences for RT-qPCR

| Gene | Sequences (5’-3’) |
| --- | --- |
| PHKG2 (Human) | Forward: 5’-GAGTTTTACCAGAAGTACGACCC-3’ |
|  | Reverse: 5’-GTAGCTCGATGAACACAACGG-3’ |
| NCOA4 (Human) | Forward: 5’-GAGGTGTAGTGATGCACGGAG-3’ |
|  | Reverse: 5’-GACGGCTTATGCAACTGTGAA-3’ |
| NRF2 (Human) | Forward: 5’-TCCAGTCAGAAACCAGTGGAT-3’ |
|  | Reverse: 5’-GAATGTCTGCGCCAAAAGCTG-3’ |
| GAPDH (Human) | Forward: 5’-TGTTCGTCATGGGTGTGAAC-3’ |
|  | Reverse: 5’-ATGGCATGGACTGTGGTCAT-3’ |

Note: RT-qPCR, reverse transcription-quantitative polymerase chain reaction; PHKG2, phosphorylase kinase catalytic subunit gamma 2; NCOA4, nuclear receptor coactivator 4; NRF2, NFE2L2 (NRF2) NFE2 like bZIP transcription factor 2; GAPDH, glyceraldehyde-3-phosphate dehydrogenase.

**
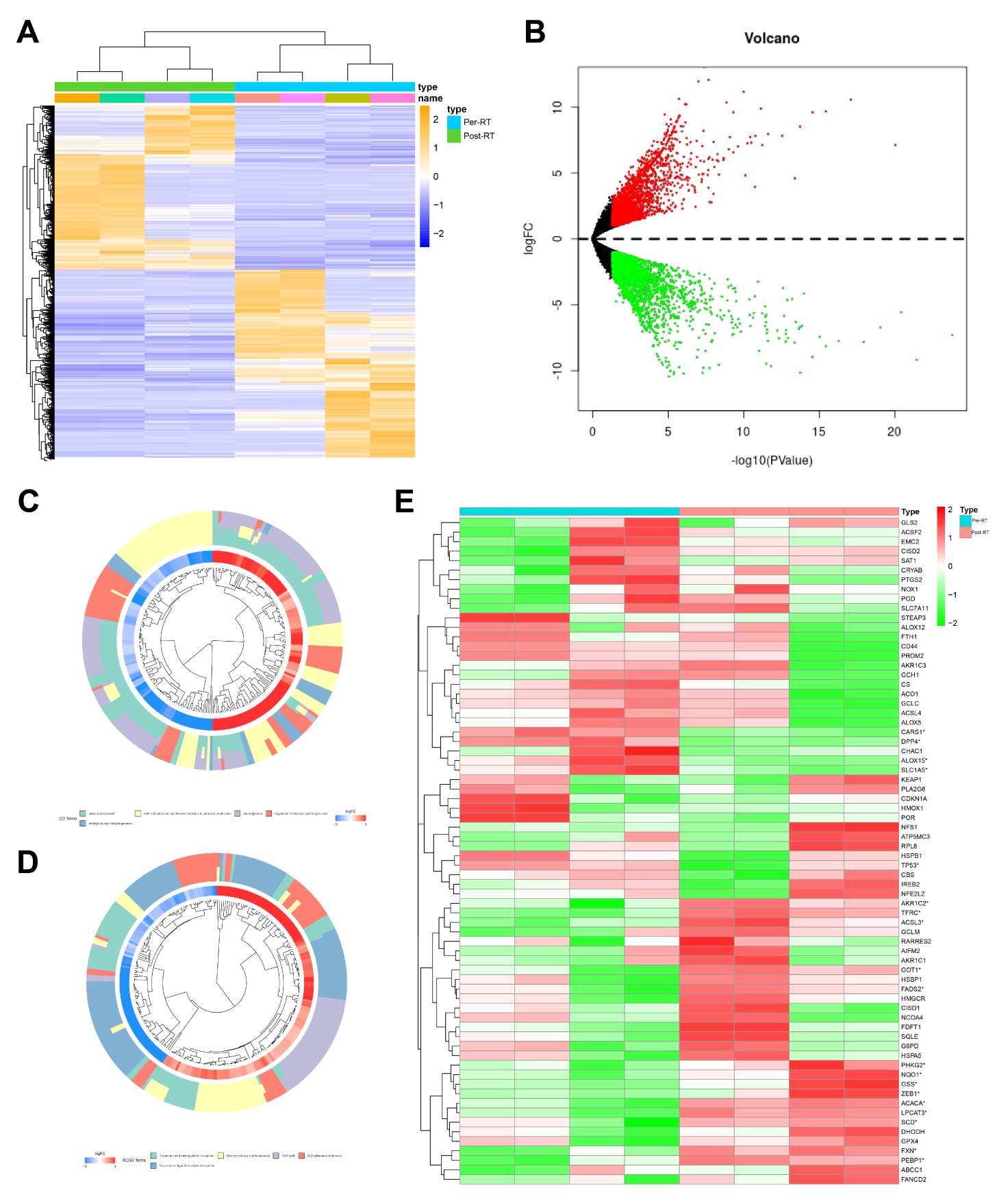
**

Supplementary Figure 1 DEGs obtained through RNAseq and their functional enrichment analysis. A, Expression heat map of DEGs in the pre-radiotherapy group (n = 4) versus post-radiotherapy group (n = 4) in RNAseq, with color scale at the top right (yellow represents up-regulated expression and blue represents down-regulated expression). B, Volcano map of DEGs in the pre-radiotherapy group versus post-radiotherapy group, where red represents up-regulated genes, green represents down-regulated genes, and black represents genes not differentially expressed. C & D, Results of GO and KEGG enrichment analyses of DEGs in pre- and post-radiotherapy groups. E, Expression heat map of FAGs in RNAseq between pre-radiotherapy group (n = 4) and post-radiotherapy group (n = 4), with color scale on the upper right (red represents up-regulated expression and green represents down-regulated expression).

**
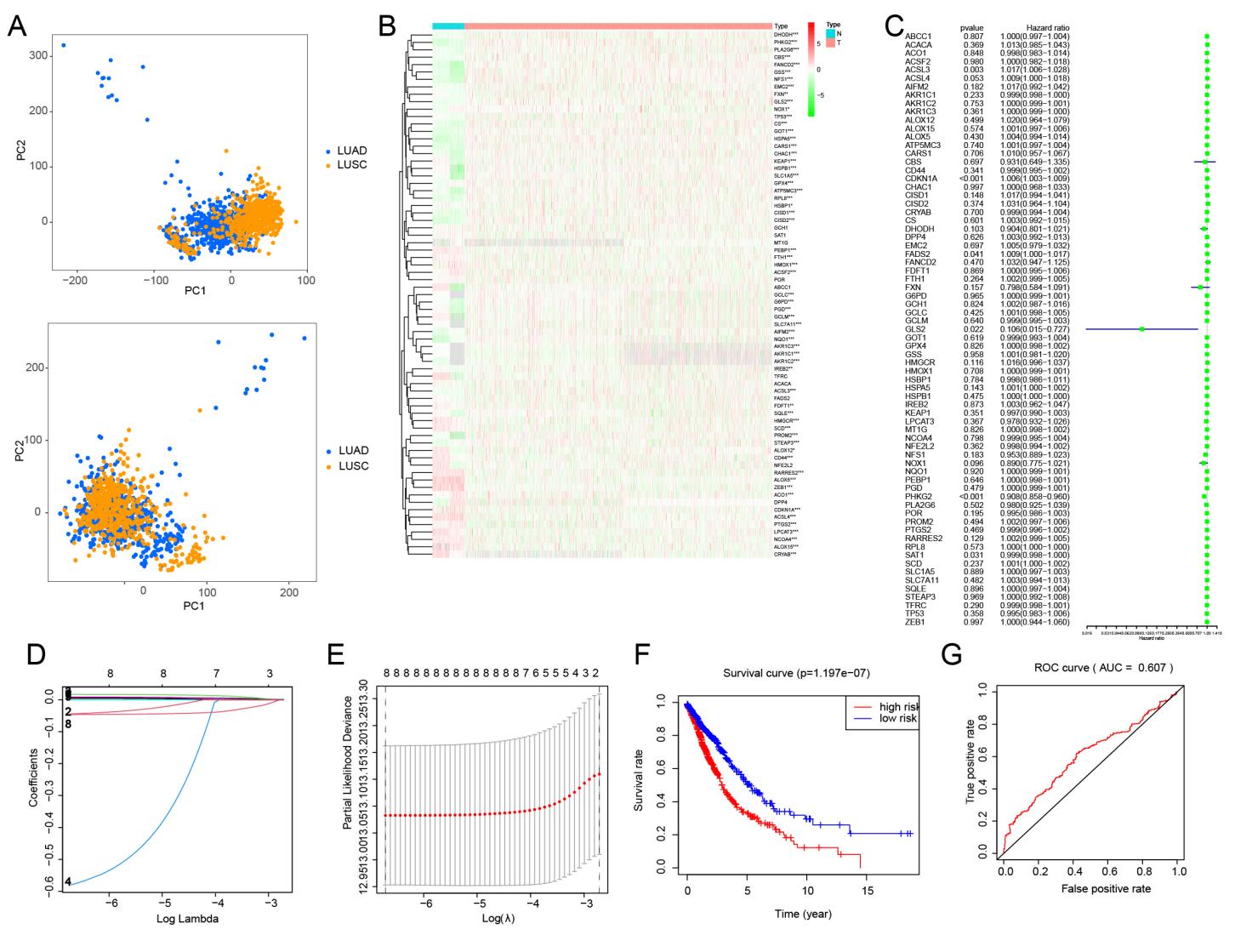
**

Supplementary Figure 2 TCGA NSCLC-based FAGs-Lasso risk regression model construction. A, Distribution of PCA before (top) and after (bottom) correction of TCGA NSCLC data (n_normal_ = 108, T_tumor_ = 1041). B, Expression heat map of TCGA NSCLC FAGs (N: normal; T: tumor) with color scale on the right (red represents up-regulated expression and green represents down-regulated expression). C, Forest plots showing univariate Cox analysis of 71 FAGs. D & E: TCGA NSCLC FAGs-Lasso regression λ and model regression coefficient plot (D) and cross-validation plot (E). F, survival analysis of patients in high-risk versus low-risk groups based on the FAGs-Lasso model. G, Risk score-based ROC curve for the FAGs-Lasso model.

**
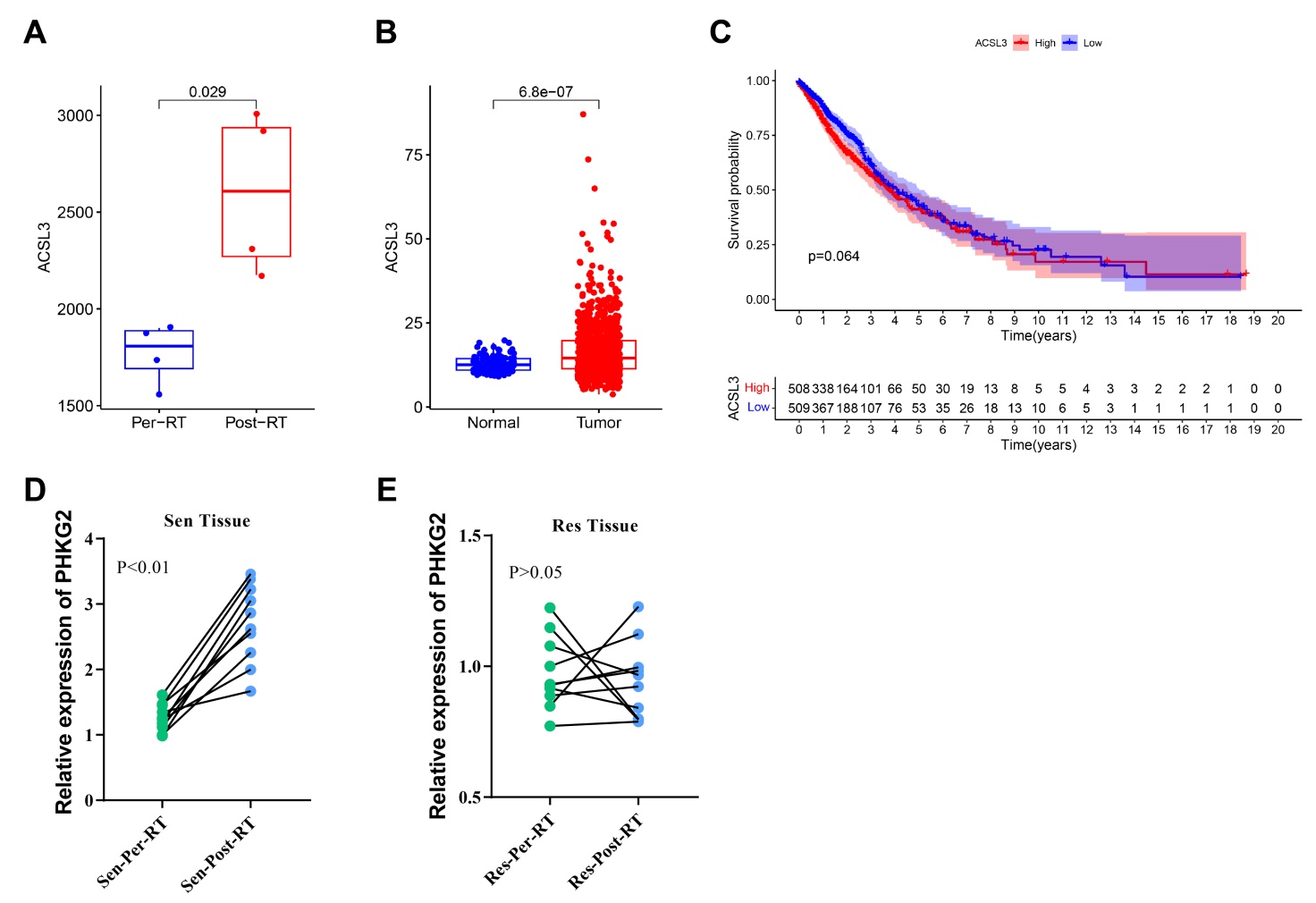
**

Supplementary Figure 3 Expression of ACSL3 and PHKG2 in NSCLC tissues of radiotherapy-sensitive and resistant patients. A, Expression of ACSL3 in RNAseq data. B, Expression of ACSL3 in TCGA-NSCLC data. C, Relationship curve between ACSL3 expression and patient survival in TCGA-NSCLC data. D & E: RT-qPCR detection of PHKG2 expression in NSCLC tissues (n = 10). Panel D compares sensitive PHKG2 expression changes before and after radiotherapy in tissues, and Panel E compares PHKG2 expression changes before and after radiotherapy in resistant tissues. ^ns^ *p* > 0.05, * *p* < 0.05, ** *p* < 0.01, *** *p* < 0.001.

**
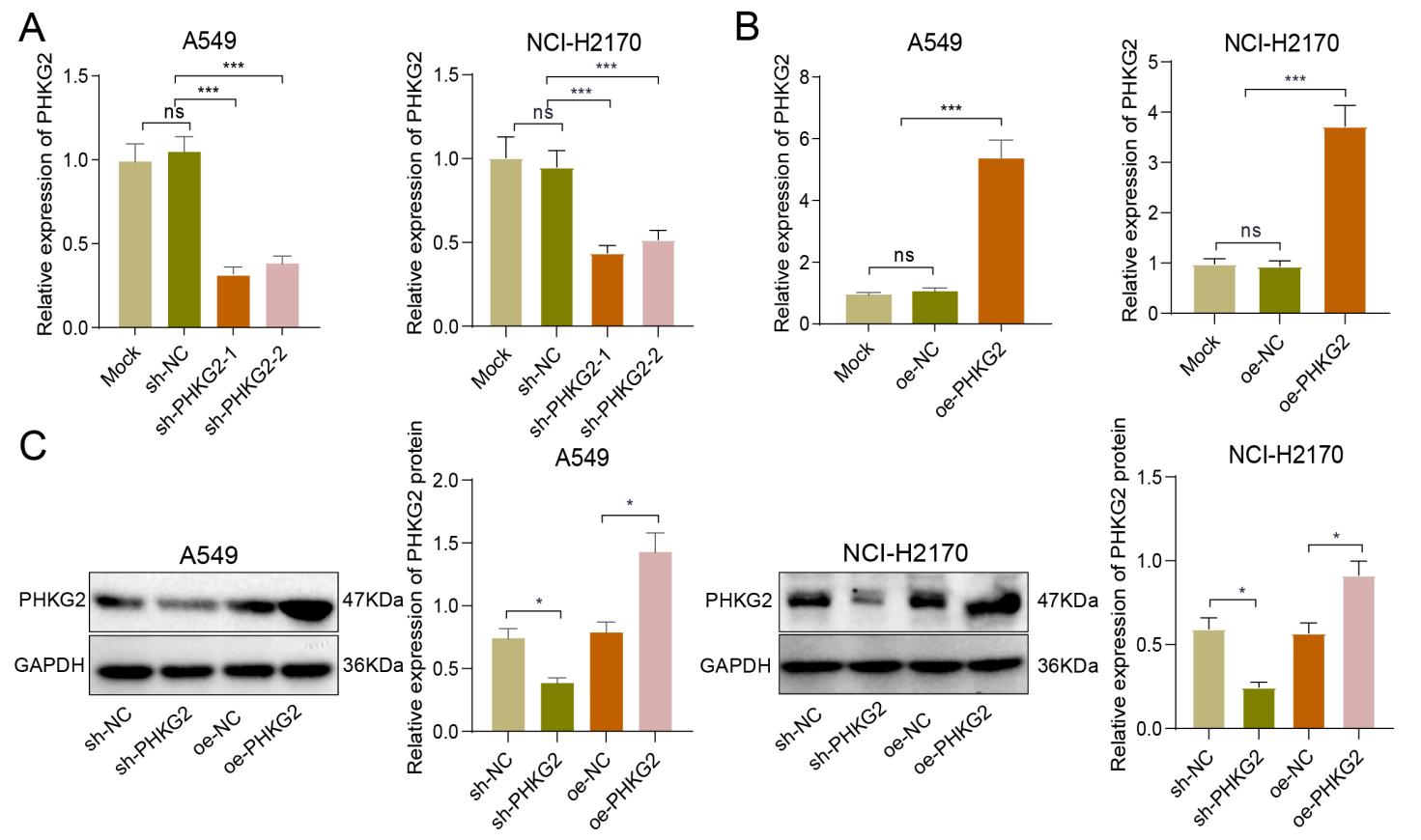
**

Supplementary Figure 4 Validation of PHKG2 knockdown and overexpression in A549 and NCI-H2170 cells. A-B, Validation of PHKG2 knockdown and overexpression by RT-qPCR. C, Verification of PHKG2 knockdown and overexpression by Western blot analysis. All cell experiments were independently repeated three times. ^ns^ *p* > 0.05, * *p* < 0.05, ** *p* < 0.01, *** *p* < 0.001.

**
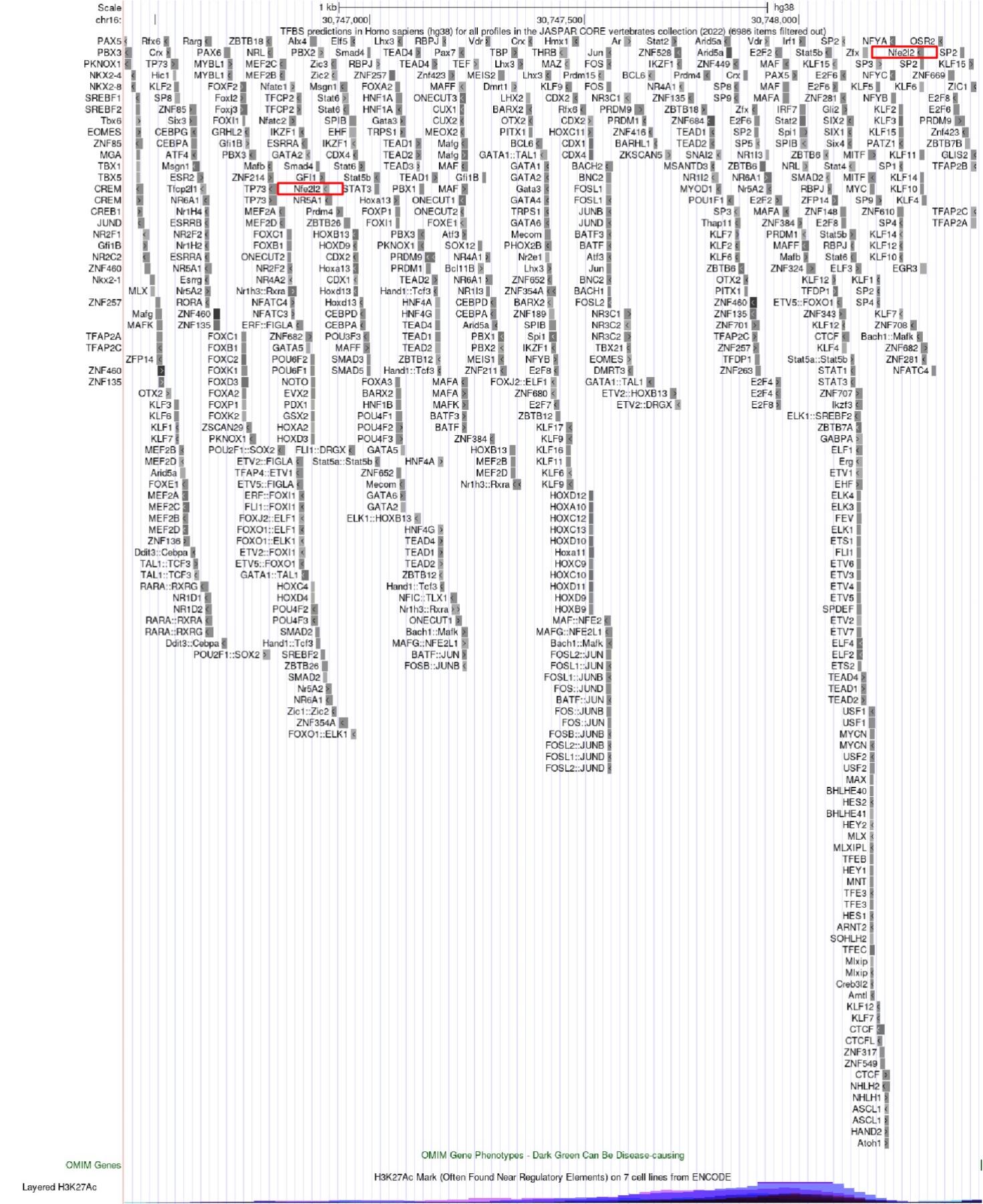
**

**Supplementary Figure 5** PHKG2 transcription factor predicted through UCSC (minimum score = 380) (The figure was predicted using the UCSC website (https://genome.ucsc.edu/) ).

**
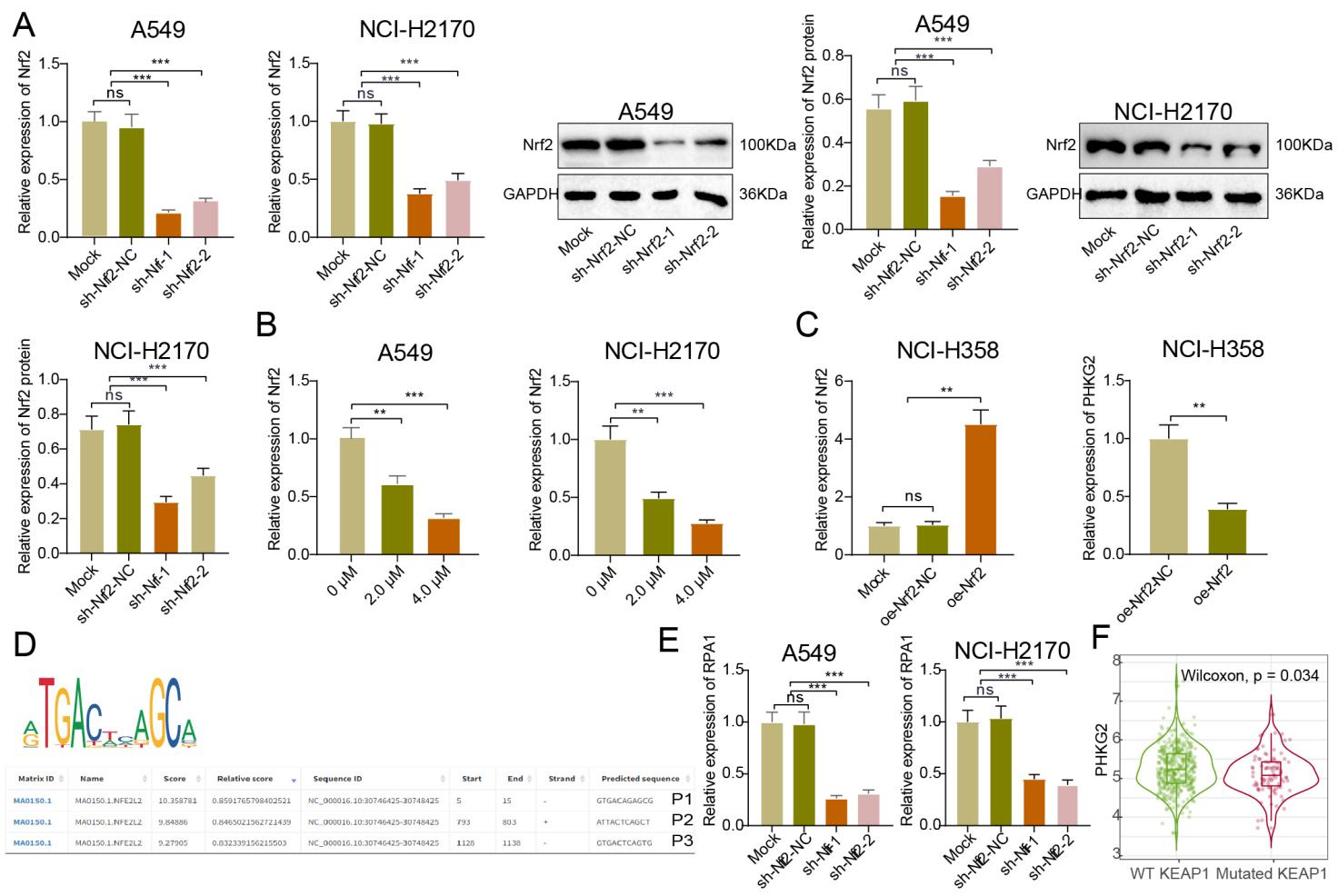
**

Supplementary Figure 6 Validation of gene expression knockdown and prediction of potential binding sites of NRF2 to the promoter region of PHKG2. A, RT-qPCR and Western blot to verify NRF2 knockdown effect in A549 cell line. B, RT-qPCR to verify the effect of ML385 on the inhibition of NRF2 expression in the A549 cell line. C, RT-qPCR to detect NRF2 overexpression in NCI-H358 cell line and its effect on PHKG2 expression. D, The NRF2 binding sequence identity map obtained from the JASPAR website and its predicted binding site to the PHKG2 promoter region. E, RT-qPCR to verify RPA1 knockdown effect in the A549 and NCI-2170 cell lines. F, The expression levels of PHKG2 in patients with wild-type and mutant KEAP1 in TCGA-LUAD data. All cell experiments were independently repeated three times. ^ns^ *p* > 0.05, * *p* < 0.05, ** *p* < 0.01, *** *p* < 0.001.

**
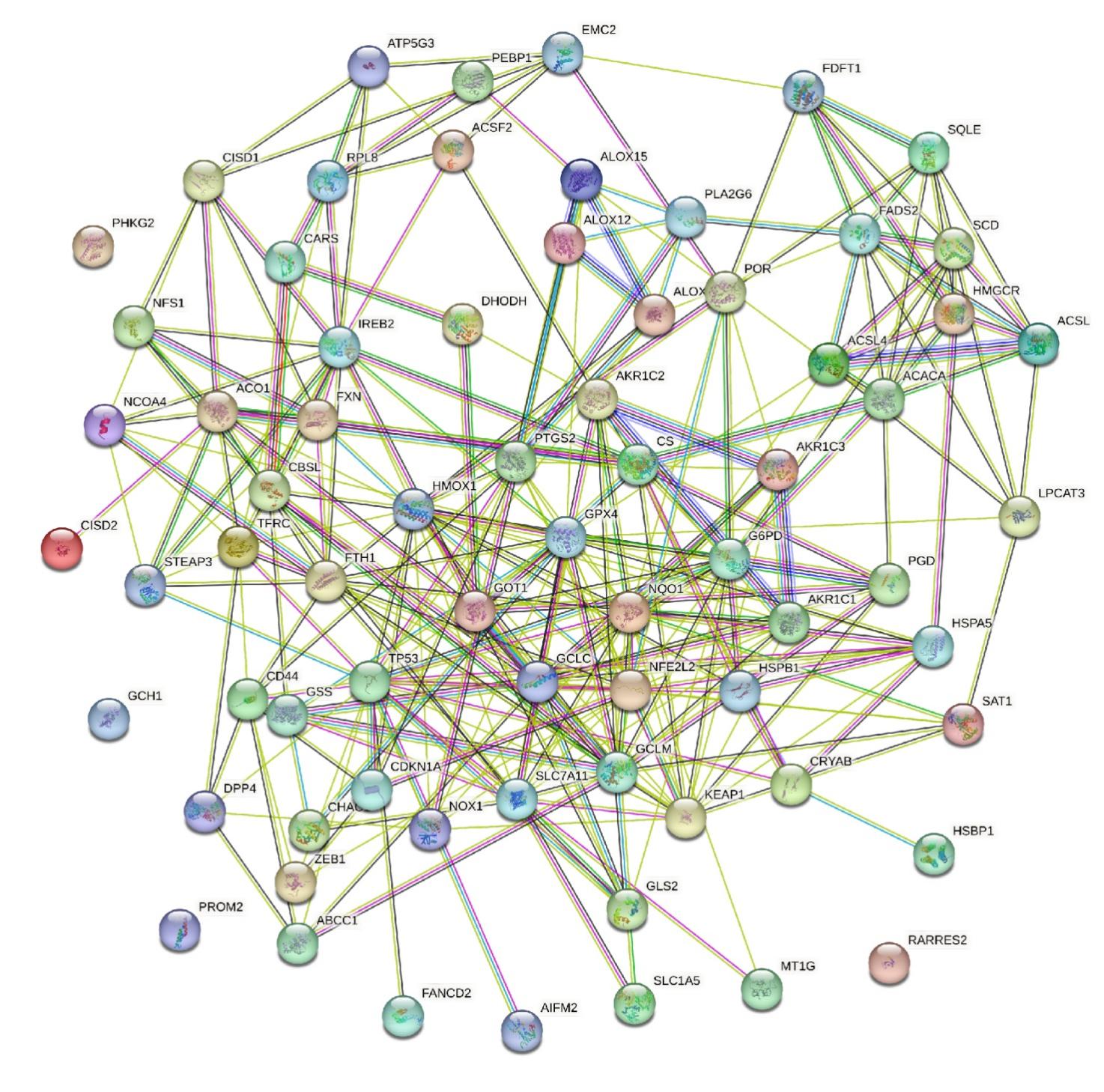
**

**Supplementary Figure 7** PPI network of 71 FAGs (The figure was generated from data input into the String website (https://creativecommons.org/licenses/by/4.0/)).

**Original WB images**


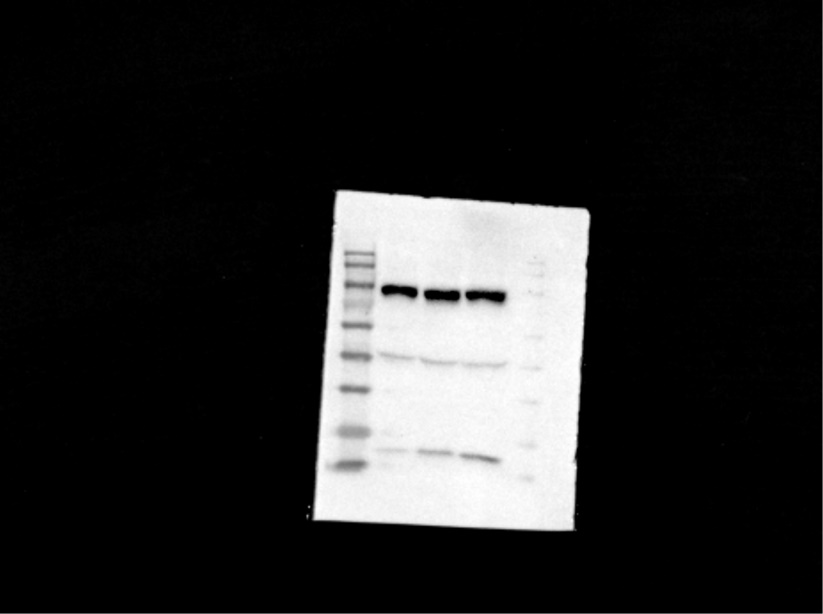


Supplementary Figure 8 (Figure 3B-1-1)


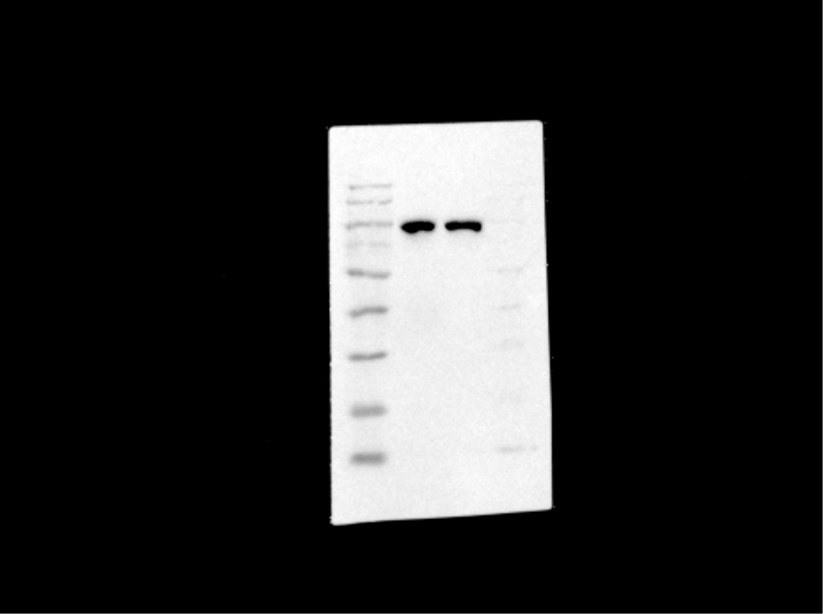


Supplementary Figure 9(Figure 3B-1-2)


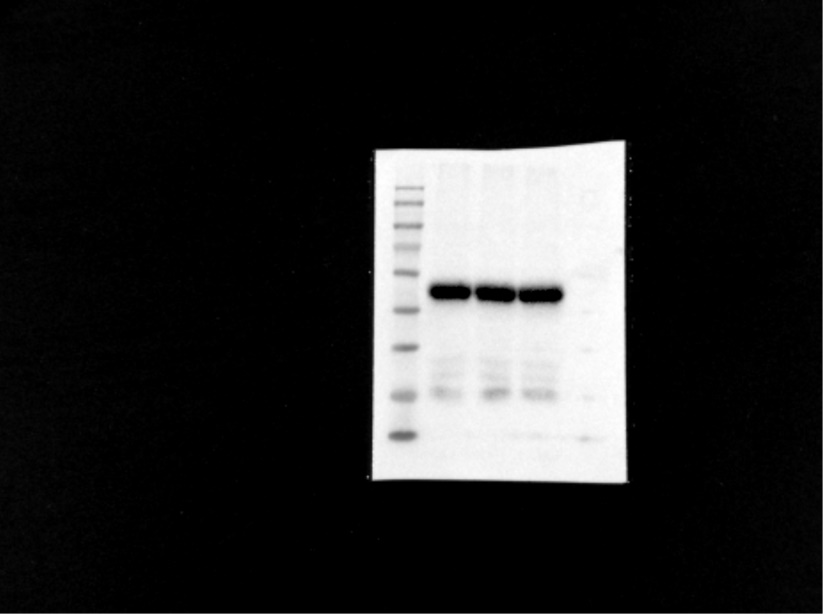


Supplementary Figure 10(Figure 3B-2-1)


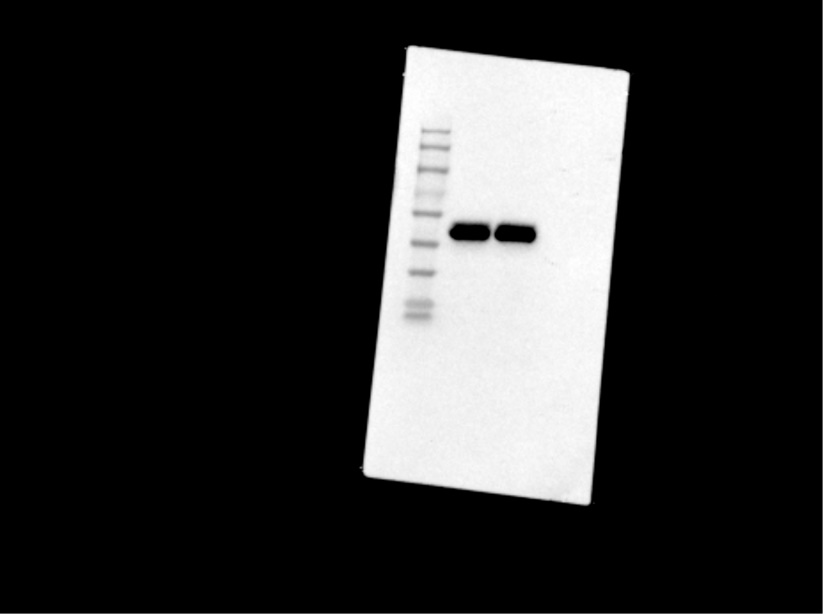


Supplementary Figure 11 (Figure 3B-2-2)


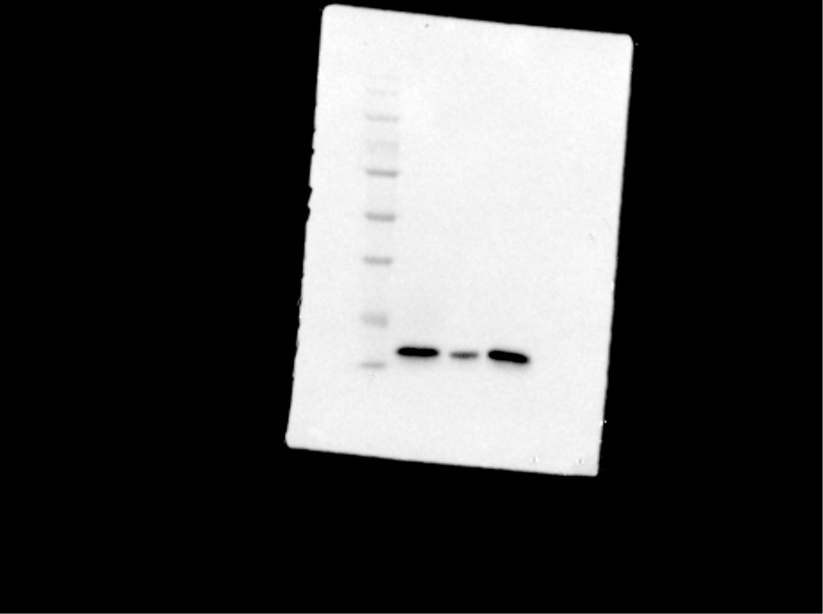


Supplementary Figure 12(Figure 3B-3-1)


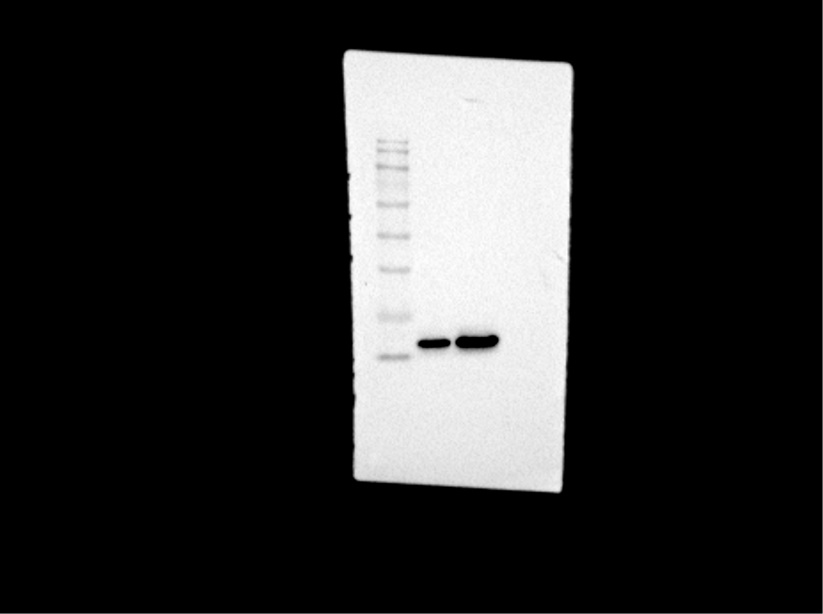


Supplementary Figure 13(Figure 3B-3-2)


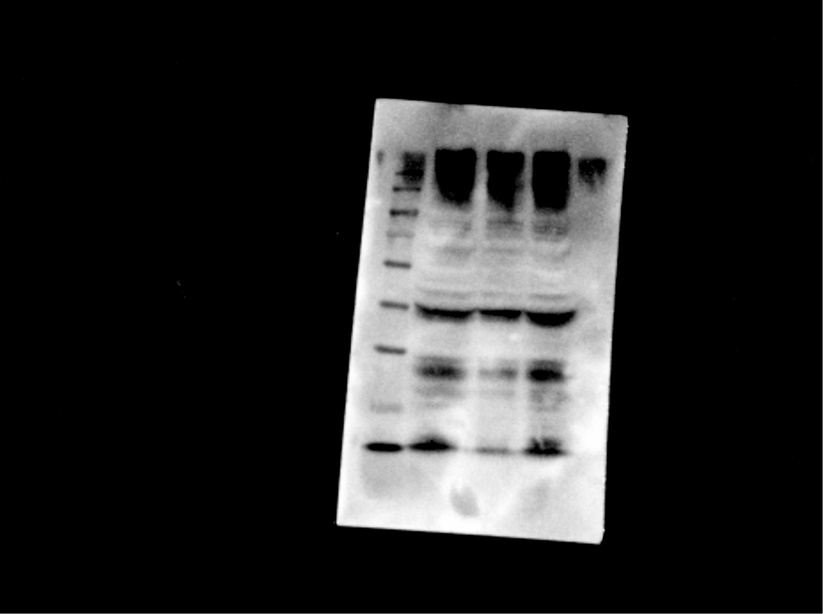


Supplementary Figure 14(Figure 3B-4-1)


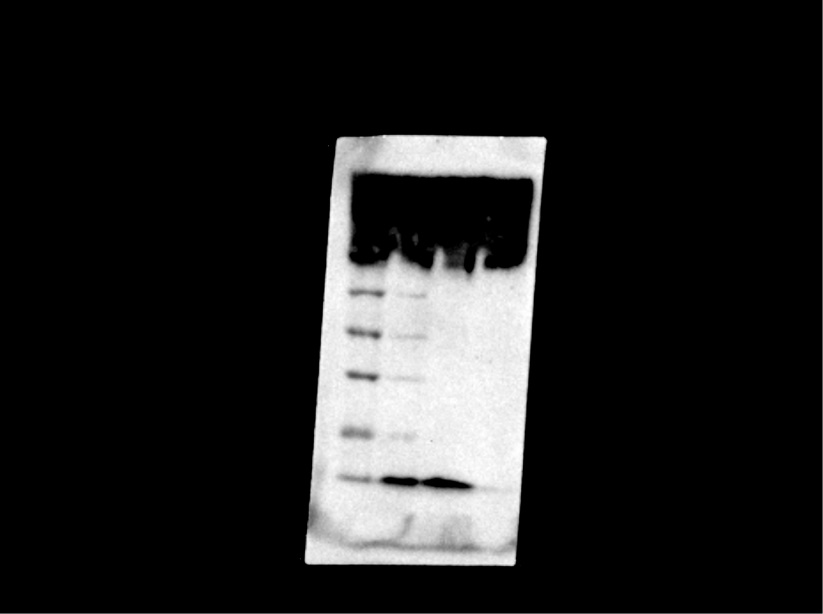


Supplementary Figure 15(Figure 3B-4-2)


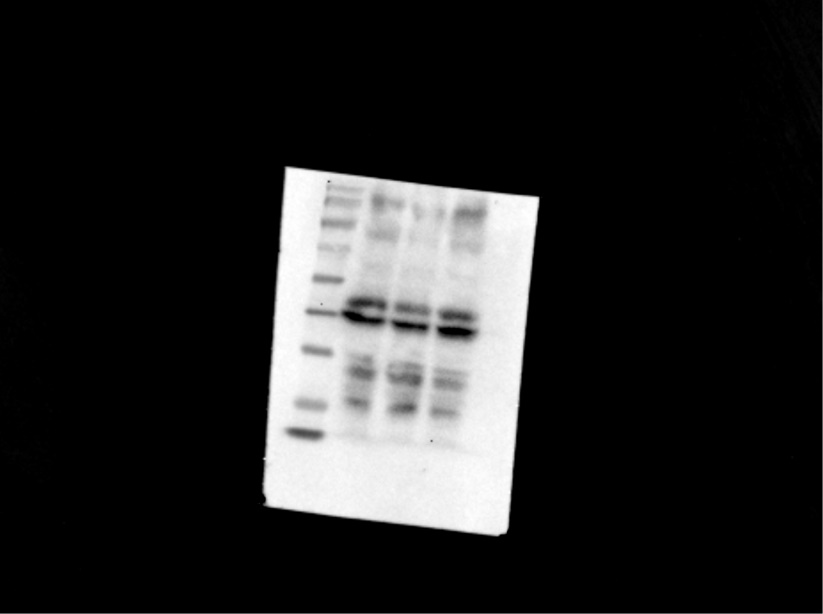


Supplementary Figure 16(Figure 3B-5-1)


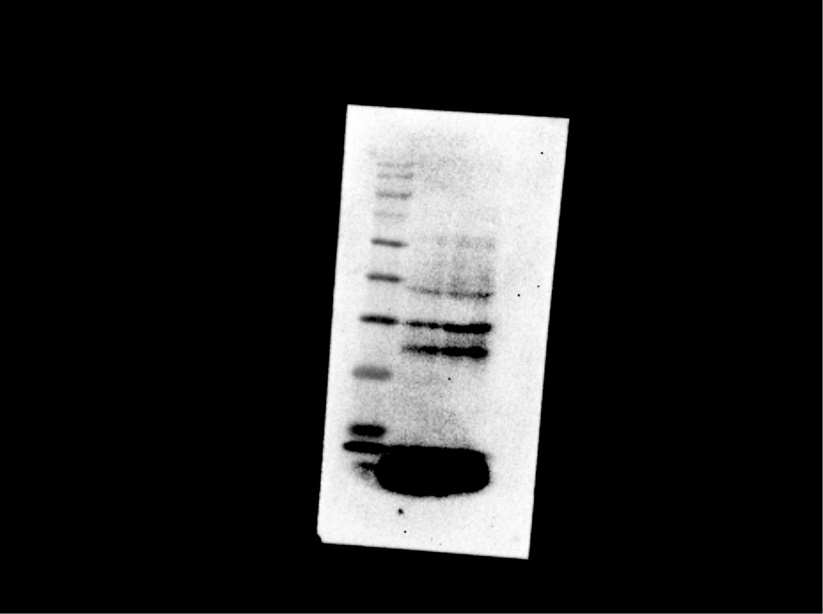


Supplementary Figure 17(Figure 3B-5-2)


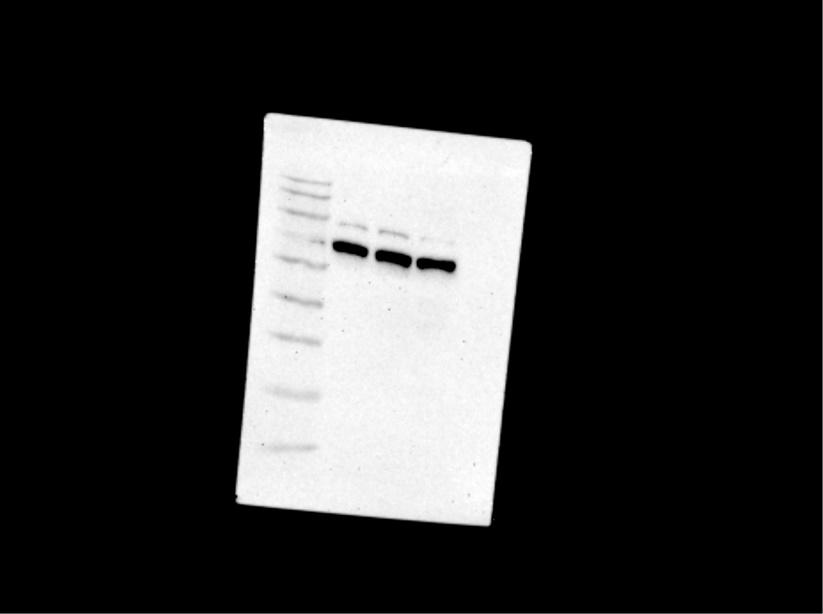


Supplementary Figure 18(Figure 3B-6-1)


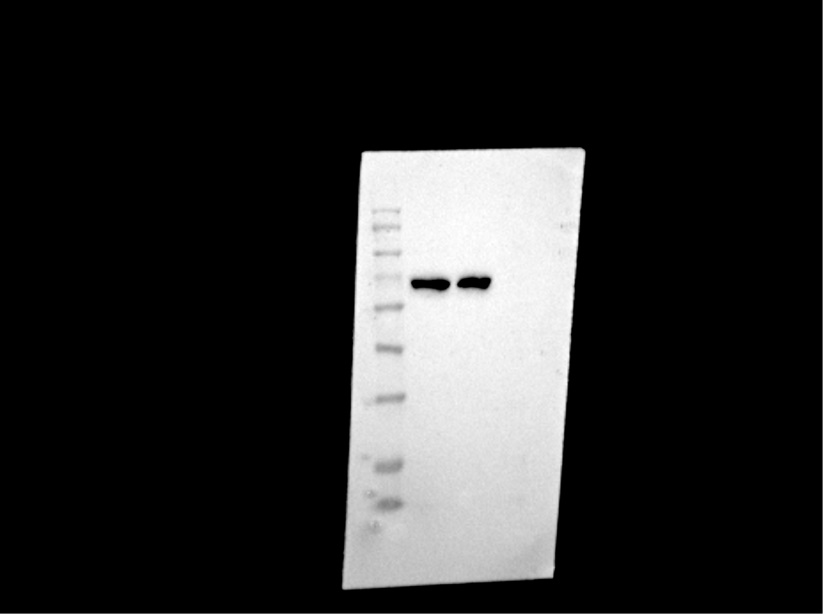


Supplementary Figure 19(Figure 3B-6-2)


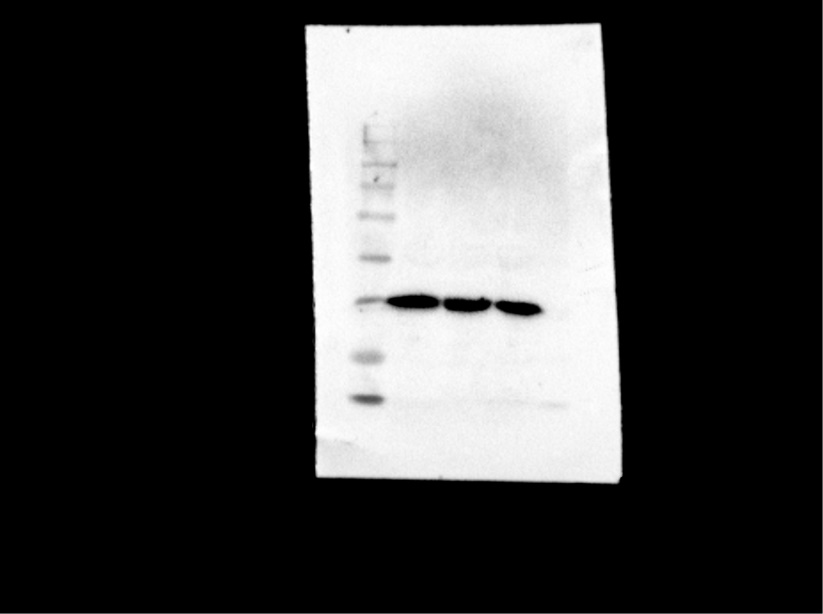


Supplementary Figure 20(Figure 3B-7-1)


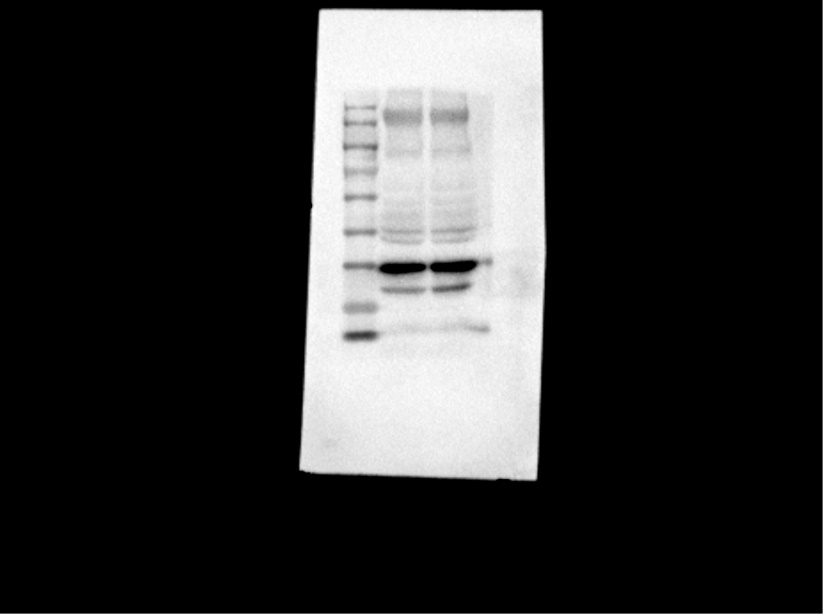


Supplementary Figure 21(Figure 3B-7-2)


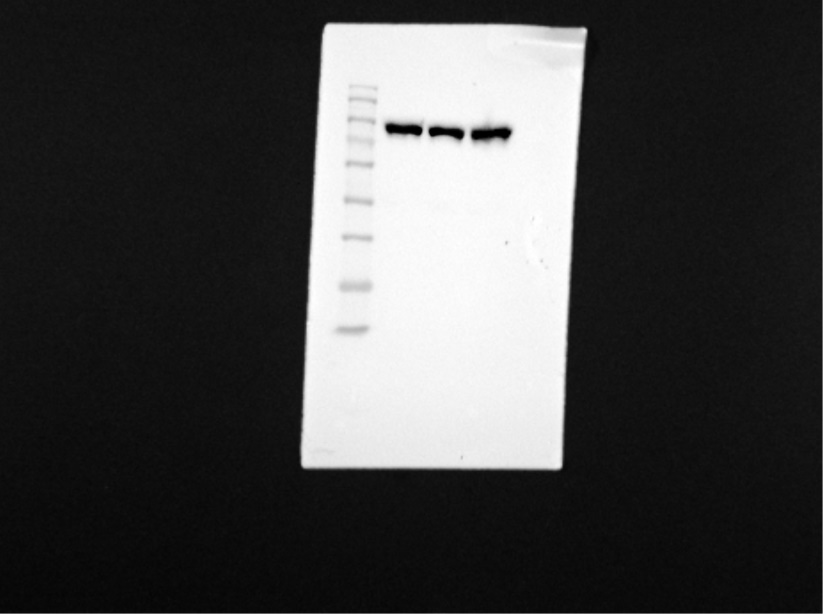


Supplementary Figure 22(Figure 3B-8-1)


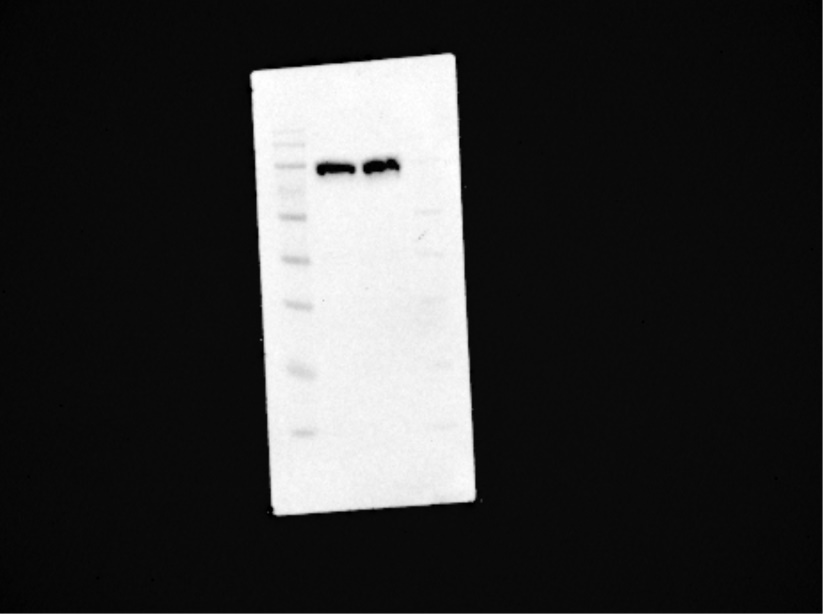


Supplementary Figure 23(Figure 3B-8-2)


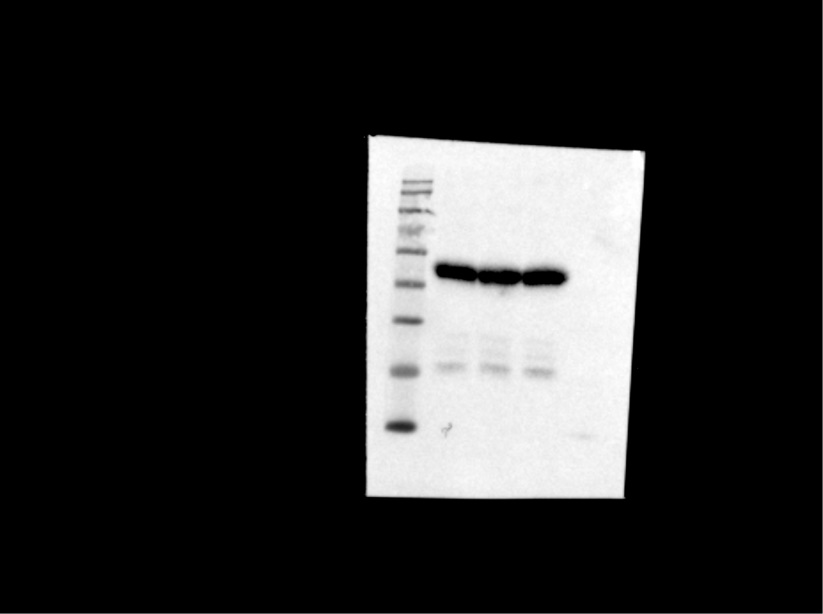


Supplementary Figure 24(Figure 3B-9-1)


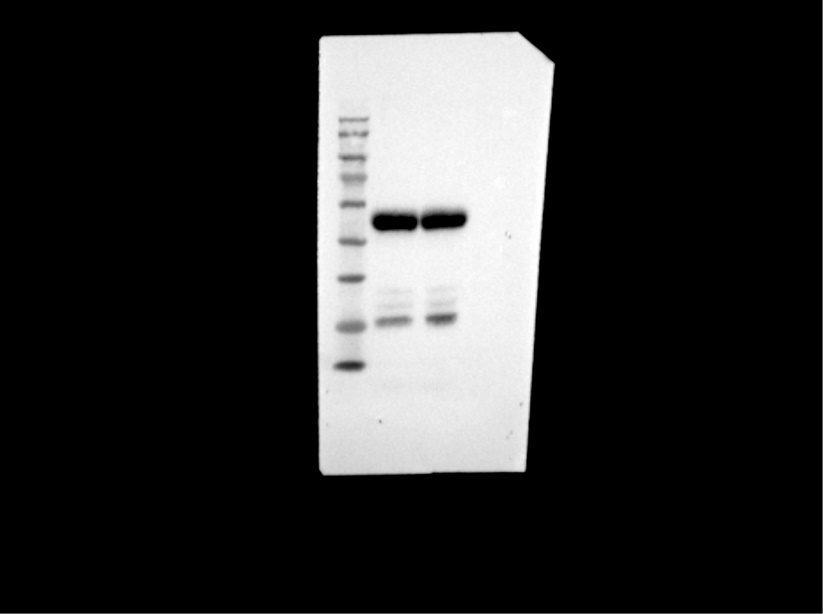


Supplementary Figure 25(Figure 3B-9-2)


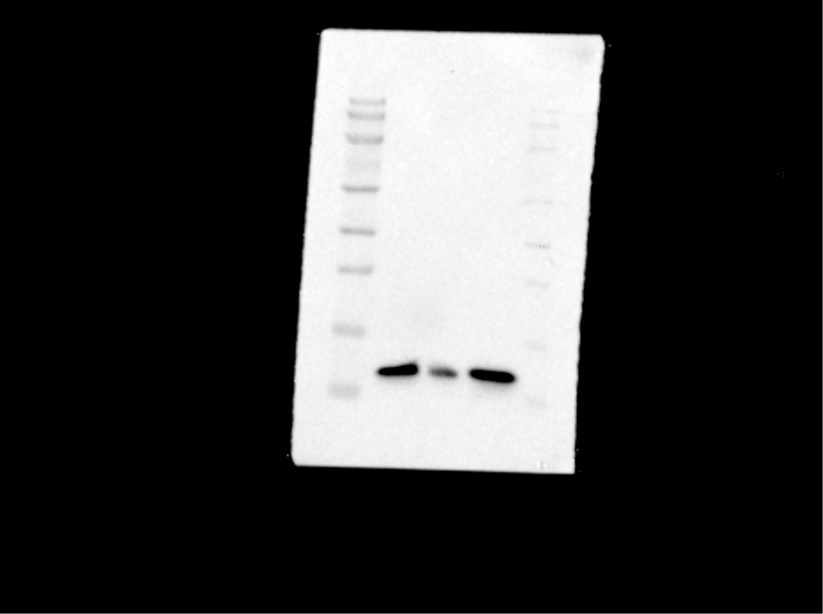


Supplementary Figure 26(Figure 3B-10-1)


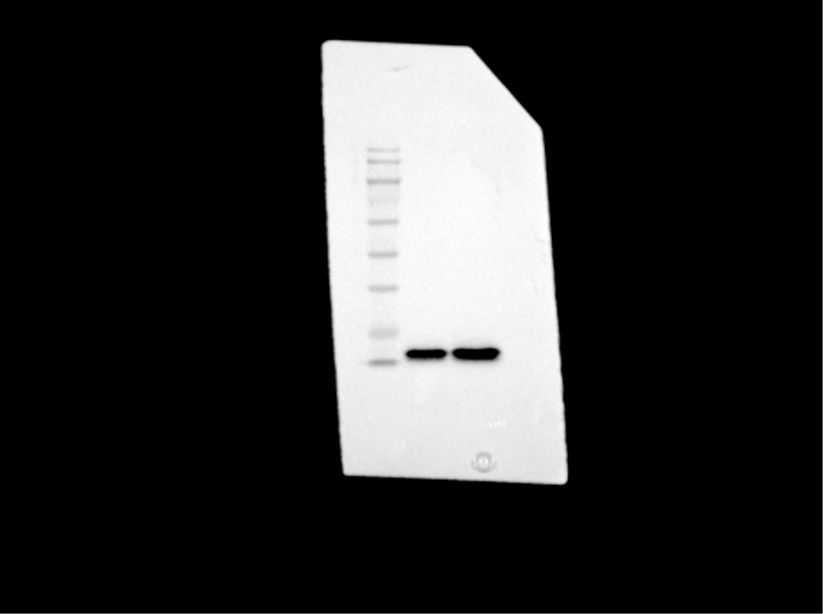


Supplementary Figure 27(Figure 3B-10-2)


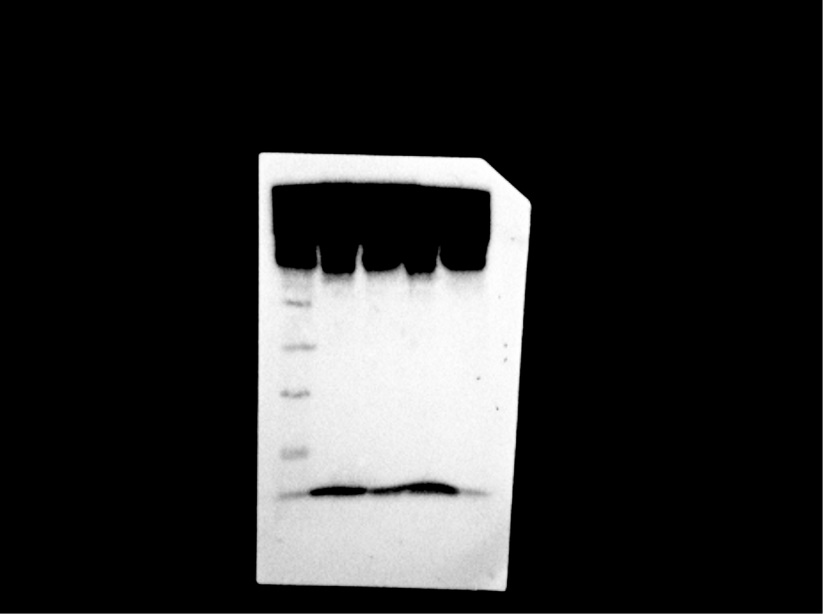


Supplementary Figure 28(Figure 3B-11-1)


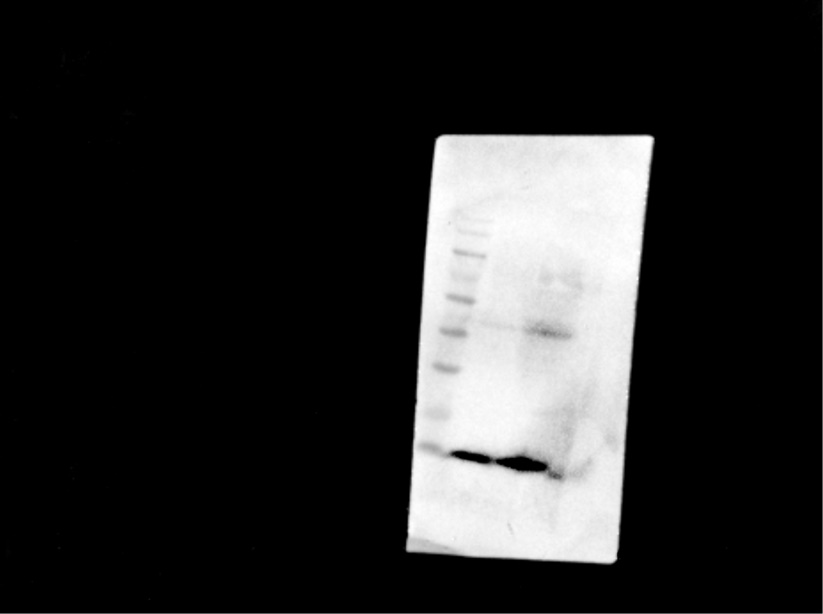


Supplementary Figure 29(Figure 3B-11-2)


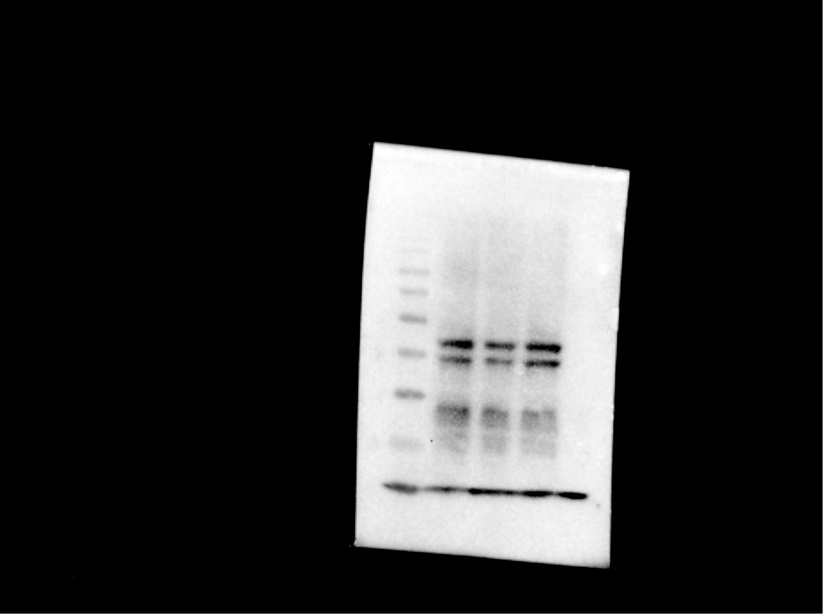


Supplementary Figure 30(Figure 3B-12-1)


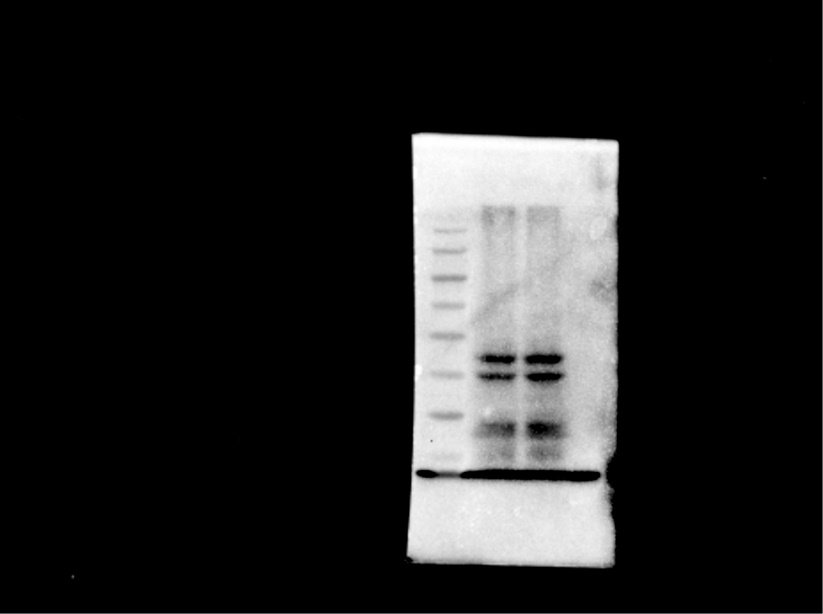


Supplementary Figure 31(Figure 3B-12-2)


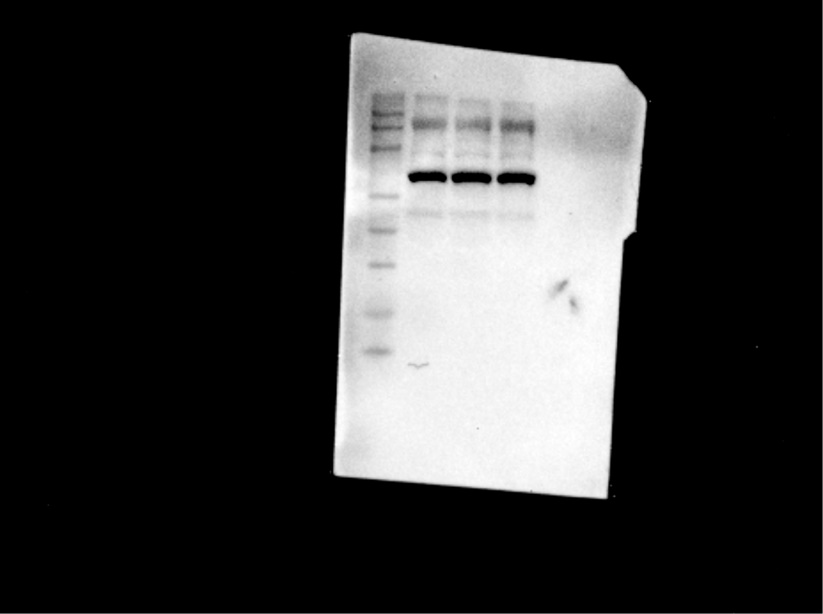


Supplementary Figure 32(Figure 3B-13-1)


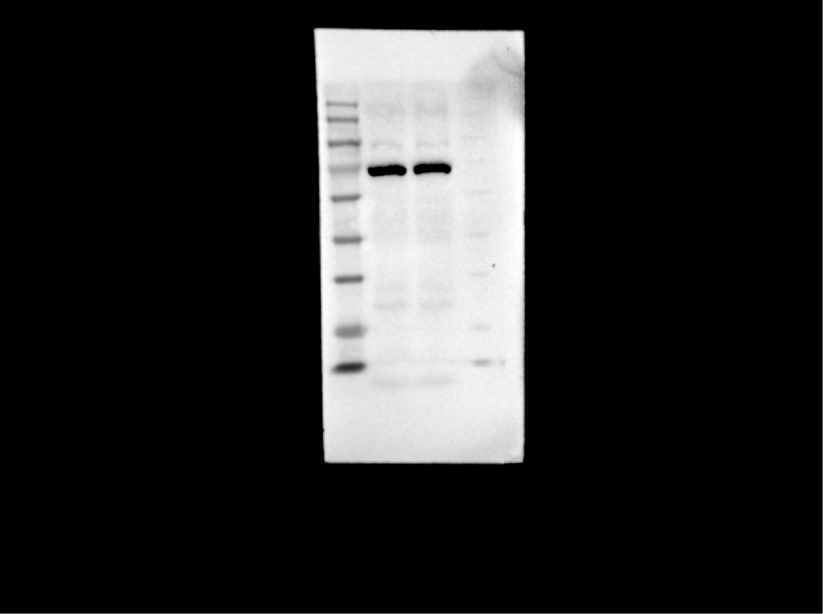


Supplementary Figure 33(Figure 3B-13-2)


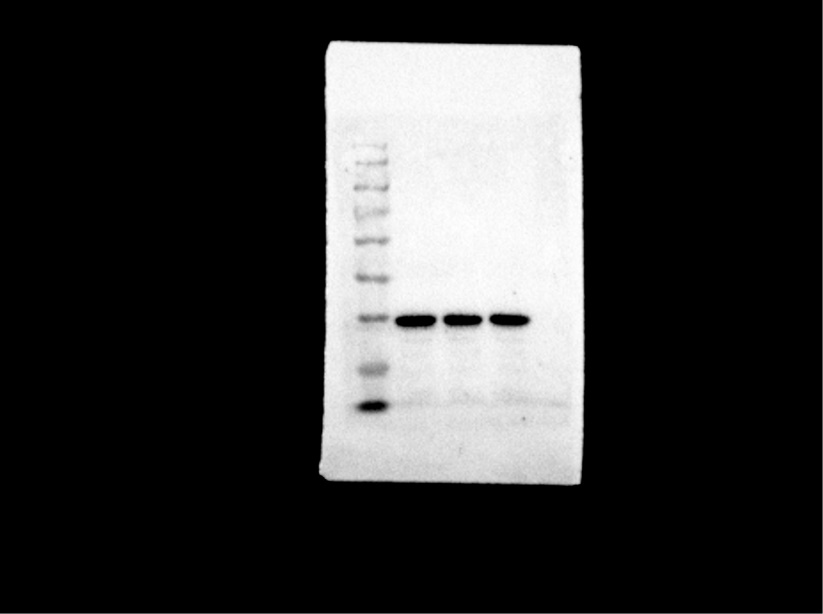


Supplementary Figure 34(Figure 3B-14-1)


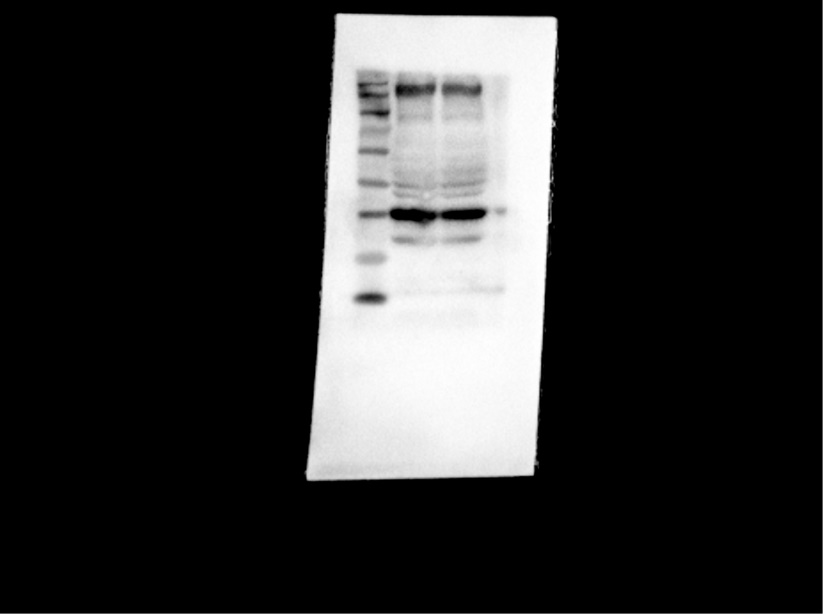


Supplementary Figure 35(Figure 3B-14-2)


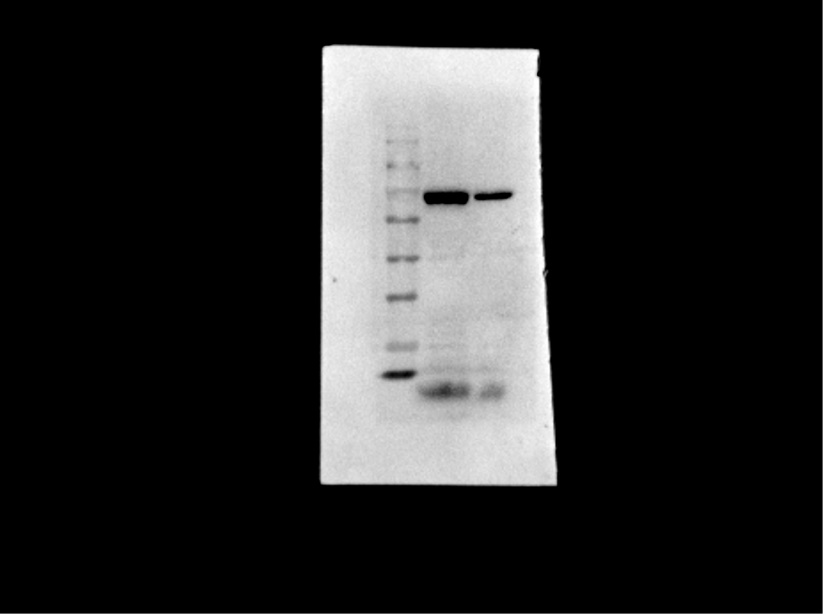


Supplementary Figure 36(Figure 3C-1)


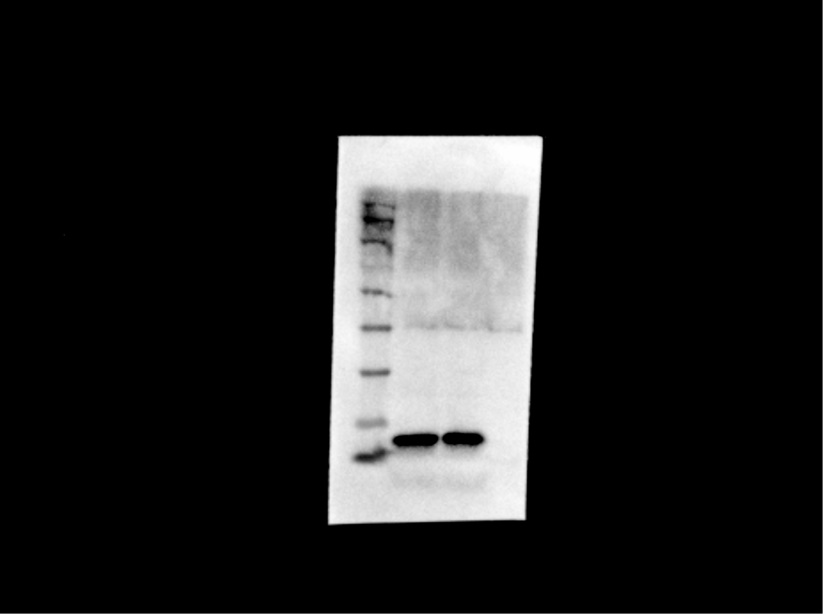


Supplementary Figure 37(Figure 3C-2)


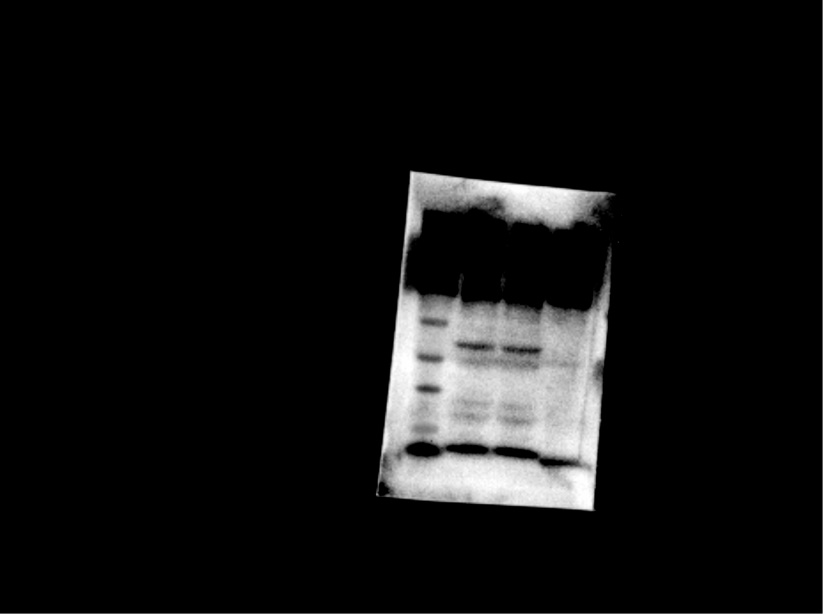


Supplementary Figure 38(Figure 3C-3)


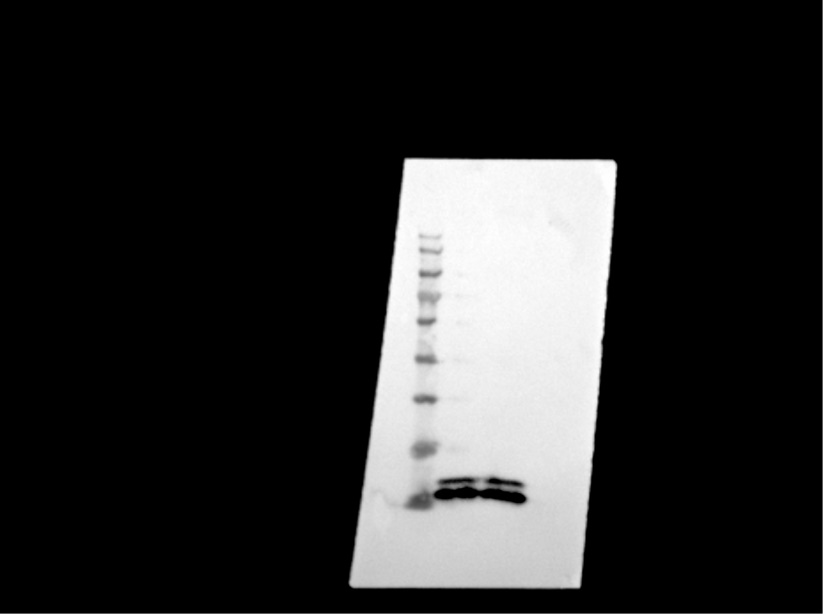


Supplementary Figure 39(Figure 3C-4)


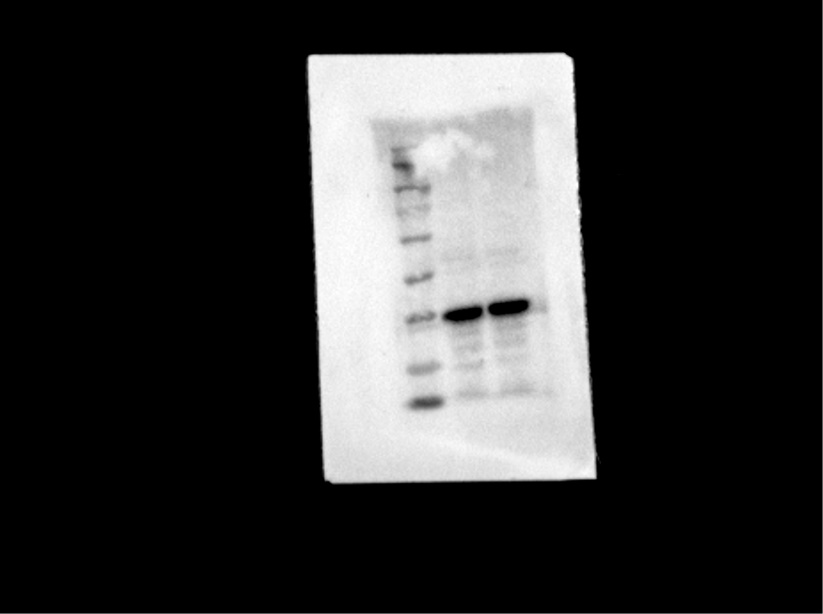


Supplementary Figure 40(Figure 3C-5)


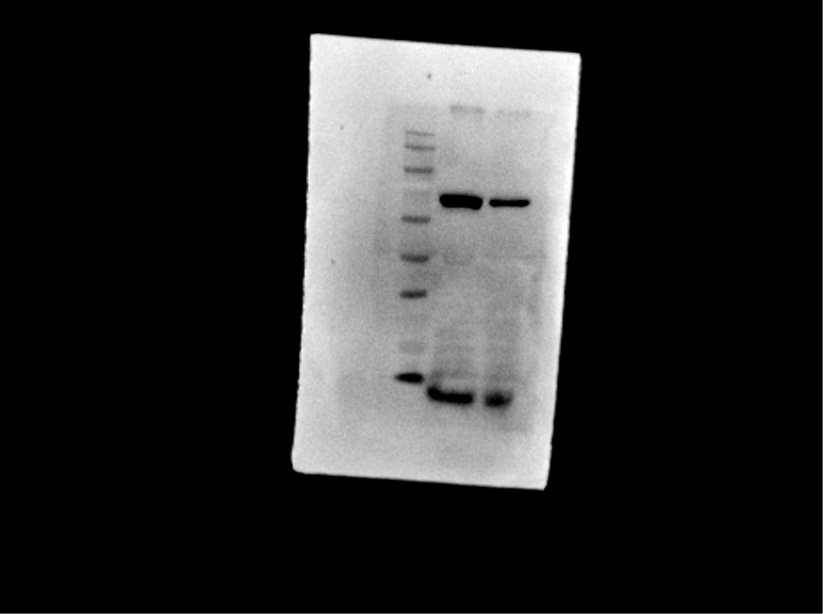


Supplementary Figure 41(Figure 3C-6)


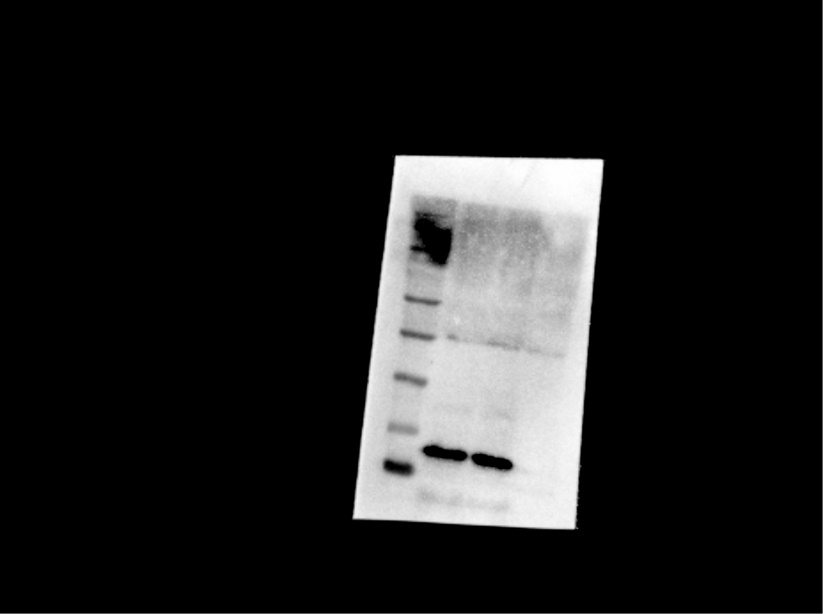


Supplementary Figure 42(Figure 3C-7)


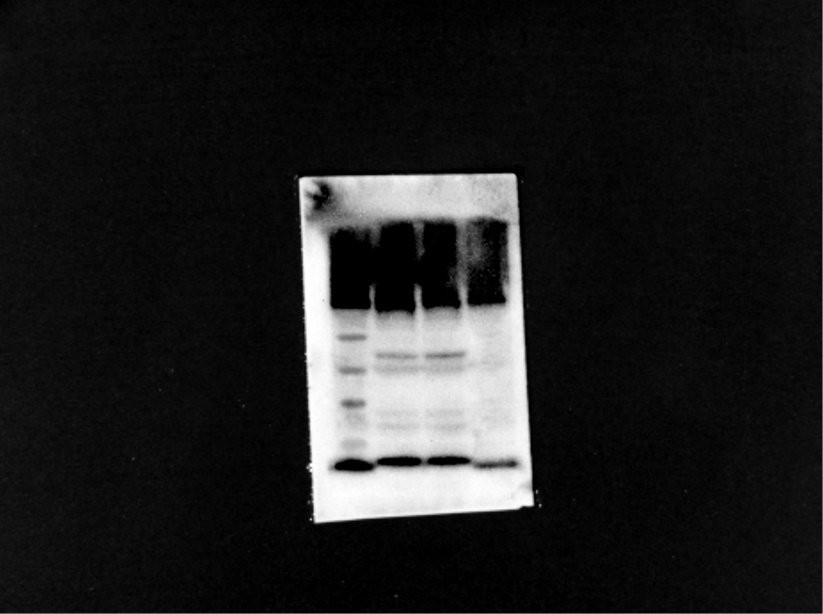


Supplementary Figure 43(Figure 3C-8)


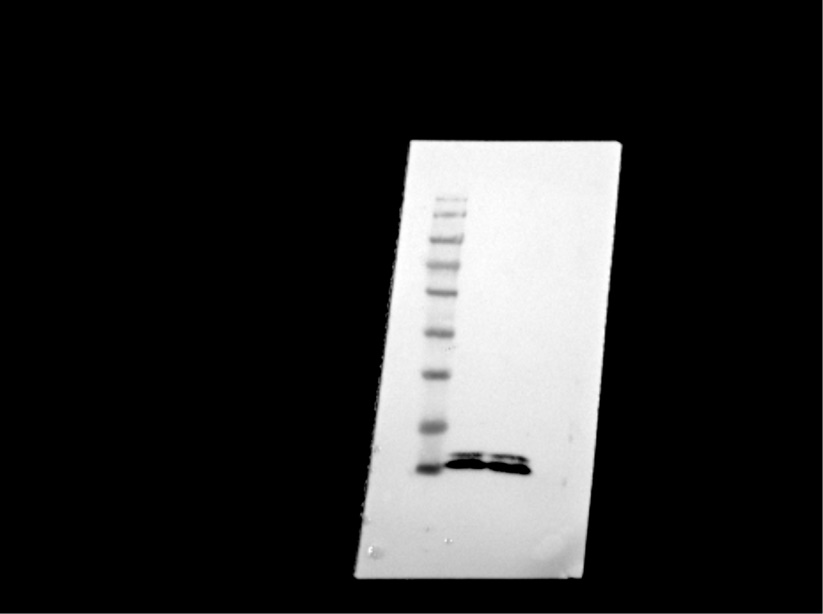


Supplementary Figure 44(Figure 3C-9)


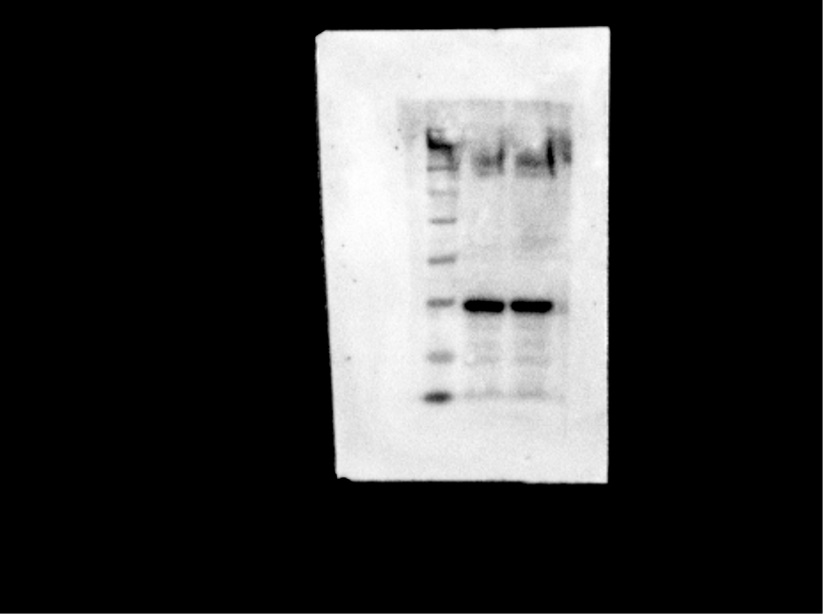


Supplementary Figure 45(Figure 3C-10)


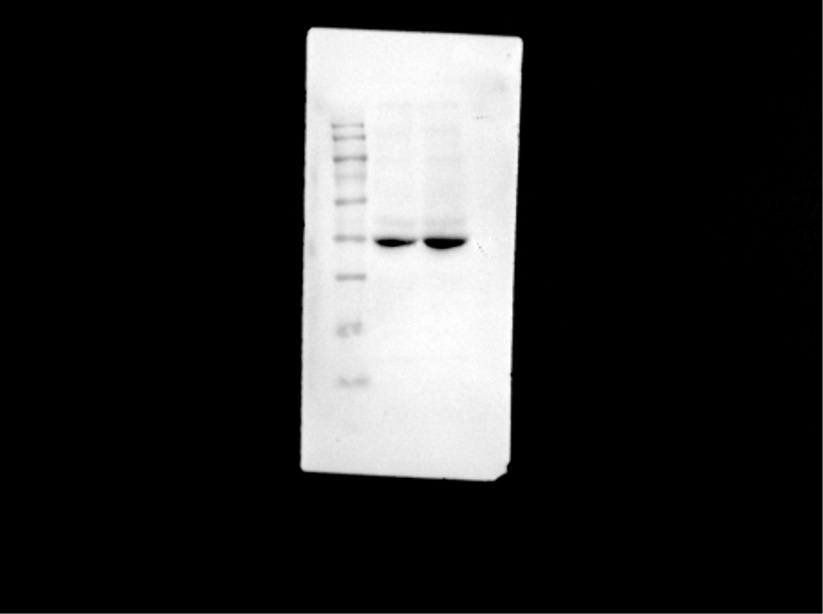


Supplementary Figure 46(Figure 5B-1)


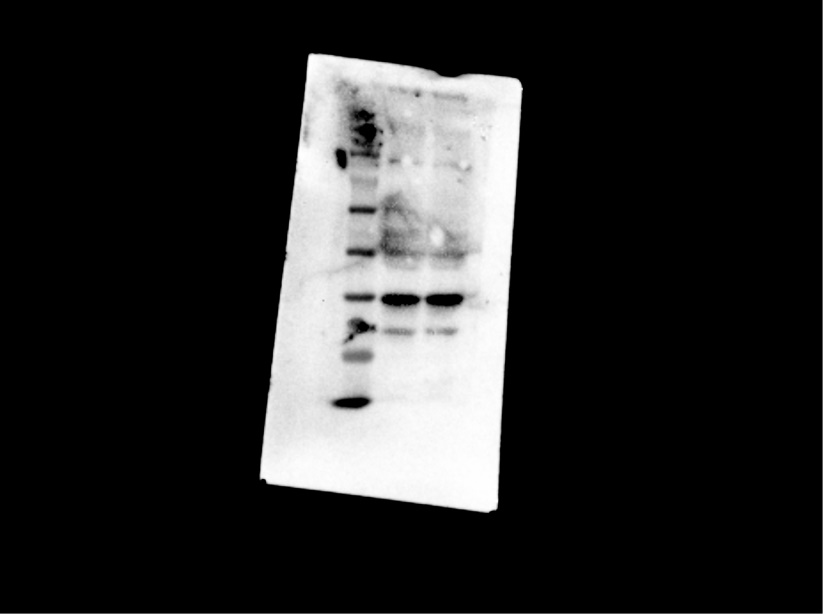


Supplementary Figure 47(Figure 5B-2)


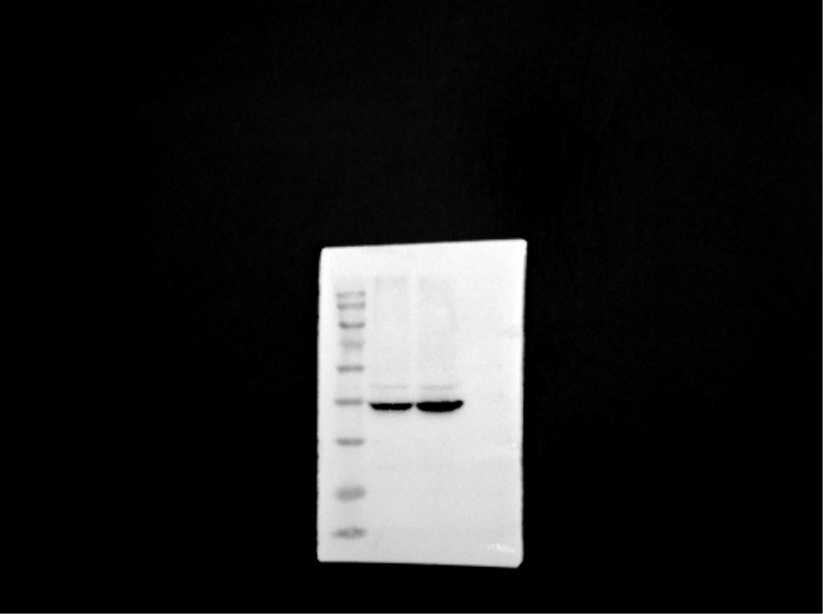


Supplementary Figure 48(Figure 5B-3)


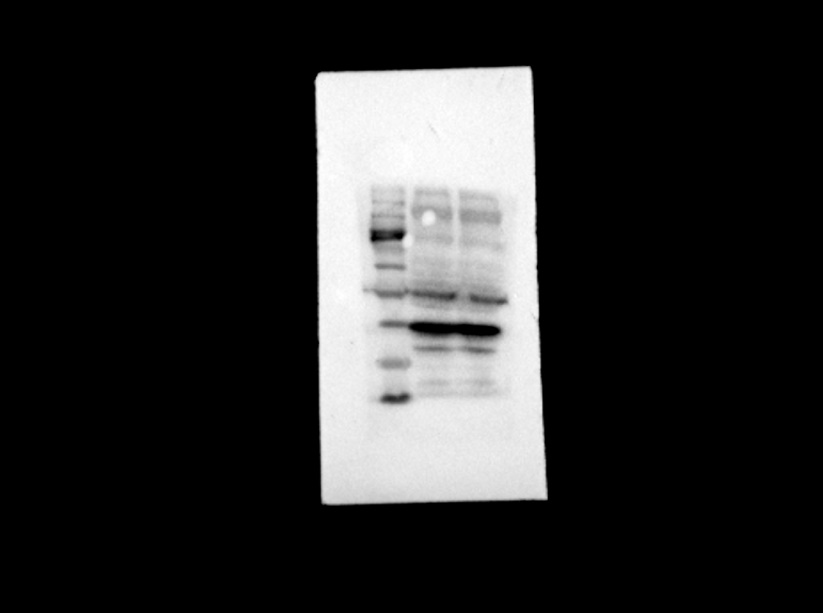


Supplementary Figure 49(Figure 5B-4)


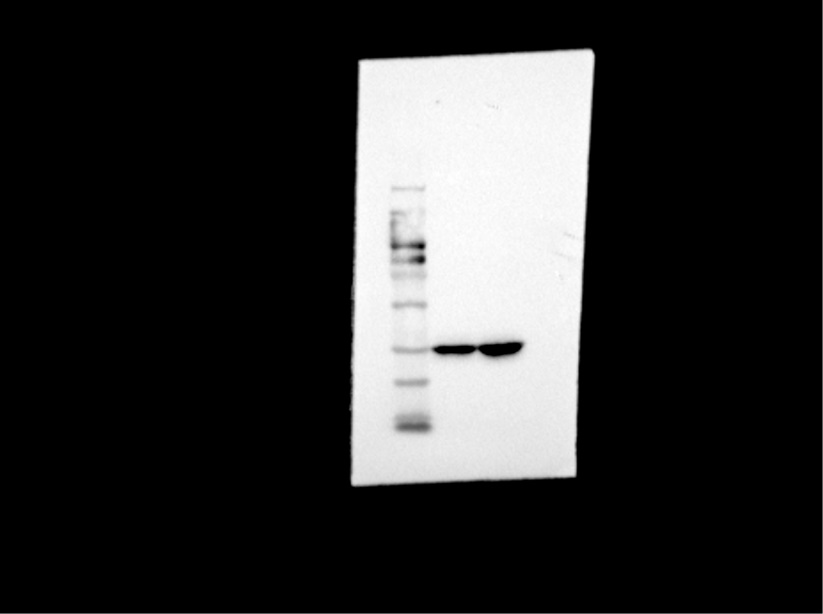


Supplementary Figure 50(Figure 5B-5)


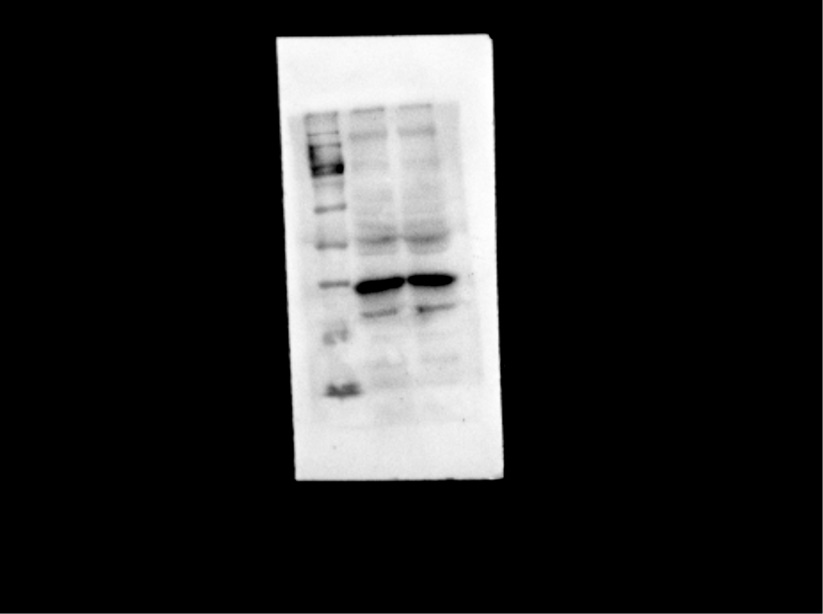


Supplementary Figure 51(Figure 5B-6)


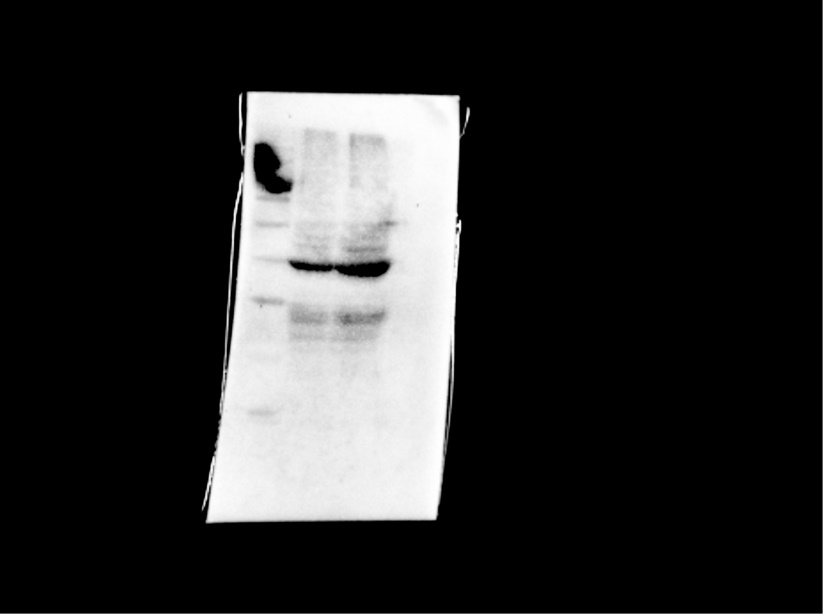


Supplementary Figure 52(Figure 5B-7)


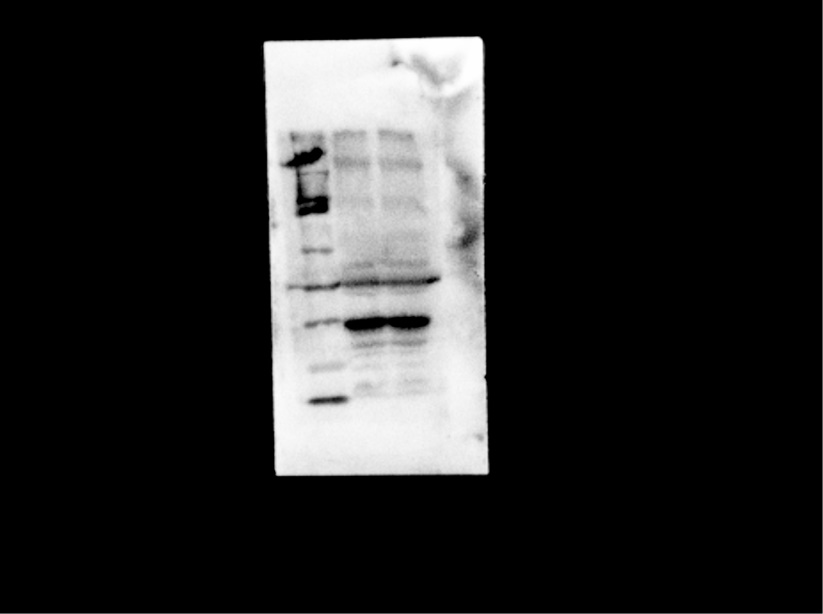


Supplementary Figure 53(Figure 5B-8)


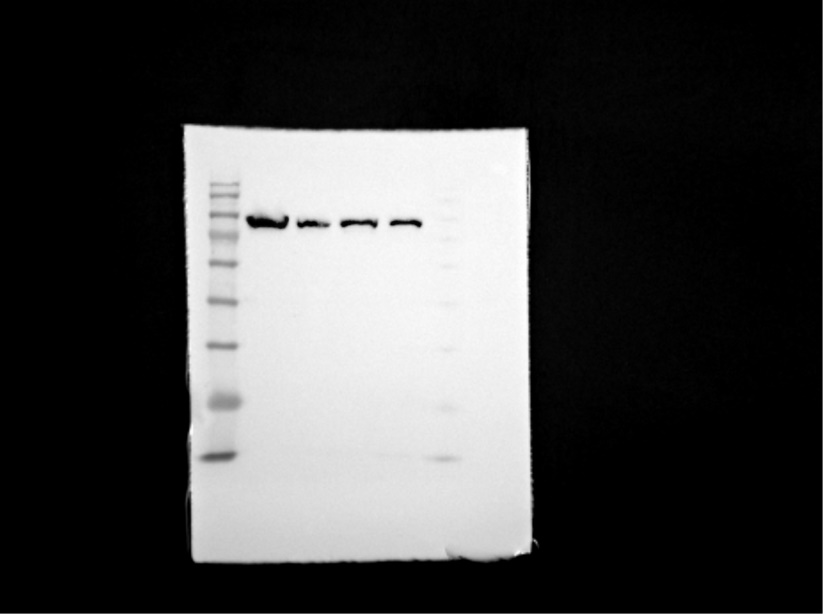


Supplementary Figure 54(Figure 5E-1)


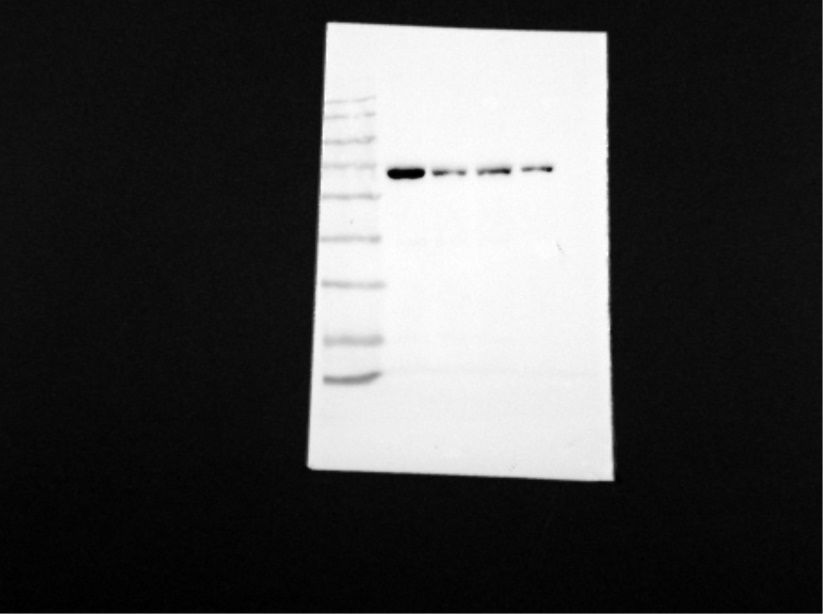


Supplementary Figure 55(Figure 5E-2)


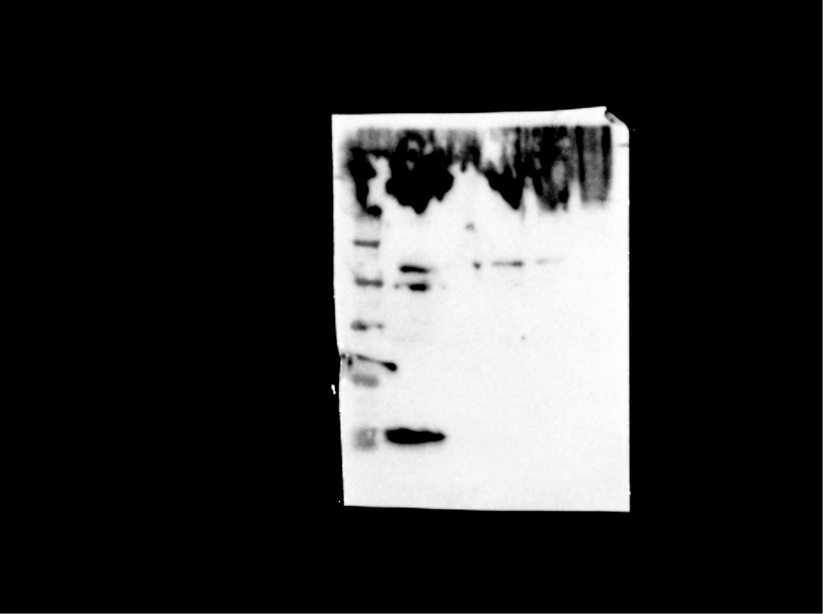


Supplementary Figure 56(Figure 5E-3)


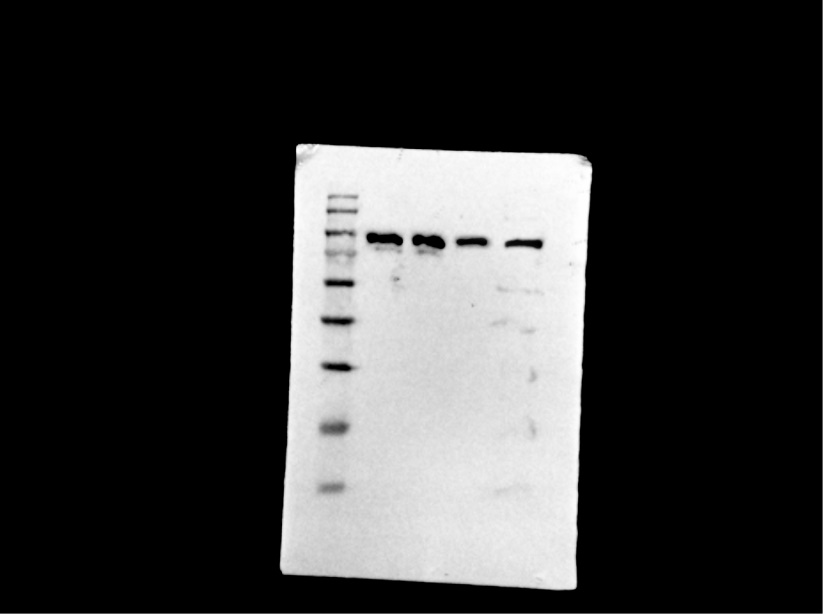


Supplementary Figure 57(Figure 5E-4)


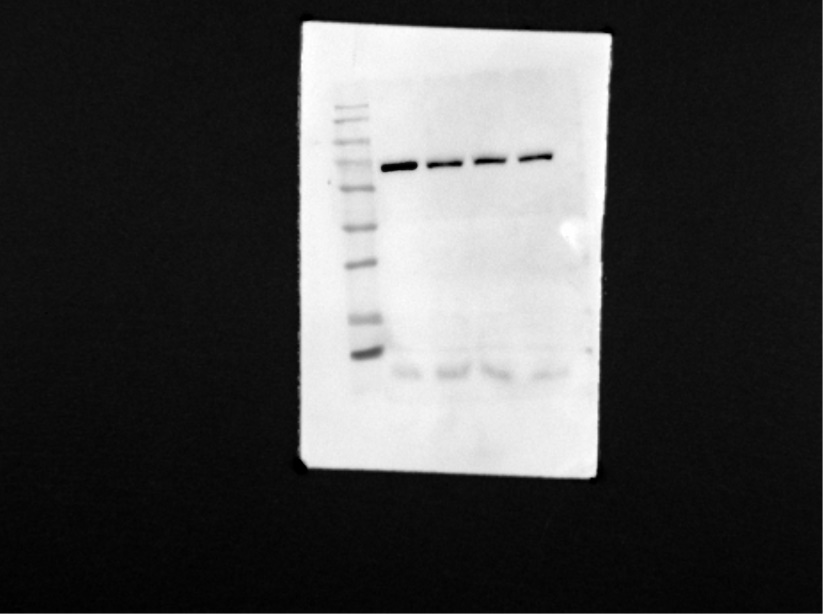


Supplementary Figure 58(Figure 5E-5)


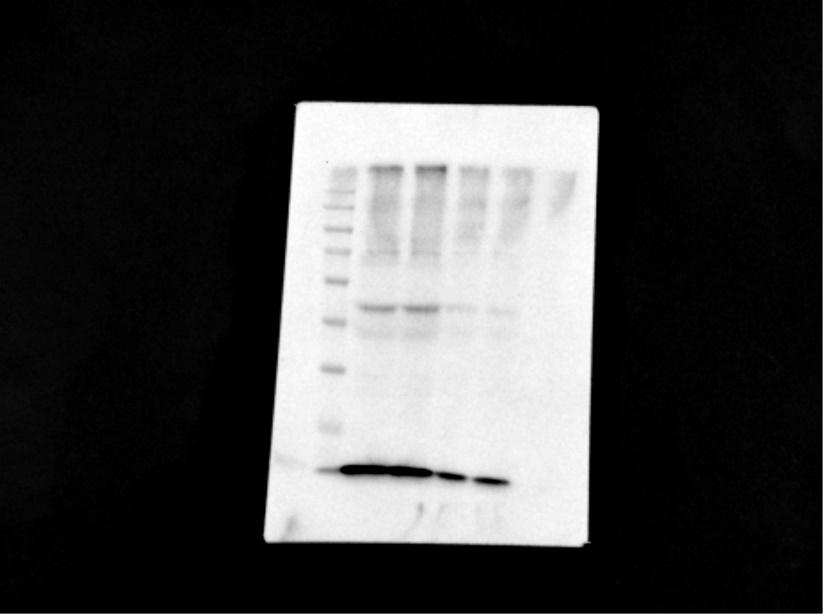


Supplementary Figure 59(Figure 5E-6)


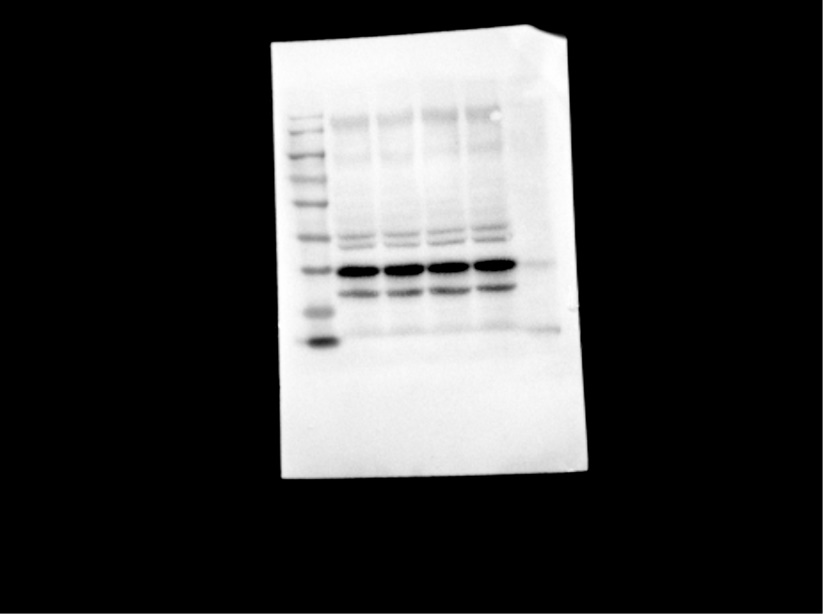


Supplementary Figure 60(Figure 5E-7)


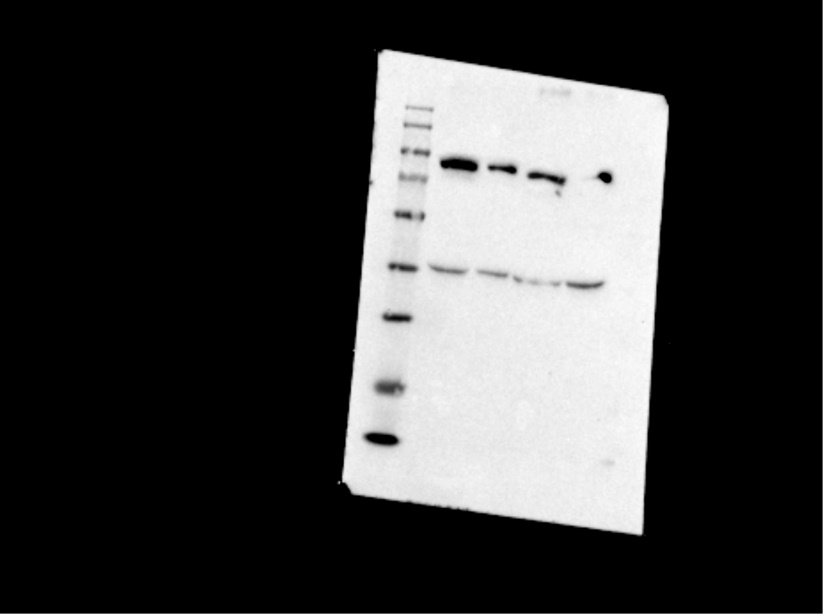


Supplementary Figure 61(Figure 5E-8)


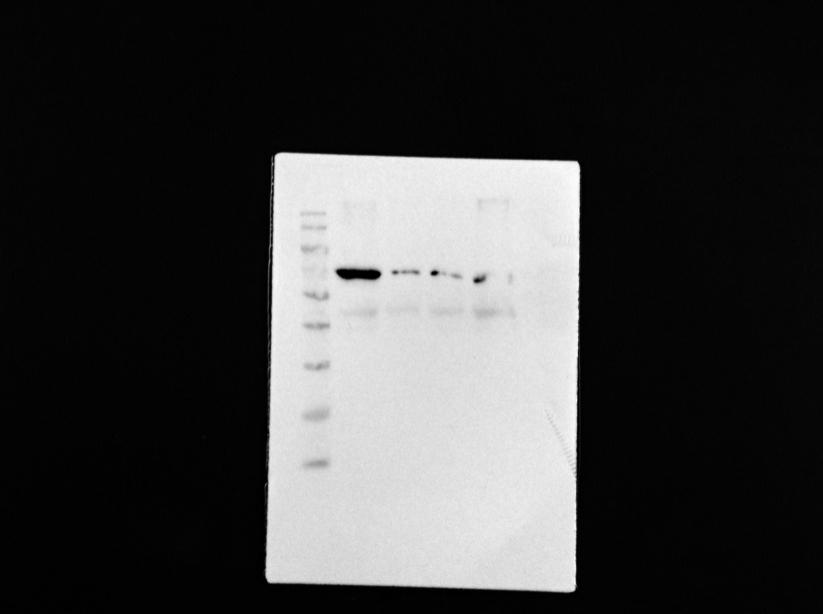


Supplementary Figure 62(Figure 5E-9)


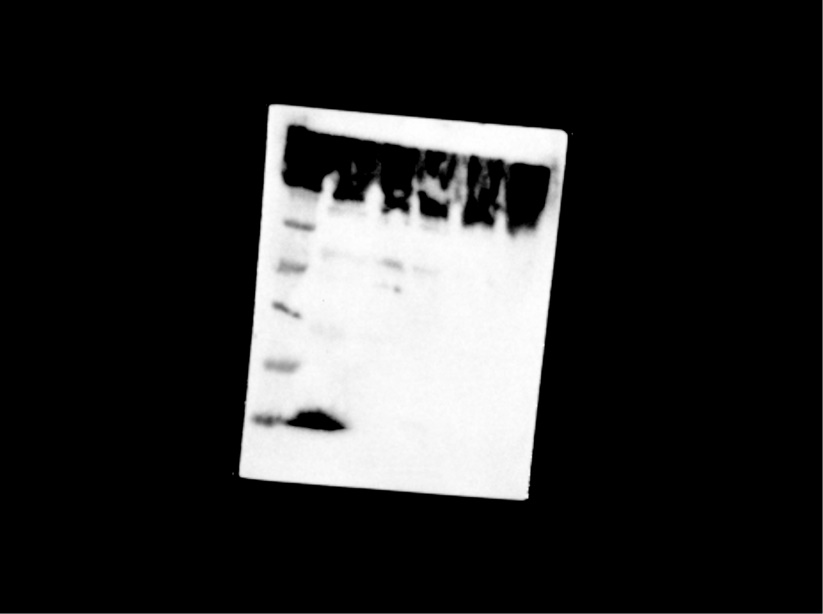


Supplementary Figure 63(Figure 5E-10)


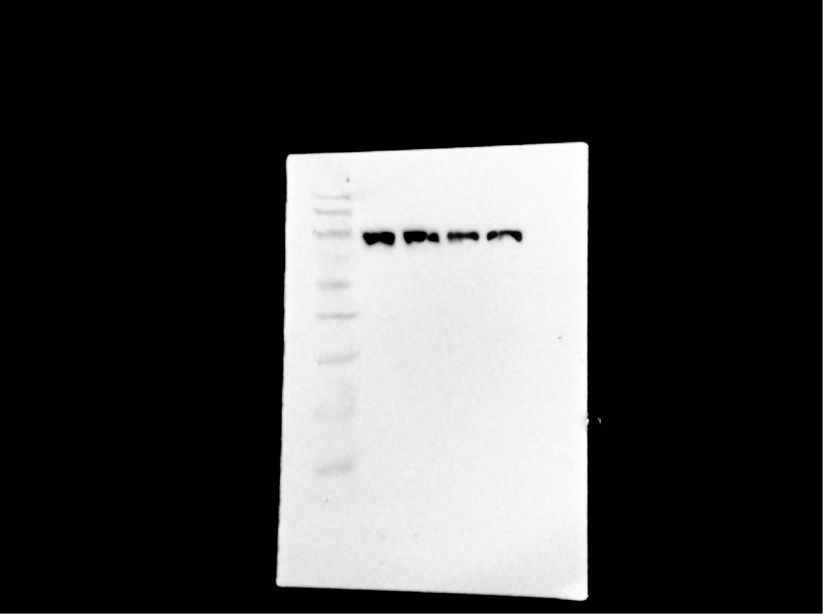


Supplementary Figure 64(Figure 5E-11)


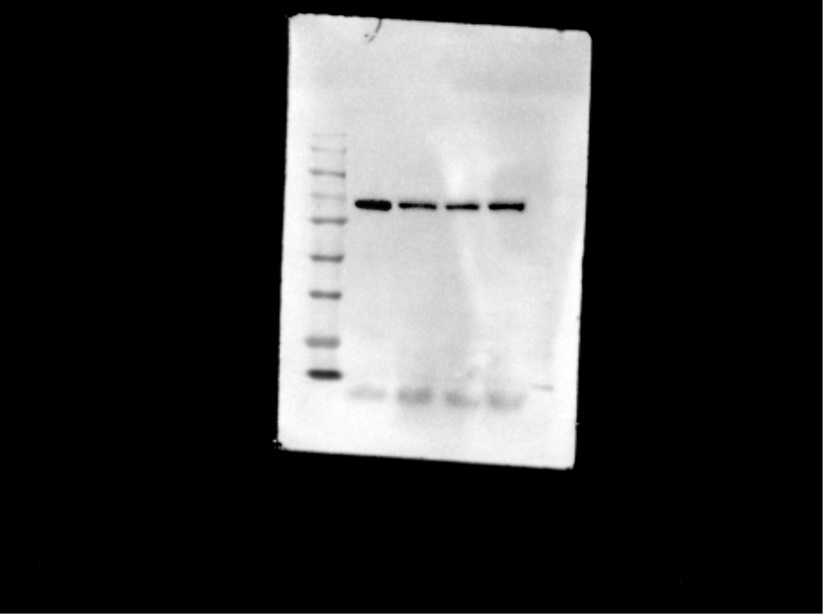


Supplementary Figure 65(Figure 5E-12)


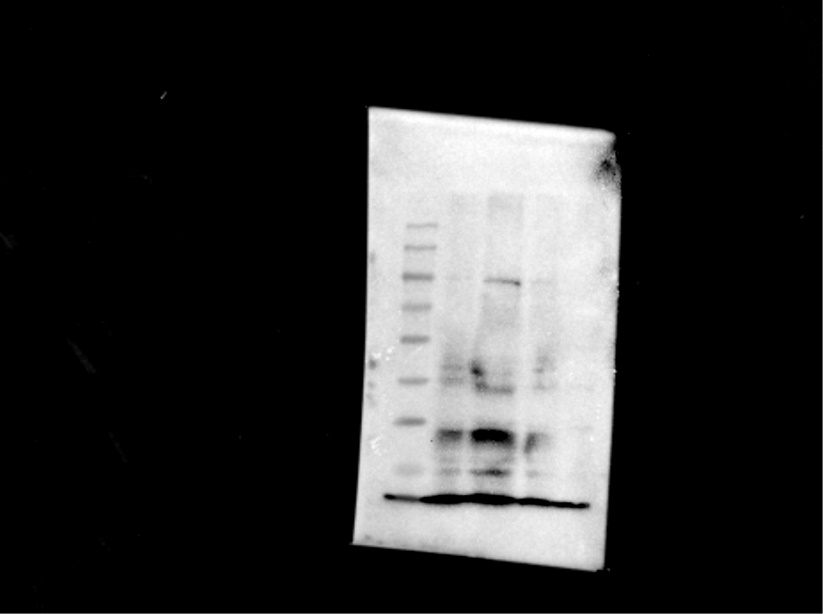


Supplementary Figure 66(Figure 5E-13)


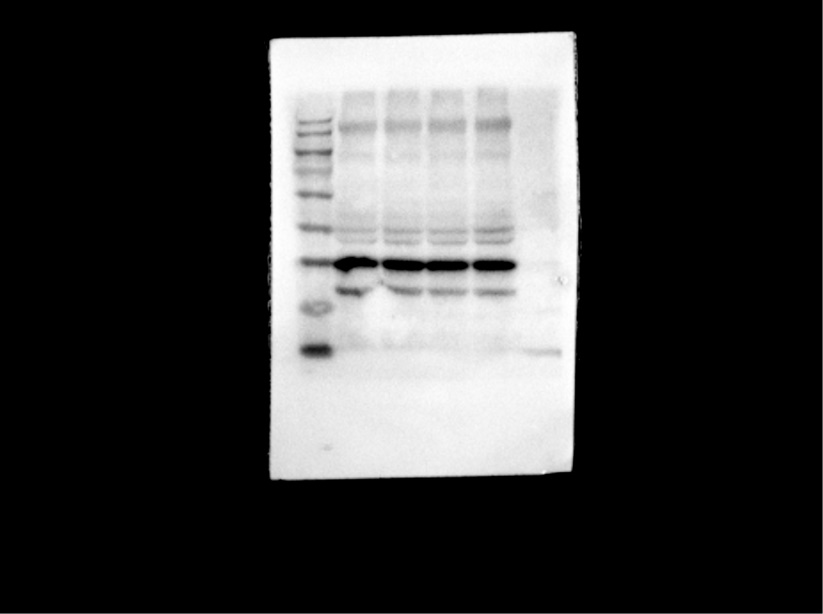


Supplementary Figure 67(Figure 5E-14)


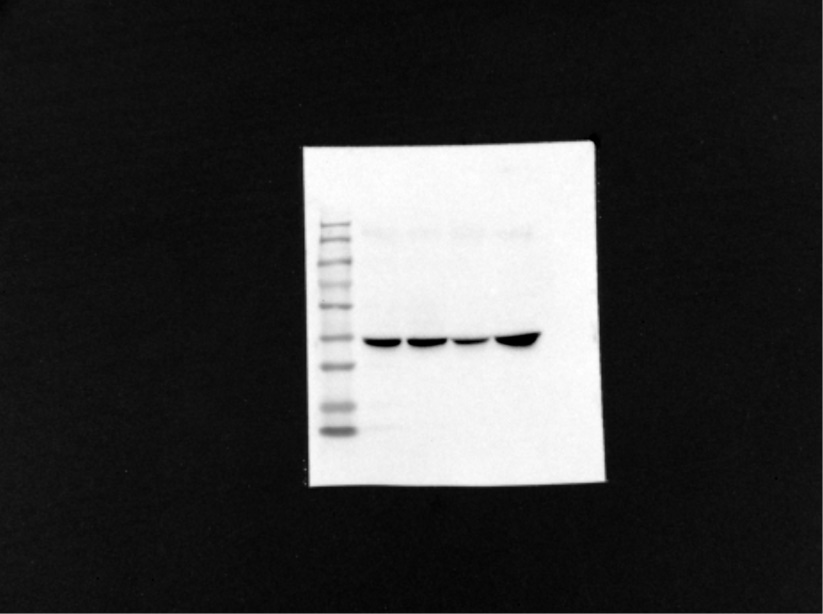


Supplementary Figure 68(Figure 4C-1)


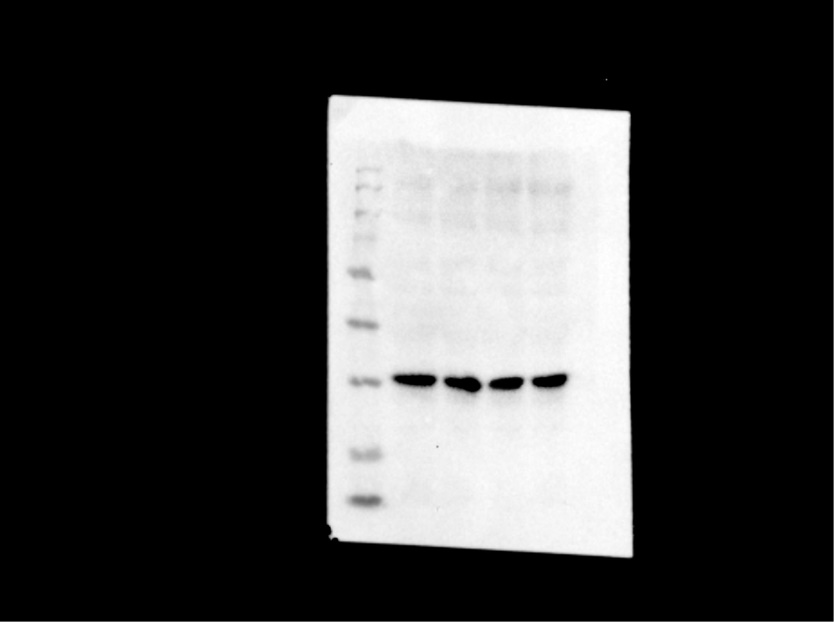


Supplementary figure 69 (Figure 4C-2)


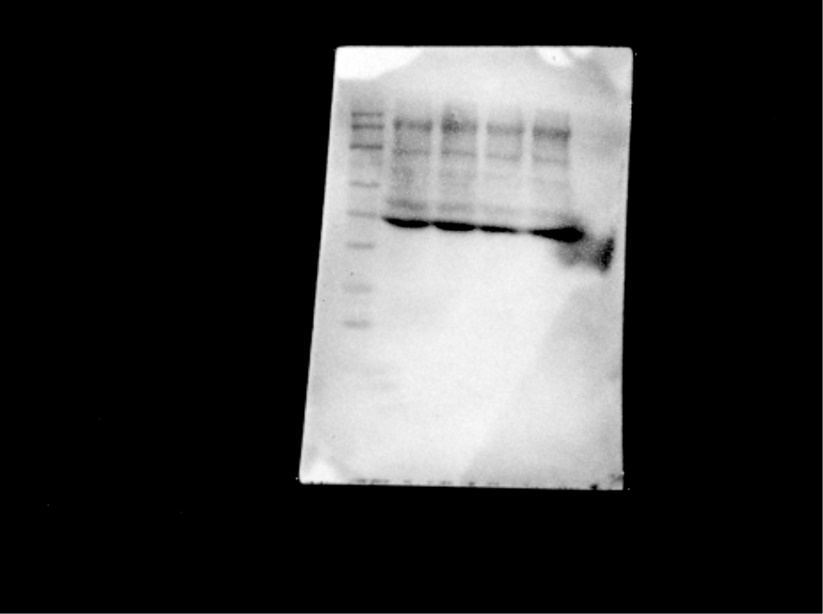


Supplementary figure 70 (Figure 4C-3)


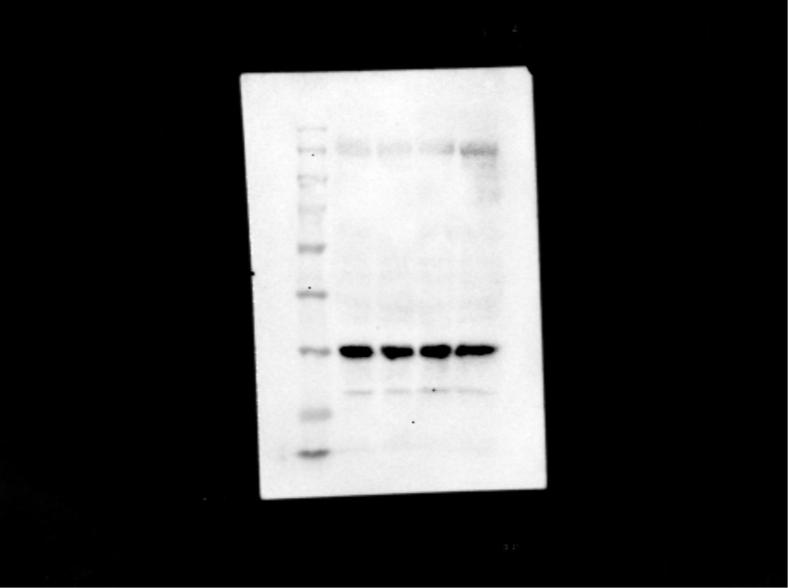


Supplementary figure 71(Figure 4C-4)


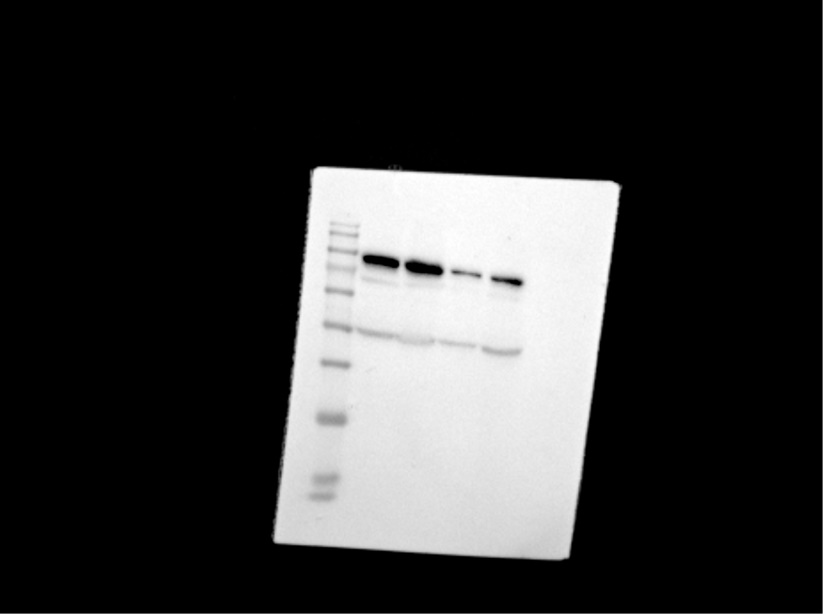


Supplementary figure 72(Figure 6A-1)


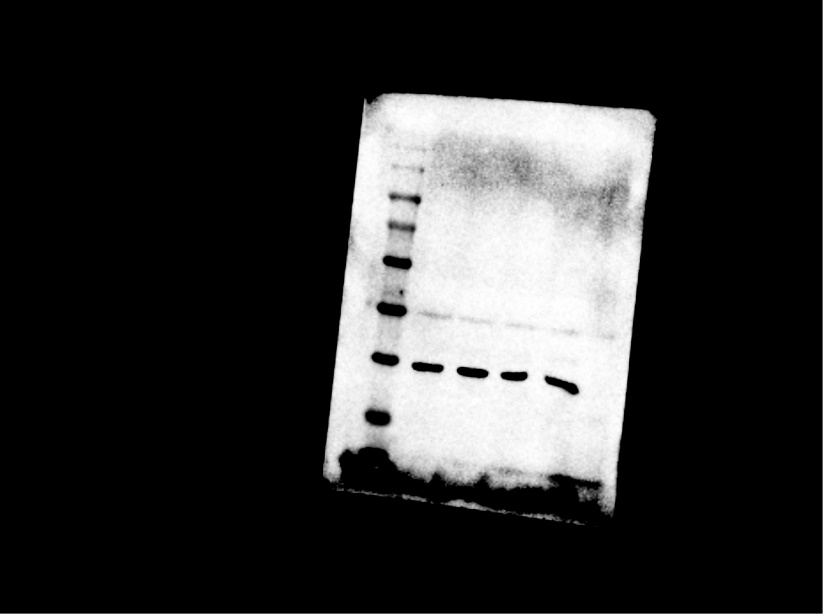


Supplementary figure 73(Figure 6A-2)


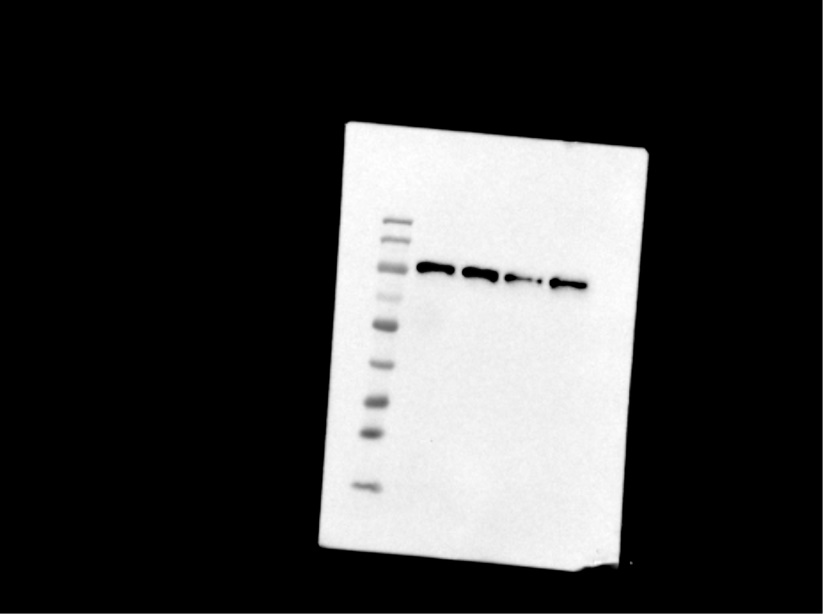


Supplementary figure 74(Figure 6A-3)


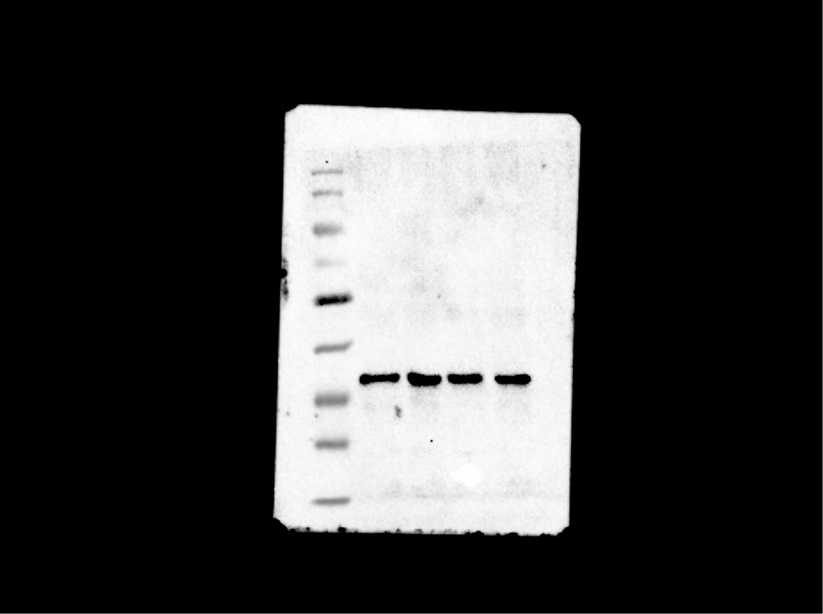


Supplementary figure 75(Figure 6A-4)
